# Self-organized hetero-nanodomains actuating super Li^+^ conduction in glass ceramics

**DOI:** 10.1038/s41467-023-35982-7

**Published:** 2023-02-07

**Authors:** Yantao Wang, Hongtao Qu, Bowen Liu, Xiaoju Li, Jiangwei Ju, Jiedong Li, Shu Zhang, Jun Ma, Chao Li, Zhiwei Hu, Chung-Kai Chang, Hwo-Shuenn Sheu, Longfei Cui, Feng Jiang, Ernst R. H. van Eck, Arno P. M. Kentgens, Guanglei Cui, Liquan Chen

**Affiliations:** 1 Qingdao Industrial Energy Storage Research Institute, Qingdao Institute of Bioenergy and Bioprocess Technology, Chinese Academy of Sciences, Qingdao, 266101 PR China; 2 School of Future Technology, University of Chinese Academy of Sciences, Beijing, 100049 PR China; 3 Institute for Molecules and Materials, Radboud University, 6525 AJ Nijmegen, The Netherlands; 4 School of Materials Science and Engineering, Tianjin University of Technology, Tianjin, 300384 PR China; 5 State Key Laboratory of Microbial Technology, Shandong University, Qingdao, 266237 PR China; 6 Max Plank Institute for Chemical Physics of Solids, Nothnitzer Strasse 40, D-01187 Dresden, Germany; 7 National Synchrotron Radiation Research Center, Hsinchu, Taiwan 30076 Republic of China; 8 Beijing National Laboratory for Condensed Matter Physics, Institute of Physics, Chinese Academy of Sciences, Beijing, 100190 PR China

**Keywords:** Batteries, Batteries

## Abstract

Easy-to-manufacture Li_2_S-P_2_S_5_ glass ceramics are the key to large-scale all-solid-state lithium batteries from an industrial point of view, while their commercialization is greatly hampered by the low room temperature Li^+^ conductivity, especially due to the lack of solutions. Herein, we propose a nanocrystallization strategy to fabricate super Li^+^-conductive glass ceramics. Through regulating the nucleation energy, the crystallites within glass ceramics can self-organize into hetero-nanodomains during the solid-state reaction. Cryogenic transmission electron microscope and electron holography directly demonstrate the numerous closely spaced grain boundaries with enriched charge carriers, which actuate superior Li^+^-conduction as confirmed by variable-temperature solid-state nuclear magnetic resonance. Glass ceramics with a record Li^+^ conductivity of 13.2 mS cm^−1^ are prepared. The high Li^+^ conductivity ensures stable operation of a 220 μm thick LiNi_0.6_Mn_0.2_Co_0.2_O_2_ composite cathode (8 mAh cm^−2^), with which the all-solid-state lithium battery reaches a high energy density of 420 Wh kg^−1^ by cell mass and 834 Wh L^−1^ by cell volume at room temperature. These findings bring about powerful new degrees of freedom for engineering super ionic conductors.

## Abstract (web summary)

The development of Li2S-P2S5 glass ceramics is greatly hampered by the low room temperature lithium conductivity. Here, the authors propose a nanocrystallization strategy to fabricate super lithium conductive glass ceramics.

## Introduction

Sulfide electrolytes have attracted much attention among the all-solid-state battery (ASSB) community in terms of their favorable formability and high Li^+^ conductivity (${\sigma }_{{{Li}}^{+}}$)^1^. Among various kinds of sulfides, Li_2_S-P_2_S_5_ glass ceramics (GCs) are among the first introduced already in 1980^2^. They remain very promising candidates for the simple element composition and mild preparation conditions in comparison to the recently developed thiophosphate Li_10_GeP_2_S_12_ or argyrodite Li_6_PS_5_Cl^3,4^. Yet, the GCs’ ${\sigma }_{{{Li}}^{+}}$ typically lower than 1 mS cm^−1^ at room temperature, greatly hampers their application in ASSBs. To overcome this problem, various attempts have been made to substitute either cations such as Ce^4+^, Sn^4+^, and Sb^5+^ at the P-site, or anions like O^2−^ and Cl^−^ at the S-site^5–8^. However, on the one hand, the complicated element composition resulting from the multi-element doping greatly increases the preparation difficulties to obtain the target products with high purity. Moreover, these heavy doping elements, undoubtedly increase the electrolyte mass, thereby lowering the specific energy. What’s worse, the valence-variable elements, e.g., Sb^5+^, Ce^4+^, can be easily reduced to generate highly resistive phases upon contact with low-voltage anodes.

In 2013, Liu et al. developed a highly conductive GC by a solvent-assisted method. Analysis attributed the anomalous conduction phenomenon to a microstructure-mediated surface conduction mechanism^9^. Although the wet-chemical method is less suitable for large-scale synthesis than the solid-state reaction due to solvent volatilization and toxicity^10^, it is favored to pursue the desirable ${\sigma }_{{{Li}}^{+}}$ through (micro)structure tuning. In reality, the GCs’ ${\sigma }_{{{Li}}^{+}}$ depends highly on the crystallites’ microstructural characteristics like grain size, morphology or distribution, etc^11^. As Maier points out, reducing the crystallite size to the nanoscale has a crucial impact on mass transfer^12^. Nanostructured materials differ from their conventional bulk counterparts by a large fraction of grain boundaries, which are so closely spaced that charge carrier channels for fast migration exist^13^. As a classic example, in the nanostructured hetero-domain (hetero-nanodomain) AgI/Al_2_O_3_^14^_,_ the Ag^+^ conductivity is considerably improved due to the more abundant charge carriers and lower diffusion energy barrier along the grain boundaries compared to the bulk^15^. These scenarios motivate us to present a strategy for nanocrystallization to significantly increase the grain boundary densities and thereby to optimize the GCs’ ${\sigma }_{{{Li}}^{+}}$, which, to the best of our knowledge, has not yet been proposed.

Advanced techniques for obtaining nanocrystals, including molecular beam epitaxy, pulsed laser deposition, and sputtering etc., are not suitable to scale-up production. From an industrial point of view, it is extremely urgent to open a universal way to incorporate nanocrystallites in GCs via solid-state reactions. Inspired by the classic LaMer model in colloid chemistry^16^, we propose here a nucleation-accelerant induced sequential crystallization and heterogeneous growth mechanism: during the solid-state reaction, the nucleation-accelerant reduces the crystal nucleation barrier to induce a short burst of nucleation forming the core nano-grains, on whose crystal faces another crystal subsequently crystallizes and self-organizes into hetero-nanodomains with a large proportion of grain boundaries.

The universality of this mechanism is confirmed through advanced characterization techniques, including synchrotron X-ray diffraction (SXRD), static and magic angle spinning solid-state nuclear magnetic resonance (MAS SSNMR), cryogenic transmission electron microscope (cryo-TEM) and electron holography. Most importantly, the resultant hetero-nanodomains can actuate superior Li^+^-conduction along the grain boundaries, as confirmed by density functional theory (DFT) calculations and variable-temperature SSNMR. Consequently, the highest room temperature ${\sigma }_{{{Li}}^{+}}$ of 13.2 mS cm^−1^ among the previously reported GCs is obtained. Such a high ${\sigma }_{{{Li}}^{+}}$ enables the successful implementation of GCs within a 220 μm-thick composite cathode (44.6 mg cm^−2^ LiNi_0.6_Mn_0.2_Co_0.2_O_2_, NCM622), endowing the resulting ASSB with an excellent energy density of 420 Wh kg^−1^ (by cell mass) and 834 Wh L^−1^ (by cell volume) at room temperature. This approach provides a general framework and universal strategy to design super ionic conductors, which makes the present findings highly relevant for the development of ASSBs.

## Results and discussion

### Sequential crystallization and heterogeneous growth model

**Fig. 1:**
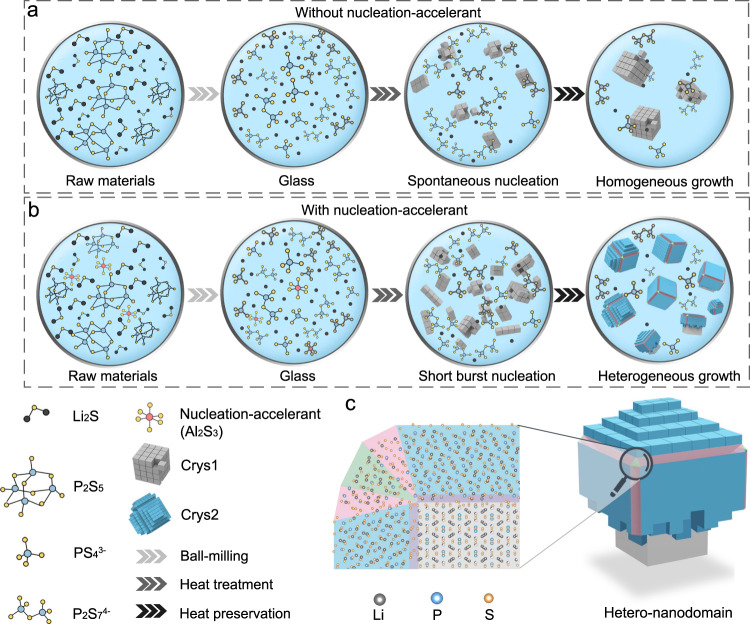
Schematic illustration of the sequential crystallization and heterogeneous growth model (mechanism). GC’s crystallization (**a**) without and (**b**) with nucleation-accelerant. **c** A schematic diagram of 3-dimentional and 2-dimentional self-organized hetero-nanodomain. Light blue area, Crys2 shell nanocrystallites; Grey area, Crys1 core nanocrystallite; Red, violet, and green area, grain boundaries.

**Fig. 2:**
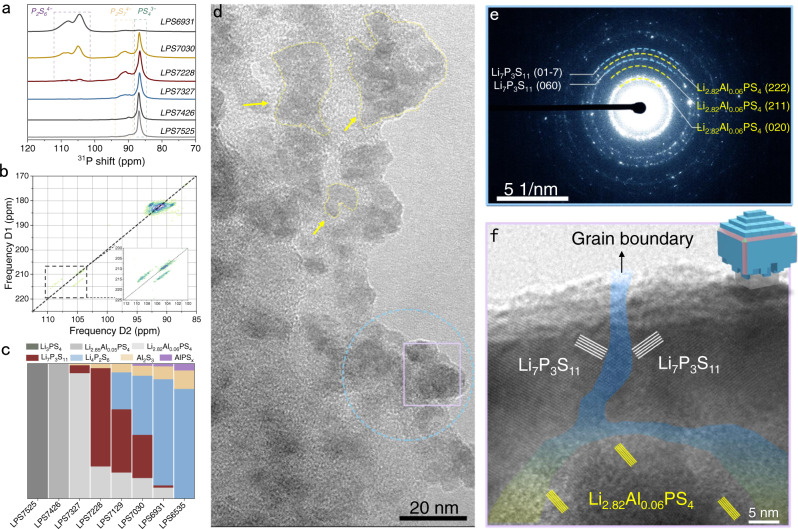
Specific species of Al-GCs and the microstructure of hetero-nanodomains. **a** ^31^P MAS SSNMR spectra of Al-GCs. **b** 2D ^31^P refocused INADEQUATE spectrum of LPS7228. The contour peaks in the 2D spectrum indicate the phosphorous units that are chemically bonded. **c** Bar graph visualizing the species evolution of Al-GCs with various Li_2_S: P_2_S_5_. **d** cryo-TEM, **e** SAED, and **f** HRTEM images of hetero-nanodomains in LPS7228.

The crystallization of glass mainly consists of nucleation and crystal growth. Without disturbances, the nucleus will grow freely to form a crystalline mass (spontaneous nucleation and homogeneous growth, Fig. 1a). If nanocrystallites are desired, the crystallization must be interrupted. Based on the LaMer model, two essential prerequisites must be met to achieve nanocrystallization: short burst nucleation and crystal growth suppression, in which the nucleation-accelerants play an indispensable role^17,18^. Additionally, the solid-state chemistry of the Li_2_S-P_2_S_5_ binary system indicates that the crystalline substances depend on the fraction of Li_2_S or P_2_S_5_, i.e., the P/S ratio^19^. Accordingly, the nucleation-accelerants should have the following functions to induce sequential crystallization and heterogeneous growth to form hetero-nanodomains: 1) substantially lowering the nucleation barrier of crystallite 1 (Crys1) and thus triggering its burst nucleation (Fig. 1b); 2) changing the P/S ratio around the as-formed nuclei to suppress their continuous growth. Therefore, the altered P/S ratio triggers the formation of different crystallite (Crys2). Based on the fact that heterogeneous nucleation is usually kinetically more favorable than homogeneous nucleation^20^, Crys2 tends to nucleate and grow upon Crys1. More importantly, Crys2 growing on different crystal faces of Crys1 shows different orientations, limiting the coalescence of Crys2 to bulk. Hence, under the action of nucleation-accelerant, Crys1 and Crys2 self-organize into hetero-nanodomains.

As indicated in Fig. 1c, within the hetero-nanodomain, the difference in crystal structure between Crys1 and Crys2 result in geometric misfits, that is, heterogeneous grain boundaries, while adjacent Crys2 with different orientations cause geometric misfits of homogeneous grain boundaries. For a perfect hetero-nanodomain, there are 12 grain boundaries (red) resulting from two adjacent Crys2 nanocrystallites, 8 grain boundaries (green) resulting from three adjacent Crys2 nanocrystallites, and 6 grain boundaries (violet) between Crys1 and Crys2 nanocrystallites. The large proportion of grain boundaries occurring in the self-organized hetero-nanodomains are predicted to significantly enhance the ${\sigma }_{{{Li}}^{+}}$ of GCs.

Guided by this model, a series of nucleation-accelerants including Al_2_S_3_, Ga_2_S_3_, and SiS_2_ are screened to verify the universality of this mechanism. All of these nucleation-accelerants can induce the sequential crystallization and heterogeneous growth to self-organize into hetero-nanodomains in GCs, significantly improving the ${\sigma }_{{{Li}}^{+}}$ to 10 mS cm^−1^ at room temperature. It is believed that, in addition to the three nucleation-accelerants provided in this work, more nucleation-accelerants will be discovered based on this universal mechanism. In the following results and discussion, we focus on Al_2_S_3_ as the nucleation-accelerant to validate the model (mechanism), while the results for Ga_2_S_3_ and SiS_2_ can be found in the Supplementary Information.

### Species identification of Al_2_S_3_-tuned GCs (Al-GCs)

As indicated in Table 1, the Al-GCs are obtained by adding Al_2_S_3_ nucleation-accelerant to regulate the molar ratio of Li_2_S: P_2_S_5_ from 75: 25 to 65: 35, the ratio ranges commonly studied^19^. The raw materials are mixed by mechanical grinding and subsequently subjected to a heat treatment. Supplementary Fig. 1a and Supplementary Fig. 1b outline the obtained Al-GCs with different phase compositions identified by SXRD and Raman spectra. Conclusively, as visualized in Supplementary Fig. 1h, at a decreasing Li_2_S: P_2_S_5_ ratio from 75: 25 to 65: 35, the phase composition undergoes the crystal structure transformation of Li_3_PS_4_ analog phase → Li_7_P_3_S_11_ analog phase → Li_4_P_2_S_6_ analog phase.

**Table 1:** Molar ratio of the reagents (Li_2_S, P_2_S_5_, Al_2_S_3_) in Al-GCs and the corresponding denotation

Li_2_S	P_2_S_5_	Al_2_S_3_	Ratio of (Li_2_S: P_2_S_5_)	Denotation
56.25	18.75	0	75:25	LPS7525
55.5	19.5	1	74:26	LPS7426
54.75	20.25	2	73:27	LPS7327
54	21	3	72:28	LPS7228
53.25	21.75	4	71:29	LPS7129
52.5	22.5	5	70:30	LPS7030
51.75	23.25	6	69:31	LPS6931
48.75	26.25	10	65:35	LPS6535

The subtle changes in the local structure of Al-GCs are also detected by ^31^P MAS NMR spectra. As shown in Fig. 2a, for both LPS7525 and LPS7426, one single resonance at 87 ppm with a small shoulder at 89 ppm is detected, indicating the presence of isolated PS_4_^3−^ polyhedra. For LPS7327, in addition to the 89 ppm peak, another peak at 91 ppm is observed simultaneously. The resonances at 91 ppm can be attributed to P_2_S_7_^4−^^21^. Further decreasing Li_2_S, P_2_S_6_^4−^ units (104 and 108 ppm) are detected and continuously increasing with decreasing Li_2_S content, which is in good accordance with the SXRD and Raman results. To further corroborate the bond connectivity in the P − S units, a 2D ^31^P − ^31^P INADEQUATE NMR experiment was performed for LPS7228. The 2D experiment is based on *J*-coupling which allows us to probe P − P and/or P − S − P bond connectivity. Figure 2b clearly shows that an intense peak located at 91 ppm attributed to the P − S − P bond in P_2_S_7_^4−^ is observed. Interestingly, there are three peaks at (104, 208), (105, 213) and (108, 213) stemming from the P − P bond in P_2_S_6_^4−^. The peak on the diagonal arises from the P − P correlation between two identical P sites, while the width of peaks parallel to the diagonal hints at the slightly different P environment in P_2_S_6_^4−^ units. This could be explained by the disordered conformation of [P_4/2_S_6_]^4−^ anion in which only two out of four possible P sites are simultaneously occupied^22^.

A more detailed characterization of the evolution of species in Al-GCs based on ^27^Al and ^6^Li SSNMR spectra can be found in a separate publication^23^, this suggests that Al is first introduced to partially occupy the vacant LiS_6_ sites in Li_3_PS_4_ to form Li_3–3*x*_Al_*x*_PS_4_, and the solid solubility is 0.06. Beyond the solid solubility limit, lithium-depleted thiophosphates, such as Li_7_P_3_S_11_ and Li_4_P_2_S_6_, begin to form with decreasing Li_2_S content. The evolution of species in Al-GCs is visualized in Fig. 2c.

### Hetero-nanodomains

The solid chemistry of the Li_2_S-P_2_S_5_ binary system indicates that the crystalline substance depends strongly on the P/S ratio in the starting materials (Supplementary Fig. 4a). For example, when the molar ratio of Li_2_S to P_2_S_5_ is 75: 25 (P/S = 0.25), pure crystalline Li_3_PS_4_ can be obtained, with all sulfur atoms are in PS_4_^3−^ tetrahedra. Upon decreasing the ratio to 70: 30 (P/S = 0.273), a pure Li_7_P_3_S_11_ phase is deposited consisting of PS_4_^3−^ isolated and corner-sharing P_2_S_7_^4−^ tetrahedra with a 1: 1 molar ratio. Decreasing the Li_2_S fraction further to 67 mol% (P/S = 0.333), pure Li_4_P_2_S_6_ phase crystallizes, yielding P_2_S_6_^4−^ dumbells.

Because both P_2_S_7_^4−^ and P_2_S_6_^4−^ derive from PS_4_^3−^ (Supplementary Fig. 4b), in this work, the P/S ratio of all sample reagents are fixed to 0.25, where the P source is P_2_S_5_ and the S sources are Li_2_S, P_2_S_5_ and Al_2_S_3_. Both SXRD and ^27^Al MAS SSNMR results suggest that Al_2_S_3_ can participate in the solid-state reaction to form Al-doped Li_3_PS_4_, i.e., Li_3−3*x*_Al_*x*_PS_4_, while the ^27^Al MAS SSNMR results further indicate that the maximum of the Al doping content, i.e., the solubility limit of Al, is only 0.06. Below this threshold, all the Al_2_S_3_ is reactive and thus the P/S ratio is constant 0.25 to generate Li_3−3*x*_Al_*x*_PS_4_. Beyond the solubility limit, the S source from the residual Al_2_S_3_ is nonreactive and as a result the P/S ratio is higher than 0.25. In these cases, Li_2.82_Al_0.06_PS_4_ (P/S = 0.25), Li_7_P_3_S_11_ (P/S = 0.273) and Li_4_P_2_S_6_ (P/S = 0.333) crystallize sequentially based on the ∆G_r_ (Gibbs free energy change) difference: ∆G_r(Li3−3*x*Al*x*PS4)_ (−138.38 meV atom^−1^) <∆G_r(Li7P3S11)_ (−87.27 meV atom^−1^) <∆G_r(Li4P2S6)_ (12.81 meV atom^−1^).

Take LPS7228 for example: 72 mol% Li_2_S, 4 mol% Al_2_S_3_, and 28 mol% P_2_S_5_ first react to form 27.1 wt % Li_2.82_Al_0.06_PS_4_ due to the lowest ∆G_r_, which indicates Al_2_S_3_ substantially lowers the nucleation barrier of Li_2.82_Al_0.06_PS_4_ and thus triggers its burst nucleation. By theoretically excluding the unreacted Al_2_S_3_, the remaining Li_2_S and P_2_S_5_ lead to a P/S ratio of 0.267, just satisfying the formation condition for forming Li_7_P_3_S_11_ (P/S ratio, 0.273). Details of the calculation can be found in the Supplementary Information. Hence, another function of the nucleation-accelerant is demonstrated, that is, changing the P/S ratio of the as-formed Li_2.82_Al_0.06_PS_4_ crystallite to suppress its continuous growth, while the changed P/S ratio triggers the formation of Li_7_P_3_S_11_. This sequential crystallization phenomenon induced by Al_2_S_3_ is experimentally confirmed via in situ temperature-dependent XRD analysis. As shown in Supplementary Fig. 5, Li_2.82_Al_0.06_PS_4_ starts to crystallize once the furnace is heated to 300 °C while Li_7_P_3_S_11_ crystallizes only after being held at 300 °C for 40 min. Following that, the XRD spectra show that the intensity of all peaks steadily rises with heat treatment duration, implying that the degree of crystallinity increases continuously. Meanwhile, no other impurities emerge during annealing.

The hetero-nanodomains resulting from the sequential crystallization and heterogeneous growth are then investigated by cryo-TEM. As shown in Fig. 2d, a large amount of nanodomains (the dark area) with a size of 20–40 nm are observed, indicating the burst nucleation. To further characterize these nanodomains, selected area electron diffraction (SAED) is first performed for the region marked by an azury circle in Fig. 2d. The SAED patterns displayed in Fig. 2e exhibit Debye-Scherrer rings with a halo pattern, indicating that LPS7228 contains both Li_2.82_Al_0.06_PS_4_ and Li_7_P_3_S_11_ crystallites, agreeing well with the SXRD results. High resolution TEM (HRTEM) of the pink square within the SAED area is displayed in Fig. 2f, the HRTEM image reveals clear lattices with interplanar distances of 0.332 nm (marked yellow) and 0.368 nm (marked white), matching well with the ${{{{{{\rm{d}}}}}}}_{121}$ and ${{{{{{\rm{d}}}}}}}_{1\bar{3}0}$ spacing of Li_2.82_Al_0.06_PS_4_ and Li_7_P_3_S_11_, respectively^24,25^. Interestingly, this image reveals that the Li_2.82_Al_0.06_PS_4_ nanocrystallites are closely surrounded by Li_7_P_3_S_11_ nanocrystallites to form Li_7_P_3_S_11_@Li_2.82_Al_0.06_PS_4_ hetero-nanodomains. DFT calculations also suggest the preferential nucleation and growth of Li_7_P_3_S_11_ crystallites on Li_2.82_Al_0.06_PS_4_ crystal faces (Supplementary Fig. 6). In such hetero-nanodomains, the proportion of 5 nm-wide grain boundaries resulting from the geometric mismatch between the Li_2.82_Al_0.06_PS_4_ core nanocrystallites and the Li_7_P_3_S_11_ shell nanocrystallites, or among the adjacent Li_7_P_3_S_11_ nanocrystallites with different orientations, increases substantially.

### Li^+^-conduction

**Fig. 3:**
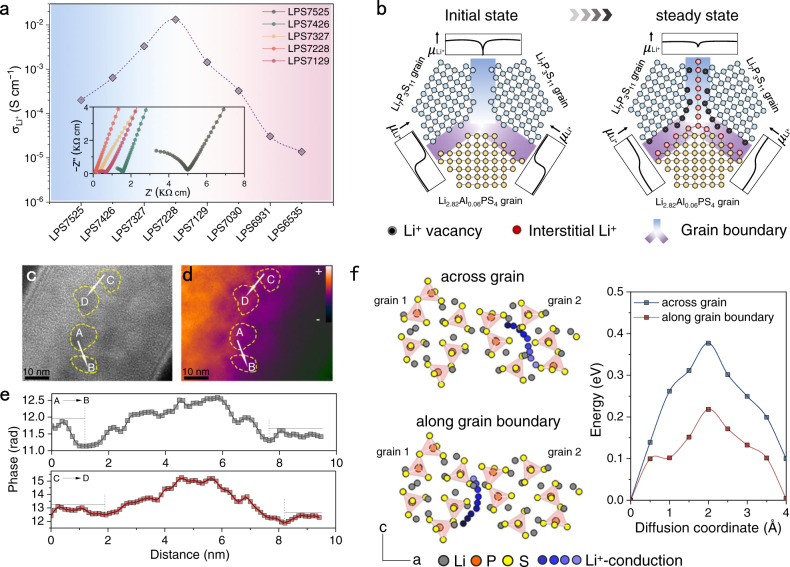
The super Li^+^-conduction. **a** ${\sigma }_{{{Li}}^{+}}$ at room temperature of various Al-GCs in the cold-pressed state. EIS plots are shown in the inset. **b** Schematic diagram of the carrier redistribution within Li_7_P_3_S_11_@Li_2.82_Al_0.06_PS_4_ hetero-nanodomain driven by *μ*_*Li*_^*+*^. **c**–**e** Electron holography observation of LPS7228: **c** Initial hologram image. **d** Reconstructed phase image. Inset color bar is defined as the relative magnitude of th**e** radius. **e** Phase profiles along the white arrow from A to B and C to D. **f** Schematic diagram of Li^+^ migration along grain boundary and across grain with the corresponding diffusion energy (activation energy).

To gain in-depth insight into the correlation between microstructure and conductivity, the trend in ${\sigma }_{{{Li}}^{+}}$ as determined from electrochemical impedance spectroscopy (EIS) is plotted. According to Fig. 3a and Supplementary Fig. 7, a value of 0.20 mS cm^−1^ is reached for LPS7525 at room temperature and increases to 0.64 mS cm^−1^ for LPS7426. With decreasing Li_2_S: P_2_S_5_ ratio, ${\sigma }_{{{Li}}^{+}}$ increases to 3.3 mS cm^−1^ for LPS7327, and even reaches a maximum of 13.2 mS cm^−1^ for LPS7228. Further reduction in the Li_2_S: P_2_S_5_ ratio reduces ${\sigma }_{{{Li}}^{+}}$ to 1.43 (LPS7129), 0.33 (LPS7030), 0.03 (LPS6931), and 0.014 mS cm^−1^ (LPS6535). Supplementary Table 13 lists the ${\sigma }_{{{Li}}^{+}}$ for GCs prepared using the solid-state reaction method reported in the literature. Clearly, sample LPS7228 studied in this work reaches the highest value reported so far.

SXRD analysis suggests that LPS7228 consists of Li_2.82_Al_0.06_PS_4_ (27.1 wt%) and Li_7_P_3_S_11_ (72.9 wt%) with a small quantity of Al_2_S_3_ residues, Fig. 2c. According to the EIS results in Supplementary Fig. 10, ${\sigma }_{{{Li}}^{+}}$ of the as-prepared Li_2.82_Al_0.06_PS_4_ and Li_7_P_3_S_11_ is estimated to be 0.94 and 1.4 mS cm^−1^, respectively. In addition, the factual composite of 27.1 wt% Li_2.82_Al_0.06_PS_4_ and 72.9 wt% Li_7_P_3_S_11_ exhibits a ${\sigma }_{{{Li}}^{+}}$ of 1.01 mS cm^−1^. All are much lower than the value of 13.2 mS cm^−1^ for LPS7228. Based on this observation, the high value of ${\sigma }_{{{Li}}^{+}}$ for LPS7228 is believed to originate from the make-up of the hetero-nanodomains.

As recently suggested^26^, when two phases come into contact, ions (carriers) will redistribute along the grain boundaries because of the chemical potential (*μ*) difference to form defect-rich regions that allow the carriers to migrate readily. For the Li_7_P_3_S_11_/Li_2.82_Al_0.06_PS_4_ heterojunction, DFT calculations suggest that *μ*_*Li*_^*+*^ of Li_7_P_3_S_11_ (−3.25 eV) is higher than that of Li_2.82_Al_0.06_PS_4_ ( − 3.35 eV). Thus, a defect-rich region in which Li^+^ vacancies accumulate at the edge of the grain boundaries near Li_7_P_3_S_11_ and Li^+^ ions accumulate at the edge near Li_2.82_Al_0.06_PS_4_, as shown in Fig. 3b. In Li_7_P_3_S_11_/Li_7_P_3_S_11_ homojunction, *μ*_*Li*_^*+*^ is usually lower in the grain boundary core than at the edges^27^. Hence, Li^+^ moves spontaneously from the grain boundary edges to the core, forming a symmetric region with abundant Li^+^ ions in the grain boundary core sandwiched between Li^+^ vacancies on both grain boundary edges.

Experimental evidence of carrier redistribution is provided by off-axis electron holography because carrier redistribution causes a built-in potential change^28^. First, an initial electron hologram is acquired using an objective spherical aberration corrected TEM. Then, the reconstructed phase image is separated. In the absence of any magnetic field, the projected phase shift of the electron wave caused by electrostatic fields within the sample can be expressed simply as:

1 $$\varDelta \varphi (x,\,y)={C}_{E}\cdot \int _{{{{{{\rm{path}}}}}}}{V}_{tot}(x,\,y,\,z)dz\, \approx \,{C}_{E}\cdot t(x,\,y)\cdot {V}_{tot}(x,\,y)$$

where *z* is the optical axis, *C*_*E*_ is an electron-energy-dependent interaction constant with the value of 0.01009 rad V^−1^ nm^−1^ for 80 kV electrons, *V*_*tot*_(*x*, *y*) is the average of the potential *V*_*tot*_(*x*,*y*,*z*) through the specimen, *t*(*x*, *y*) is the sample thickness at a given position. By using Eq. (1), the phase shift measured by holography can be directly related to the potential distribution of the sample^29^.

As shown in Fig. 3c, a large number of hetero-nanodomains can be clearly distinguished by the dark regions, among which are the grain boundaries. Phase profiles are extracted along the white arrow (from A to B and C to D, Fig. 3e) in the reconstructed phase image (Fig. 3d). The local potential distribution is highlighted in a color-density plot. As observed from the direction of the white arrow, there is an abrupt decrease in potential at the outer edges of the two adjacent hetero-nanodomains followed by a potential increase. Correspondingly, the grain boundary edges are enriched with Li^+^ vacancies resulting in a low potential, while the core potential increases sharply due to the enrichment of Li^+^ ions. This is a very direct observation of the carrier redistribution as described by our model (Fig. 3b).

With the large fraction of carrier-enriched grain boundaries, enhanced Li^+^ conduction is expected, which is probed by recording static variable-temperature ^7^Li spectra and NMR relaxometry^23^. In particular, the relaxation time in the rotating frame T_1ρ_ is employed to probe Li^+^ dynamics in the kHz regime that is associated with long-range Li^+^ diffusion. It seems that only one motional process is present in LPS7525 with activation barrier of 0.13 eV. This hardly changes for LPS7426. We observe a very flat slope in the temperature-dependent relaxivity curve of LPS7228, meaning the activation barrier is substantially lowered. The curve shows multiple slopes, however, and no well-defined maximum, implying that there is a distribution of multiple diffusion pathways, as is expected for hetero-nanodomains consisting of several phases. Moreover, DFT simulations are performed to investigate Li^+^ diffusion barriers (activation energy, Fig. 3f) along grain boundaries or across nano-grains. It is calculated that the activation energy for Li^+^ diffusion along grain boundaries is 0.21 eV, about 2 times lower than that of across nano-grains, 0.37 eV. According to the Nernst-Einstein Eq. (2) and the Arrhenius Eq. (3), both the enhancement of carriers’ concentration and a low activation energy lead to an excellent ${\sigma }_{{{Li}}^{+}}$ of 13.2 mS cm^−1^ at room temperature.

2 $${\sigma }_{L{i}^{+}}=\frac{N\cdot D\cdot {e}^{2}}{{k}_{B}\cdot T}$$

3 $$D={D}_{0}\cdot {e}^{-\frac{{E}_{a}}{R\cdot T}}$$

where *N* is the number of charge carriers; *D*, the self-diffusion coefficient; *e*, the electron charge; *k*_*B*_, the Boltzmann constant; *T*, the Kelvin temperature; *D*_*0*_, temperature-independent diffusion coefficient; *E*_*a*_, activation energy; *R*, the gas constant.

### Demonstration in lithium metal ASSB

**Fig. 4:**
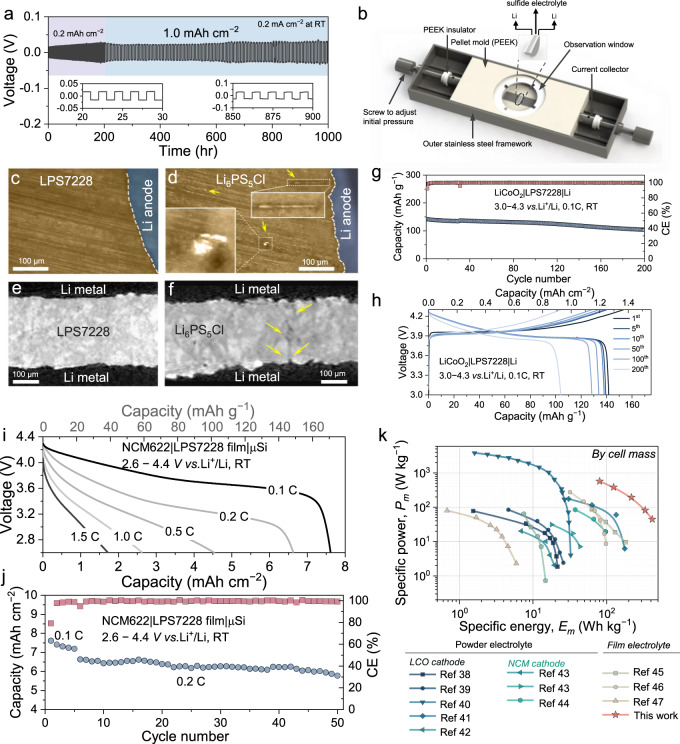
Demonstration of LPS7228 in ASSBs. **a** Galvanostatic cycling of the Li|LPS7228 | Li cell at a current density of 0.2 mA cm^−2^ and areal specific capacities of 0.2 or 1.0 mAh cm^−2^ at room temperature. **b** Schematic view of the home-made device for operando optical microscopy observation. Top-down optical observation of (**c**) Li|LPS7228 | Li and (**d**) Li|Li_6_PS_5_Cl | Li symmetric cells after 10 h cycling. Sliced images of the reconstructed tomographic volumes of (**e**) Li|LPS7228 | Li and (**f**) Li|Li_6_PS_5_Cl | Li symmetric cells after 10 h cycling. **g** Cycle behavior and (**h**) charge-discharge curves of LiCoO_2_ | LPS7228 | Li ASSB at room temperature. **i** The C-rate performance and (**j**) cycle performance of the NCM622 | LPS7228 film|μSi ASSB at room temperature. **k** Ragone plots for cells employing sulfide electrolytes cycled at room temperature. The *E*_*m*_ and *P*_*m*_ are delivered during discharging, normalized by cell mass.

Lithium metal is a promising anode material for high-energy-density ASSBs because of its high theoretical capacity (3860 mAh g^−1^) and low electrochemical potential (−3.04 V vs. the standard hydrogen electrode)^30^. The electrochemical performance of lithium metal ASSB depends strongly on the compatibility between electrolyte and lithium metal. Thereby, the stability of LPS7228 against lithium metal anode is firstly evaluated in symmetric Li−Li cells at room temperature. As shown in Fig. 4a and Supplementary Fig. 16, at a current density of 0.2 mA cm^−2^, the overpotential gradually increases from 15.7 to 28 mV during the first 150 h, which can be attributed to the formation of stabilized interface between lithium metal and LPS7228 as discussed below. The nearly unchanged overpotential after 150 h proves that the in situ formation of the interface is self-terminating, rendering the Li|LPS7228 | Li symmetric cell stable cycling for over 1400 h with specific areal capacity of 0.5 mAh cm^−2^, and over 1000 h with a specific areal capacity of 1.0 mAh cm^−2^. The stability and surface capacity are adequate for high-performance lithium metal ASSBs^31–33^.

To reveal the stable lithium plating/stripping behavior, a device (Fig. 4b) for operando optical microscopy observation was designed in house. For a fair comparison, the most common Li_6_PS_5_Cl is adopted as the contrastive electrolyte. Supplementary Fig. 17 displays a flat voltage plateau of 50 mV for over 10 hrs at a current density of 0.5 mA cm^−2^ for Li|LPS7228 | Li, while the cell using Li_6_PS_5_Cl survives only 28 min with a short circuit appearing afterwards. Cross-sectional snapshots, taken after 10 hrs (Fig. 4c) show an integrated LPS7228 layer without cracks or lithium dendrites inside, which is also verified by X-ray microtomography (Fig. 4e). Besides, the interface between LPS7228 and lithium metal anode is very smooth. In contrast, lithium metal granules, lithium dendrites, and spiky interface between Li_6_PS_5_Cl and the lithium metal anode are clearly observed in Fig. 4d, f and Supplementary Fig. 18. According to X-ray photoelectron spectroscopy (XPS) analysis and DFT calculations, the stable Li polarization behavior can be attributed to the Li−Al alloy formed in situ to uniformize the Li^+^ flux during the Li plating/stripping processes^6,34^. Detailed discussion can be found in the Supplementary Information related to Supplementary Fig. 19.

The high ${\sigma }_{{{Li}}^{+}}$ and good anodic stability of LPS7228 against lithium metal suggest its very promising potential for direct application (without a buffer layer between the electrolyte and lithium metal anode or using a modified lithium metal anode) in lithium metal ASSBs. With a LiNbO_3_-coated LiCoO_2_ cathode (areal loading 8.92 mg cm^−2^), lithium metal ASSBs with Li_3_PS_4_, Li_6_PS_5_Cl, Li_10_GeP_2_S_12_ (LGPS) and LPS7228 electrolytes are demonstrated for a fair comparison. As shown in Supplementary Fig. 21, the LiCoO_2_ | Li ASSB using Li_3_PS_4_ is only stable for the first 3 cycles. After then, the cell shows severe overcharging related to dendrite growth during charging^35^. Similarly, the LiCoO_2_ | Li ASSB with Li_6_PS_5_Cl survives only 15 cycles, followed by a sudden voltage-drop due to lithium dendrite growth within Li_6_PS_5_Cl (Fig. 4d, f). Rapid capacity decay is observed for LiCoO_2_ | LGPS | Li, mainly attributed to the notorious side reactions between lithium metal and LGPS, resulting in highly resistive products^36^.

By a huge contrast, a lithium metal ASSB with LPS7228 demonstrates a more stable cycling performance (Fig. 4g, h). Specifically, a reversible specific discharge capacity of 141.7 mAh g^−1^ is achieved for the first cycle in the voltage range of 3.0 − 4.3 V with a high CE of 92.4%, due to the excellent stability of Li/LPS7228 interface. After 100 cycles, 90.6% capacity is retained, indicating superior interface compatibility. Remarkably, a reversible specific capacity of 104.1 mAh g^−1^ is maintained after 200 cycles, showing a good retention of 73.5% with an average CE of 99.8%, suggesting highly reversible Li^+^ intercalation/deintercalation. Compared to other reported lithium metal ASSBs with the same configuration (Supplementary Table 14), the lithium metal ASSB reported in this work performs best.

### Demonstration in high energy ASSB

To assemble high specific energy (*E*_*m*_) ASSB, a free-standing and flexible film with a thickness of 110 μm is prepared by rubbing LPS7228 powders with polytetrafluoroethylene binder (Supplementary Fig. 24a–c), which has a high room temperature ${\sigma }_{{{Li}}^{+}}$ of 10.3 mS cm^−1^. Microsilicon (μSi) is used as the anode (Supplementary Fig. 24d) due to its high specific capacity exceeding 3500 mAh g^−1^ and excellent C-rate performance^37^. As shown in Supplementary Fig. 24e, the battery is constructed by a 220 μm-thick composite cathode containing 80 wt% LiNbO_3_@NCM622 with an areal loading of 44.6 mg cm^−2^, a LPS7228 film, and a 20 μm-thick μSi anode (N/P = 1.21). At a charge rate of 0.1 C (1 C = 180 mAh g^−1^) and room temperature, an initial discharge capacity of 171 mAh g^−1^ is obtained, corresponding to an excellent areal capacity of 7.62 mAh cm^−2^ and an *E*_*m*_ of 420 Wh kg^−1^ by cell mass. Encouragingly, a high value of 6.63 mAh cm^−2^ corresponding to an *E*_*m*_ of 336 Wh kg^−1^ is still obtained when increasing the C-rate to 0.2 C and cycling stably over 50 cycles with an average CE of 98.1% (Fig. 4j). As included in Fig. 4k, the Ragone plots indicate that compared with the previously reported high energy ASSBs, our battery demonstrates a much higher *E*_*m*_ at a fixed specific power (*P*_*m*_) or vice versa^38–47^, empowering our work an important potential for future development of electric vehicle batteries with both high *E*_*m*_ and *P*_*m*_.

In summary, this work proposes a sequential crystallization and heterogeneous growth mechanism to drive crystallites nanocrystallization during the solid-state reaction. The resulting self-organized hetero-nanodomains, composed of a considerable number of grain boundaries, can actuate super Li^+^-conduction, leading to a high room temperature ${\sigma }_{{{Li}}^{+}}$ of 13.2 mS cm^−1^ in the cold-pressed state. Such super Li^+^-conductive GCs ensure stable operation of thick composite cathodes (> 200 μm), realizing ASSBs with high energy density of 420 Wh kg^−1^ by cell mass at room temperature. These relevant results hence open a whole new avenue to pursue superionic conductors.

## Methods

### Synthesis

Reagent-grade Li_2_S (Alfa Aesar, 99.9 %), P_2_S_5_ (MACKLIN, ≥ 99 %), and Al_2_S_3_ (Alfa Aesar, 99.9%) are used as precursors. Given the different coordination polyhedra, e.g., *pyro*-thiophosphate (P_2_S_7_^4−^) or *hypo*-thiophosphate (P_2_S_6_^4−^), are derived from the *ortho*-thiophosphate polyhedra (PS_4_^3−^)^48^, the P/S ratio in various Al_2_S_3_-tuned GCs (Al-GCs) is fixed to 1: 4. To prepare Al-GCs, the reagents based on the stoichiometry listed in Table 1 are weighed out and mixed in zirconia jars followed by mechanical milling for 24 h at a speed of 450 rpm using an apparatus (FRITSCH, Pulverisette7). Afterwards, the obtained powders are pressed into green pellets under 320 MPa. Then the pellets are sealed into an argon-filled quartz tube and heated at 300 °C for 8 h and naturally cooled down to room temperature. The final product powders are obtained by grinding the sintered pellets in a mortar. All synthesis operations are carried out under the protection of argon to prevent the materials from being attacked by moisture and oxygen in the ambient air.

Other experiments can be found in the Supplementary Information.

### Cell assembly

#### steel | steel symmetric cells

Symmetric steel|steel cells are employed to evaluate the ionic and electronic conductivity of the electrolytes. To fabricate steel|sulfide electrolytes|steel symmetric cells, sulfide powders are cold pressed into pellet at 300 MPa inside an 8 mm diameter PEEK die with two stainless-steel rods as current collectors.

#### Li | Li symmetric cells

Symmetric Li|Li cells are employed to evaluate the critical current densities, Li stripping/plating behavior of the electrolytes, and to observe the dendrite growth behavior/interfacial evolution through X-ray computed tomography (CT) or an operando optical microscopy observation device. To fabricate Li|Li symmetric cells for evaluating the critical current densities and Li stripping/plating behaviors, 80 mg sulfide powders are pressed under 300 MPa inside an 8 mm diameter PEEK die to form a solid pellet. Then, two pieces of Li metal with thickness 50 μm and diameter 6 mm are placed onto each side of the sulfide pellet and following pressed by 120 MPa for 5 min to form the symmetrical cell. To assemble the tomography cell, one piece of lithium disc with thickness 100 μm and diameter 2.5 mm is firstly placed on a 3 mm diameter steel pin, which is interposed in a polyimide tube with diameter of 3 mm. Then, sulfide powders, another 2.5 mm diameter lithium disc on a steel pin are placed on top of each other. Afterwards, a pressure of ~110 MPa is applied to them. After releasing the pressure, the obtained symmetrical cell is sandwiched by two steel M5 bolts for testing. For the assembly of operando optical microscopy cell, sulfide powders are pressed under 300 MPa inside a half-round PEEK die to form a half-round solid pellet. Then, two pieces of half-round Li discs are placed onto both sides of the half-round sulfide pellet. Afterwards, a pressure of 120 MPa is applied to the three layers to form the half-round symmetrical cell.

#### Al-GCs/VGCF | Li_6_PS_5_Cl | Li/In batteries

These batteries are assembled for CV testing to evaluate the electrochemical stable windows of Al-GCs. To this end, the cathode composites are firstly prepared by ball-milling Al-GCs and VGCF powders according to a weight ratio of 7:3 at 100 rpm for 2 h using an apparatus (FRITSCH, Pulverisette7). The electrolyte layer is prepared by pressing 80 mg Li_6_PS_5_Cl powders at 200 MPa inside a 10 mm diameter PEEK die. Then, 10 mg of the cathode composites are dispersed on the surface of the as-prepared electrolyte pellet and pressed at 350 MPa. Finally, one piece of Li-In alloy foil is attached on the other side of the electrolyte pellet and pressed at 100 MPa.

#### LiCoO_2_ | Li or LiCoO_2_ | Li/In and NCM811 | Li/In batteries

The LiCoO_2_ | Li or LiCoO_2_ | Li/In and NCM811/Li/In batteries are prepared and tested as described below. The cathode composites are firstly prepared by manual grinding LiNbO_3_ coated LiCoO_2_ or bare-/LiNbO_3_ coated-NCM811 and LPS7228 powders according to a weight ratio of 7:3. The electrolyte layer is prepared by pressing 80 mg electrolyte powders at 200 MPa inside a PEEK die (diameter of 10 mm). Then, 10 mg of the cathode composites are dispersed on the surface of the as-prepared electrolyte pellet and pressed at 350 MPa. Finally, one piece of lithium or indium foil (0.1 mm thickness, 8 mm diameter) is attached on the other side of the electrolyte pellet and pressed at 100 MPa. The LiCoO_2_ or NCM811 loading is 8.92 mg cm^−2^.

#### Thick-cathode battery

For thick-cathode cell, active material LiNbO_3_ coated LiNi_0.6_Mn_0.2_Co_0.2_O_2_ and LPS7228 are uniformly mixed in a mass ratio of 80:20 to prepare the cathode composite. The fabricated LPS7228 film is cut into circular discs with 10 mm diameter. The mass of the used electrolyte film is around 8 mg. Afterwards, the LPS7228 film is placed into a 10 mm diameter PEEK die. Next, ~50 mg cathode composites are placed on one side of the film and μSi anode with diameter of 10 mm and active material of 2.2 mg is attached on the other side. The obtained sandwiched pellet is then pressed under 350 MPa with stainless steel as collectors on both sides.

### Characterization

#### Cells or batteries

An electrochemical working station (Biologic VMP-300) is used to perform the EIS experiments in a frequency range of 7 M to 100 mHz with an applied amplitude of 50 mV. DC polarization measurement is carried out to determine the electronic conductivity. Subsequently, time dependence of the current is measured under constant voltage 0.1 V for 2 h by using the electrochemical working station Biologic VMP-300. For CV testing, to probe the upper potential of the electrochemical stable window, the cell is cycled at a scan rate of 0.1 mV/s, starting at the open-circuit potential, up to 3.5 V and back to 1 V vs. Li^+^/Li and the cycle ends at the open-circuit potential. To probe the lower potential of the electrochemical stable window, the cell is cycled at a scan rate of −0.1 mV/s, starting at the open-circuit potential, down to 0.5 V and back to 2.0 V vs. Li^+^/Li. Operando optical microscopy observation is performed in optical microscope (OLS-4000 OLYMPUS) at home-made device with a transparent window, where Li|Li symmetric cells with different electrolytes are assembled and the process of Li deposition at 0.5 mA cm^−2^ is captured in real time. For the tomography cells, these cells after cycling at 0.5 mA cm^−2^ are measured by laboratory X-ray CT instrument (Rigaku CT lab HX) under an automatic mode. The scan mode is High Resolution and the scan time is 68 min. During the measurement, the X-ray tube voltage is 70 kV and the tube current is 116 μA. The geometry between the sample and the detector is Long and the used field of view (FOV) is 10. The distance between Focus and Object is 32 mm and the distance between Focus and Detector is 432 mm. The resultant spatial resolution is 3.6 μm. The batteries are galvanostatically cycled using a Land battery test system (Land CT2001A, Wuhan Land Electronic Co. Ltd., China).

#### Diffraction

Lab X-ray diffraction (XRD) measurements are carried out using a Rigaku SmartLab X-ray diffractometer with Cu Kα radiation. The diffraction data are collected with a step width of 0.01° over a 2θ range of 10−80°. Synchrotron XRD measurements are performed using the BL01C2 beamline at Taiwan Synchrotron Radiation Research Center, with an X-ray wavelength of 0.82657 Å. The specimens are sealed under an Ar atmosphere in Lindemann glass capillaries (∼0.3 mm inner diameter). Diffraction data are collected in 0.01° steps between 5 and 35° at 298 K. Rietveld refinements for the structural parameters are conducted using the GSAS II program^49^. Initially, the structural information obtained from the Materials Project database. These steps are used during the refinements: (i) scale factor, (ii) 12 coefficients for a Chebyshev function background, (iii) peak shape parameters, (iv) lattice parameters, (v) zero error, (vi) fractional atomic coordinates, (vii) atomic occupancies. Then, multiple correlated parameters are refined simultaneously, and the related constraints Tables can be found separately in Supporting Information.

#### Raman spectra

These are acquired using a micro-Raman spectrometer (Renishaw, inVia) equipped with a 532 nm Ar ion laser.

#### Solid-state NMR

Solid-state NMR experiments are performed on 19.96 T Varian VNMRS spectrometer (^31^P Larmor frequency of 344.09 MHz) using 1.6 mm T3 MAS probe. ^31^P MAS NMR spectra are recorded using 90^o^ pulse of 3 μs length and a recycle delay of 50 s. Prior to NMR experiments, the sample is packed into 1.6 mm Al-reduced zirconia rotor in Ar-filled glovebox. N_2_ gas flow is used for MAS drive and bearing gas. The connectivity between phosphorus units is investigated by 2D ^31^P refocused INADEQUATE experiments. ^31^P chemical shifts are referenced to 85 % H_3_PO_4_ solution. All of the NMR data are processed with ssNake freeware^50^.

#### Cryogenic transmission electron microscope (cryo-TEM)

A TEM cryo-holder (Gatan 626) is used to load the sample where TEM grids are immersed in liquid nitrogen and then mounted onto the holder via a cryo-transfer workstation. The TEM images, high-resolution TEM (HRTEM) images and selected-area electron diffraction (SAED) patterns are obtained using Tecnai G^2^ F20 (FEI) at an acceleration voltage of 200 kV.

#### off-axis Electron holography

Electron hologram image is obtained by using FEI-Themis Z with High brightness Schottky field emission electron source and an accelerating voltage of 80 kV.

#### X-ray photoelectron spectroscopy (XPS)

The samples are placed onto double-sided tape fixed to clean glass slides and placed in a vacuum transfer holder inside an Ar-filled glovebox. An X-ray photoelectron spectroscopy spectrometer (Thermo Scientific Model K-Alpha XPS) with a monochromatized Al Kα source (1486.6 eV) is used to obtain the surface chemistry of the samples. A 400 μm X-ray spot size is used to maximize the signal intensity and to obtain an average surface composition over a large area. The sample surface is cleaned via Ar^+^ sputtering with an acceleration voltage of 0.5 kV for 200 s. A dual beam charge neutralization is applied for charge compensation. The base pressure in the analysis chamber is 3 × 10^−10^ mbar. The pass energy is 23.5 eV. Spectra are charge corrected using the C 1 s core level peak set to 284.8 eV. Data evaluation is performed with the software Thermo Avantage XPS software package (version 5.976).

#### Computation method

We employ the Vienna Ab Initio Package (MedeA-VASP5.4)^51,52^ to perform all the DFT calculations within the GGA using the PBE formulation^53^. We chose the projected augmented wave (PAW) potentials^54,55^ to describe the ionic cores and take valence electrons into account using a plane wave basis set with a kinetic energy cutoff of 520 eV. Partial occupancies of the Kohn−Sham orbitals are allowed using the Gaussian smearing method and a width of 0.05 eV. The electronic energy is considered self-consistent when the energy change is smaller than 10^−5^ eV. A geometry optimization is considered convergent when the force change is smaller than 0.01 eV/Å. At low temperature, the entropic contributions to Gibbs free energy change (∆G_r_) are small, and the volume change of solid material is relatively small which could be ignored, so the ∆G_r_ can be approximated by the internal energy change (∆E_r_), ∆G_r_ ≈ ∆E_r_, which can be obtained from DFT calculations^56^.

According to previous reports, the most common bare surfaces are (100) and (010) for Li_2.82_Al_0.06_PS_4_ and Li_7_P_3_S_11_, respectively^57,58^. To obtain the most stable interface structures, their structure optimizations are performed by fixing the bottom 2 layers and 1 layer for Li_7_P_3_S_11_(010)@Li_2.82_Al_0.06_PS_4_ (100) and Li_7_P_3_S_11_(010)@Li_7_P_3_S_11_(010), respectively, meanwhile allowing other atomic positions to vary. The *k*-point meshes of 2 × 3 × 4 and 4 × 2 × 2 are used for Brillouin zone (BZ) sampling for Li_2.82_Al_0.06_PS_4_ and Li_7_P_3_S_11_, respectively. Four interface structures are constructed in Supplementary Fig. 6a. The binding energy (*E*_bind_) is evaluated the Li_7_P_3_S_11_ nucleation on the different surfaces, which is defined as the energy that different layers of Li_7_P_3_S_11_(010) are formed on the Li_2.82_Al_0.06_PS_4_(100) or Li_7_P_3_S_11_(010) surface. The *E*_bind_ is formulated as:

$${E}_{{{{{{\rm{bind}}}}}}}=\frac{1}{A}\left({{E}}_{{{{{{\rm{interface}}}}}}}-{{E}}_{{{{{{{\rm{Li}}}}}}}_{7}{{{{{{\rm{P}}}}}}}_{3}{{{{{{\rm{S}}}}}}}_{11}}-{{E}}_{{{{{{{\rm{Li}}}}}}}_{2.82}{{{{{{\rm{Al}}}}}}}_{0.06}{{{{{{\rm{PS}}}}}}}_{4}}\right)$$

where *E*_interface_ represents the total energy of interface structure. ${{E}}_{{{{{{{\rm{Li}}}}}}}_{7}{{{{{{\rm{P}}}}}}}_{3}{{{{{{\rm{S}}}}}}}_{11}}$ and ${{E}}_{{{{{{{\rm{Li}}}}}}}_{2.82}{{{{{{\rm{Al}}}}}}}_{0.06}{{{{{{\rm{PS}}}}}}}_{4}}$ represent the energy of Li_7_P_3_S_11_(010) and Li_2.82_Al_0.06_PS_4_(100), respectively. *A* is the surface area which we are interesting.

The grain boundaries structures which contain single-crystal structures on either side are created using the coincident site lattice (CSL) theory^59^. During structural optimizations, the 2 × 2 × 1 Monkhorst-Pack *k*-point grid for Brillouin zone is used for Li_7_P_3_S_11_/Li_7_P_3_S_11_ (010) grain boundary structures. Finally, nudged elastic band (NEB) method has been employed to calculate the Li ions migration along the grain boundary and across the grain regions.

The Li chemical potential is the minus of the Li-vacancy formation energy with respect to Li metal (*μ*_*Li*_ = −*E*_*v*_), which is defined as:

$${E}_{v}(L{i}_{i})=[E(L{i}_{i})+{\mu }_{Li(metal)}]-E$$

where the *E*(*Li*_*i*_) and *E* are the energies of the Li_2.82_Al_0.06_PS_4_ or Li_7_P_3_S_11_ with and without defect Li_i_, respectively; and *μ*_*Li(metal)*_ is the Li chemical potential in Li metal with a body-centered cubic-type structure.

## Supplementary information

Supplementary Information

Peer Review File

## Data Availability

The relevant data generated in this study are provided in the Supplementary Information/Source Data file. Source data are provided with this paper.

## Source data

Source Data
