# Supplementary material for: Self-organized hetero-nanodomains actuating super Li+ conduction in glass ceramics
Source: Nat Commun. 2023 Feb 7;14:669. doi: 10.1038/s41467-023-35982-7 (PMC9905078; doi:10.1038/s41467-023-35982-7)
Supplement: Supplementary file 1 — Supplementary Information [file 41467_2023_35982_MOESM1_ESM.docx]

**Supplementary Information**

**Self-organized hetero-nanodomains actuating super Li^+^ conduction in glass ceramics**

Yantao Wang^1,2^, Hongtao Qu^3^, Bowen Liu^4^, Xiaoju Li^5^, Jiangwei Ju^1🖂^, Jiedong Li^1^, Shu Zhang^1^, Jun Ma^1^, Chao Li^4^, Zhiwei Hu^6^, Chung-Kai Chang^7^, Hwo-Shuenn Sheu^7^, Longfei Cui^1^, Feng Jiang^1^, Ernst R. H. van Eck^3^, Arno P. M. Kentgens^3🖂^, Guanglei Cui^1,2🖂^ & Liquan Chen^8^

^1^ Qingdao Industrial Energy Storage Research Institute, Qingdao Institute of Bioenergy and Bioprocess Technology, Chinese Academy of Sciences, Qingdao 266101, People’s Republic of China

^2^ School of Future Technology, University of Chinese Academy of Sciences, Beijing 100049, People’s Republic of China

^3^ Institute for Molecules and Materials, Radboud University, 6525 AJ Nijmegen, The Netherlands

^4^ School of Materials Science and Engineering, Tianjin University of Technology, Tianjin 300384, People’s Republic of China.

^5^ State Key Laboratory of Microbial Technology, Shandong University, Qingdao 266237, People’s Republic of China

^6^ Max Plank Institute for Chemical Physics of Solids, Nothnitzer Strasse 40, D-01187 Dresden, Germany

^7^ National Synchrotron Radiation Research Center, Hsinchu, Taiwan 30076, Republic of China

^8^ Beijing National Laboratory for Condensed Matter Physics, Institute of Physics, Chinese Academy of Sciences, Beijing 100190, People’s Republic of China.

These authors contributed equally: Yantao Wang, Hongtao Qu, Bowen Liu, Xiaoju Li.

^🖂^Email: cuigl@qibebt.ac.cn (G. Cui); a.kentgens@nmr.ru.nl (A.P.M. Kentgens); jujw@qibebt.ac.cn (J. Ju)

**Supplementary Figures**


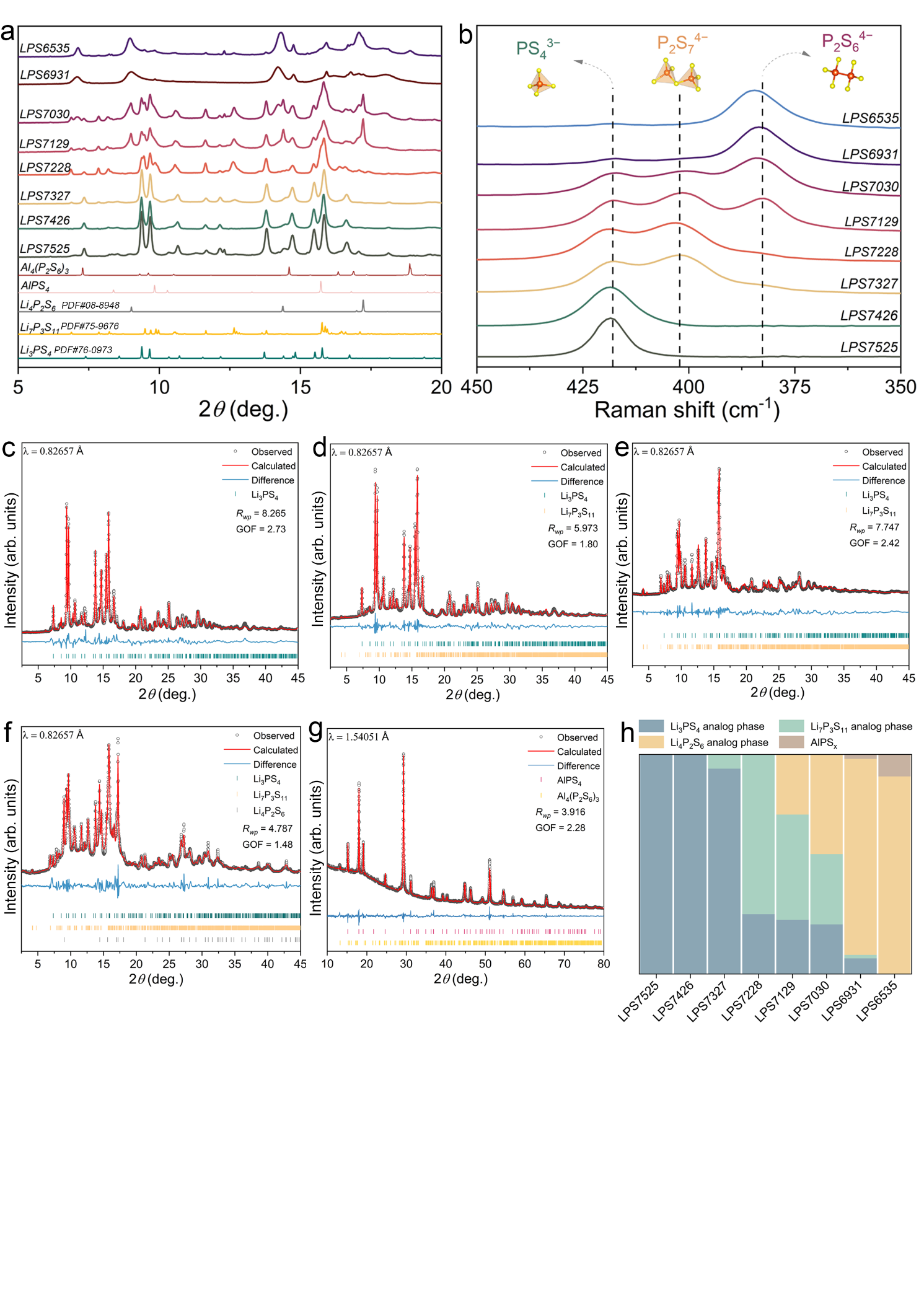


**Supplementary Figure 1**. **Phase composition analysis**. (**a**) SXRD patterns and (**b**) Raman spectra of Al-GCs. Assignments: PS_4_^3−^, 418 cm^−1^; P_2_S_7_^4−^, 402 cm^−1^; P_2_S_6_^4−^, 382 cm^−1^. Rietveld refinements against SXRD for various Al-GCs: (**c**) LPS7525, (**d**) LPS7327, (**e**) LPS7228, and (**f**) LPS7129. (**g**) Rietveld refinement of the XRD pattern of the as-prepared AlPS_4_. 11.2 wt % Al_4_(P_2_S_6_)_3_ side-phase can be distinguished from the AlPS_4_ parent phase. (**h**) Bar graph visualizing the phase evolution of Al-GCs with various Li_2_S : P_2_S_5_ ratios.


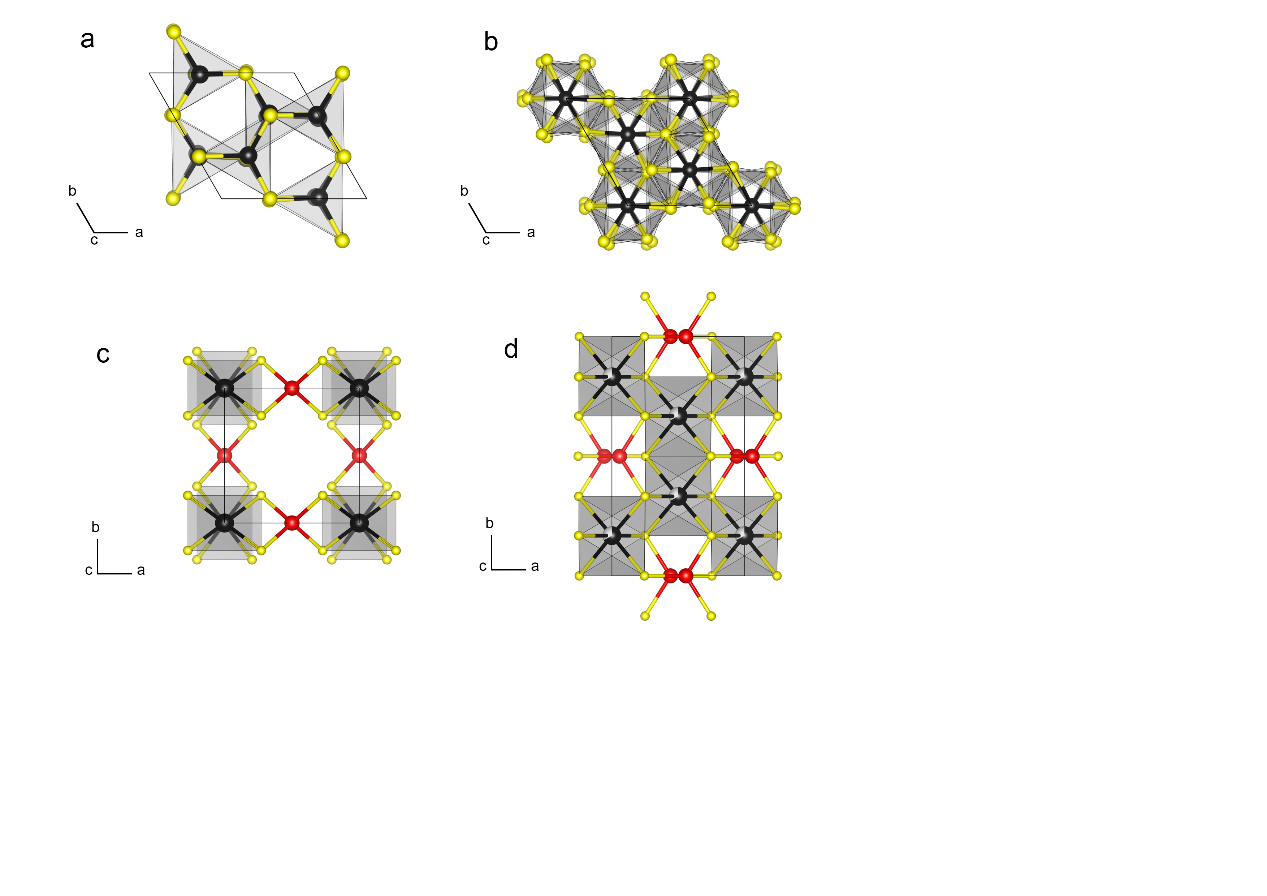


**Supplementary Figure 2.** **Crystal structure.** (**a**) *α*-Al_2_S_3_ built by AlS_4_ tetrahedra and (**b**) *γ*-Al_2_S_3_ built by AlS_6_ octahedra. Al and S atoms are shown in black and yellow, respectively. (**c**) Crystal structure of AlPS_4_ built by AlS_4_ tetrahedra. (**d**) Crystal structure of Al_4_(P_2_S_6_) built by AlS_6_ octahedra. Al, P and S atoms are shown in black, red, and yellow, respectively.


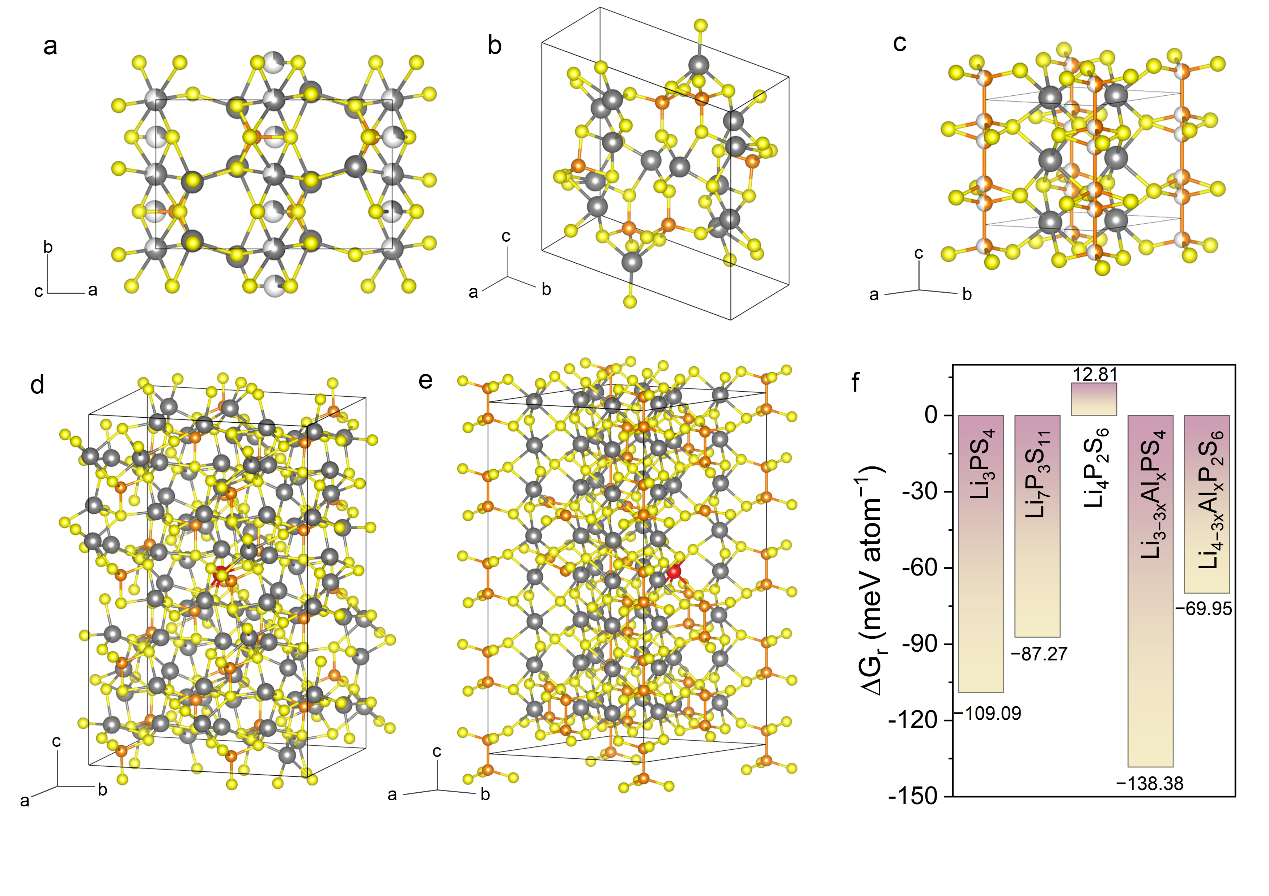


**Supplementary Figure 3. Formation energy.** Crystal structure (**a**) Li_3_PS_4_. (**b**) Li_7_P_3_S_11_. (**c**) Li_4_P_2_S_6_. (**d**) Li_3−3_*_x_*Al*_x_*PS_4_. (**e**) Li_4−3_*_x_*Al*_x_*P_2_S_6_. The Li, P, S and Al atom are depicted with grey, orange, yellow and red spheres, respectively. (**f**) ∆G_r_ of Li_3_PS_4_, Li_7_P_3_S_11_, Li_4_P_2_S_6_, Li_3−3_*_x_*Al*_x_*PS_4_, and Li_4−3_*_x_*Al*_x_*P_2_S_6_.


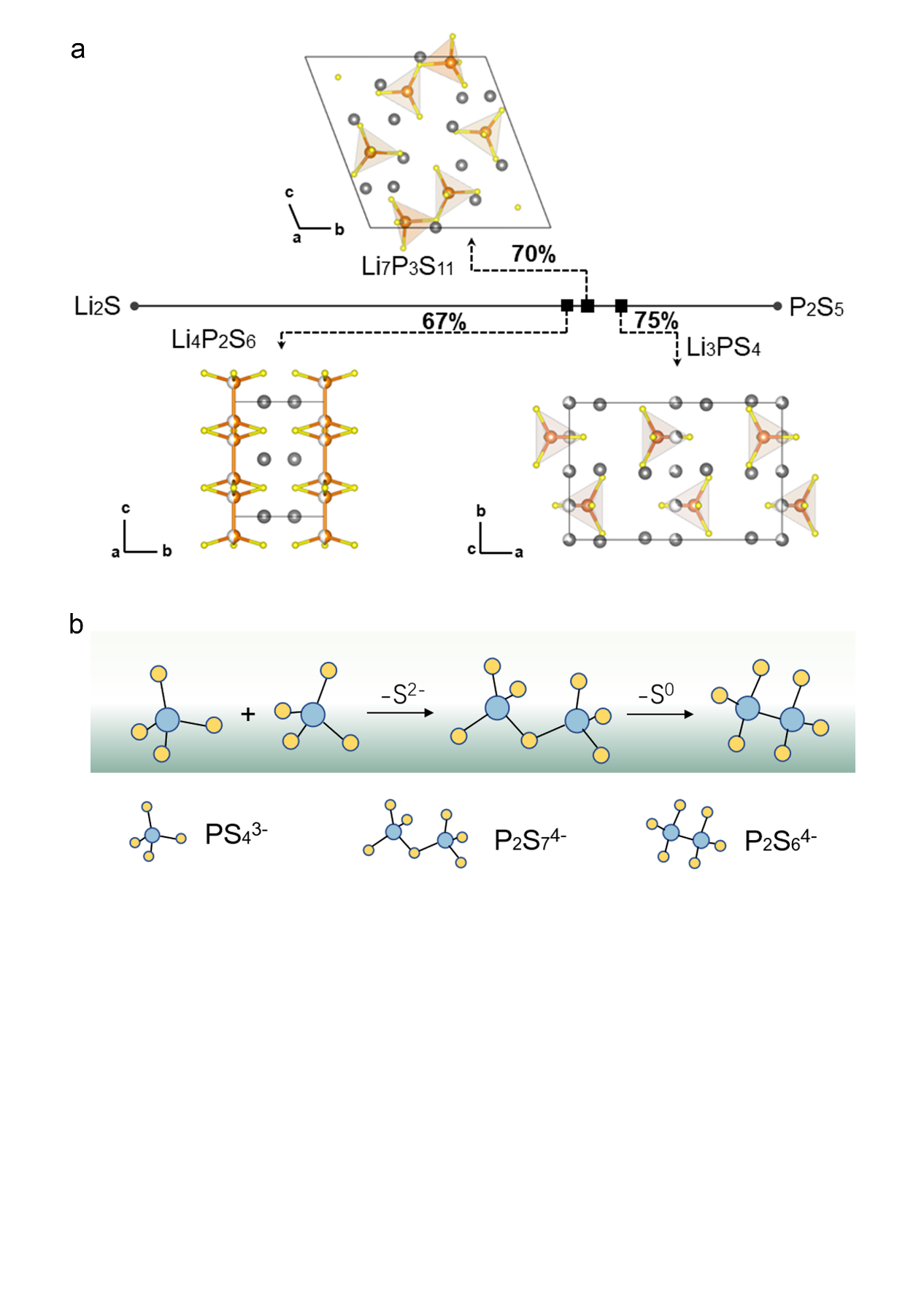


**Supplementary Figure 4. Species evolution.** (**a**) Evolution of the crystal structures with Li_2_S (or P_2_S_5_) fraction in Li_2_S-P_2_S_5_ binary system.^1^ (**b**) Transformation of PS_4_^3−^ to P_2_S_7_^4−^ and P_2_S_6_^4−^.


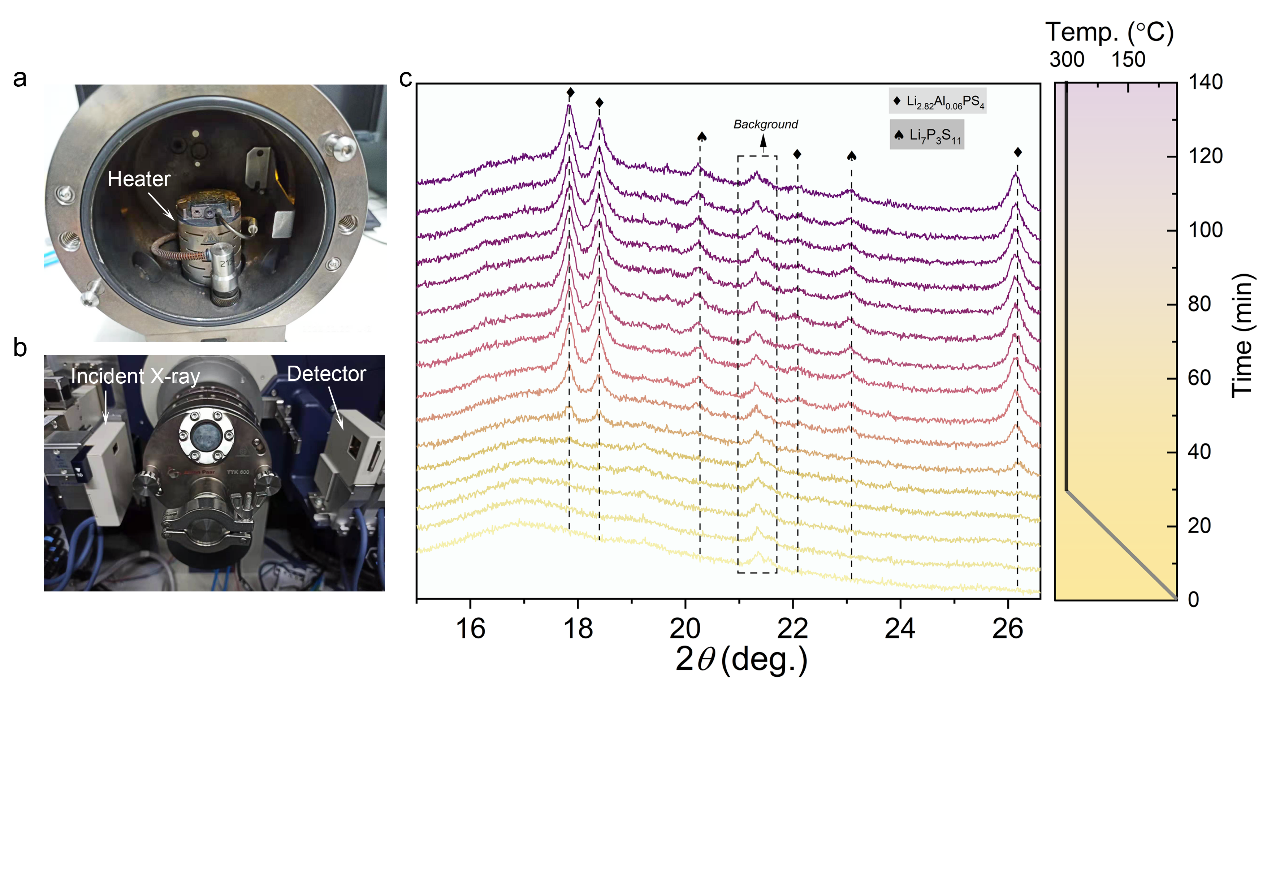


**Supplementary Figure 5. In-situ XRD experiment.** (**a**) The internal structure of ANTON PAAK TTK 600 chamber used in in-situ XRD test. (**b**) Photo of in-situ XRD test. (**c**) in-situ XRD patterns of ball-milled 72 mol% Li_2_S, 28 mol% P_2_S_5_ and 4 mol% Al_2_S_3_ (the reagents of LPS7228) recorded over a temperature range of 30 − 300 °C in argon atmosphere.


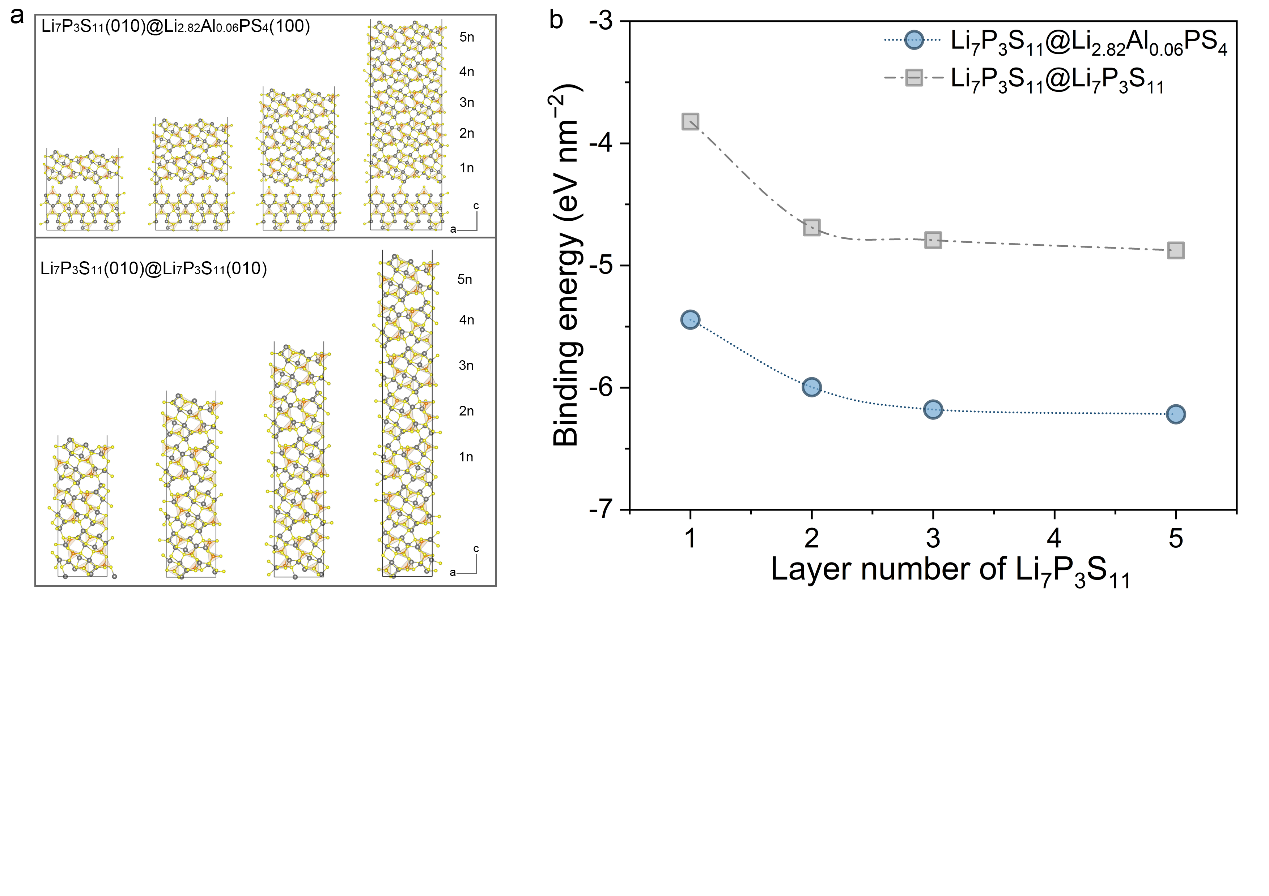


**Supplementary Figure 6. DFT calculations.** (**a**) Four atomic stacking sequences of Li_7_P_3_S_11_(010)@Li_2.82_Al_0.06_PS_4_(100) and Li_7_P_3_S_11_(010)@Li_7_P_3_S_11_(010). (**b**) Binding energy as a function of Li_7_P_3_S_11_ layer number.


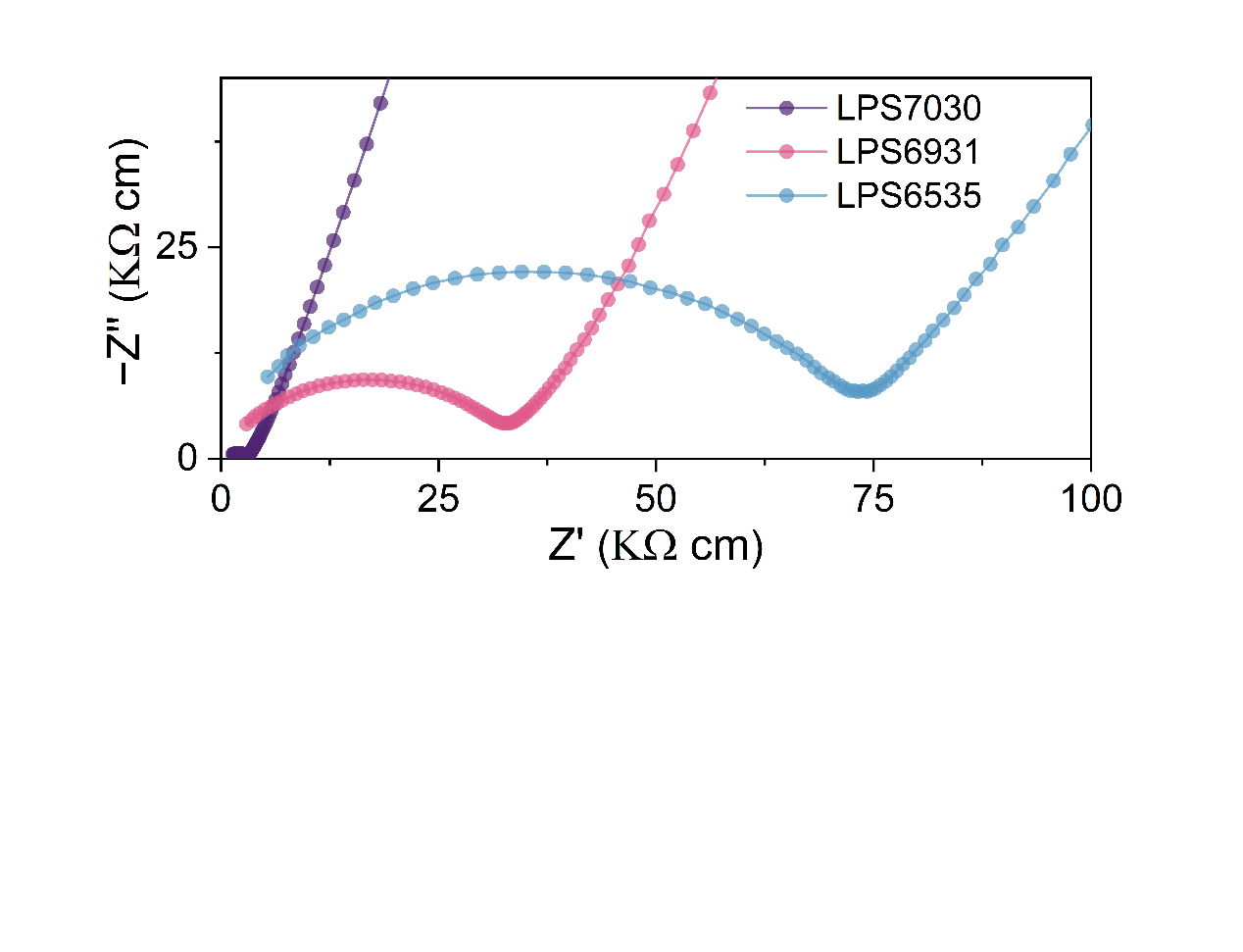


**Supplementary Figure 7. Li^+^-conduction.** EIS plots of LPS7030, LPS6931 and LPS6535 in cold pressed state at room temperature.


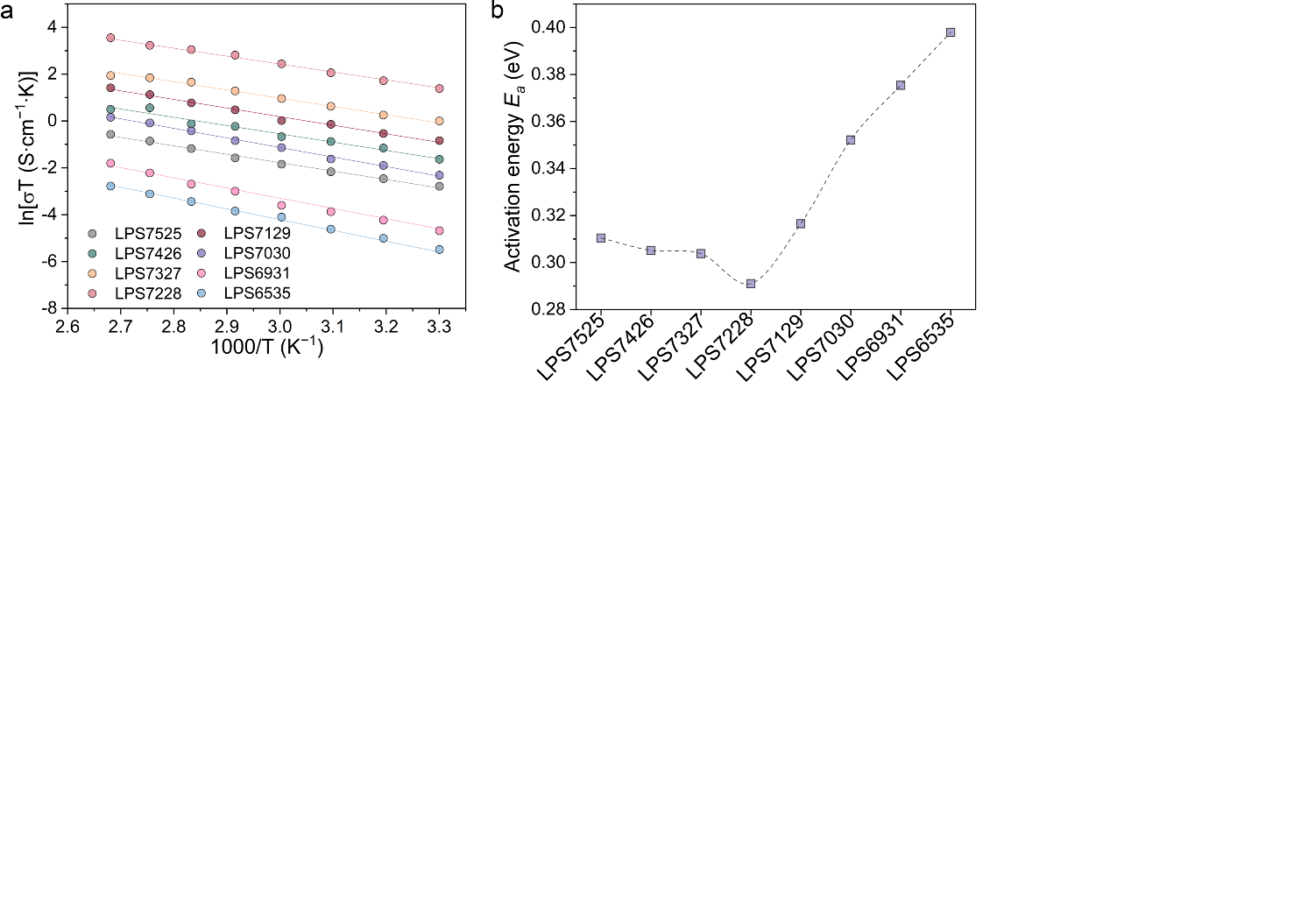


**Supplementary Figure 8. Activation energy.** (**a**) Arrhenius plots of the conductivity for Al-GCs. (**b**) Activation energy for Li^+^ conduction of Al-GCs, as calculated from Arrhenius plots.

*
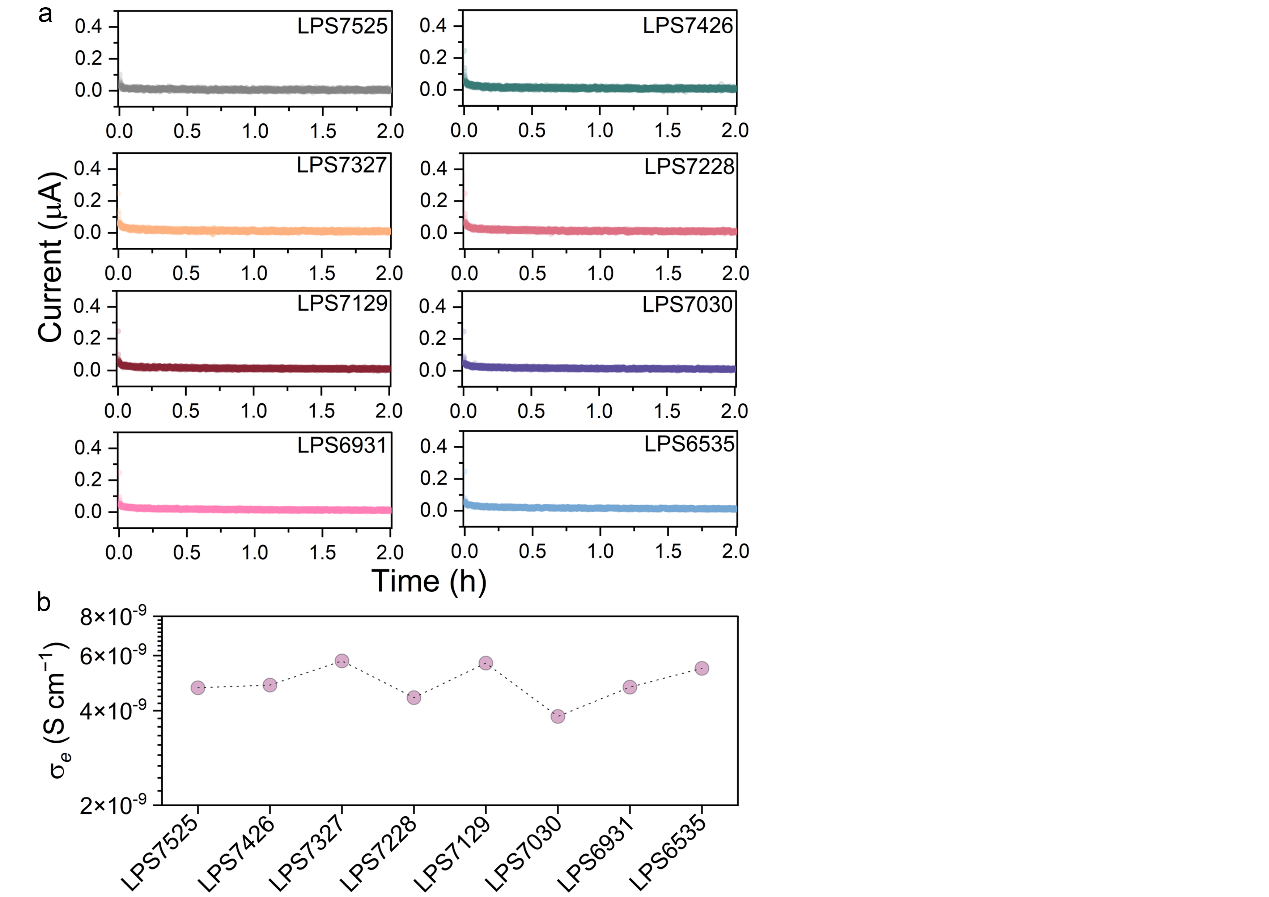
*

**Supplementary Figure 9. Electronic conductivity.** (**a**) DC polarization curves of steel|Al-GCs|steel symmetric cells using a voltage amplitude of 0.1 V. (**b**) The estimated $\sigma_{e}$ of various Al-GCs at room temperature.


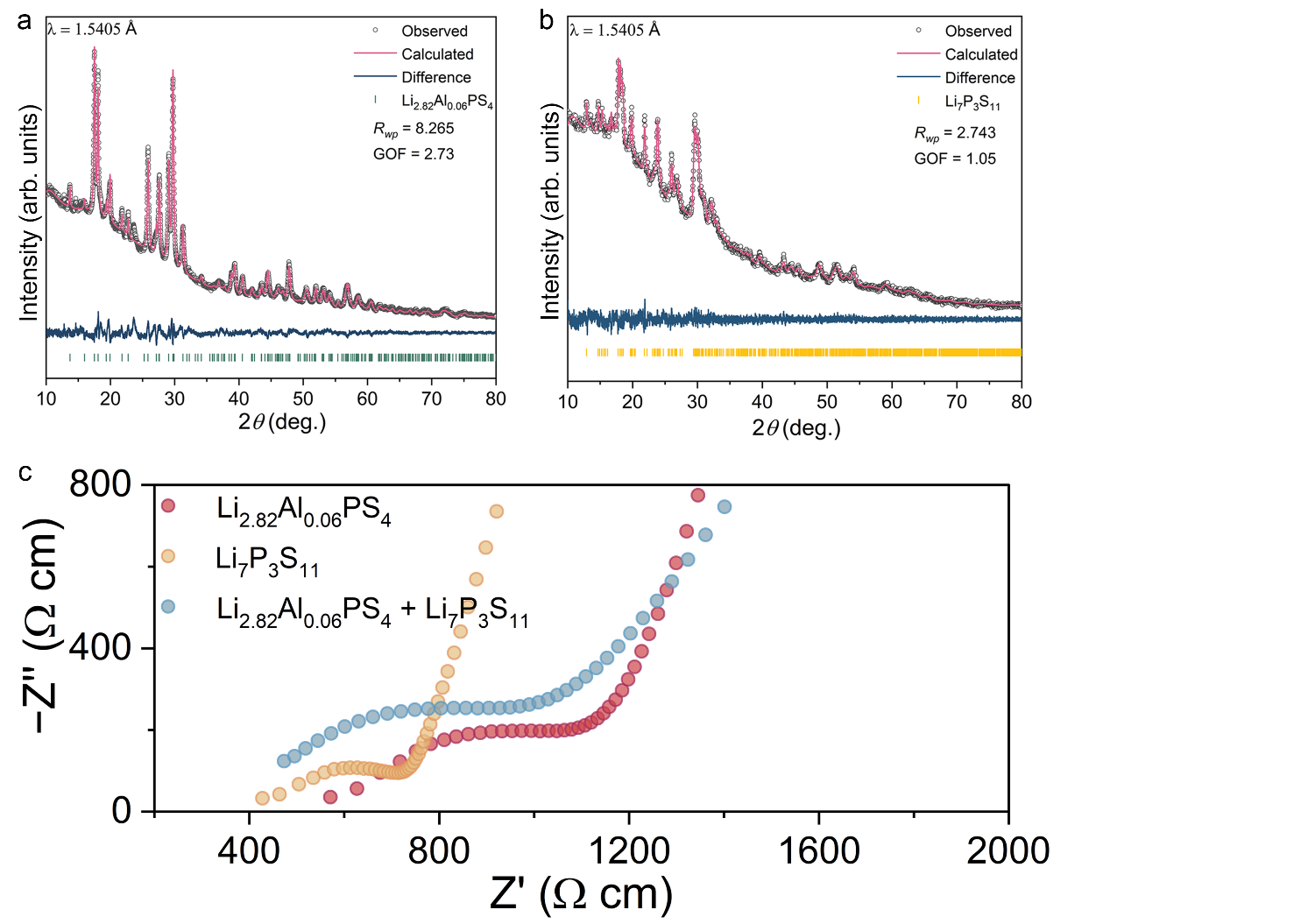


**Supplementary Figure 10.** Rietveld refinement of the XRD patterns of the as-prepared (**a**) Li_2.82_Al_0.06_PS_4_ and (**b**) Li_7_P_3_S_11_. (**c**) EIS plots of Li_2.82_Al_0.06_PS_4_, Li_7_P_3_S_11_, and the factitious Li_2.82_Al_0.06_PS_4_ (27.1 wt%)/Li_7_P_3_S_11_ (72.9 wt%) composite, the same composition as LPS7228.


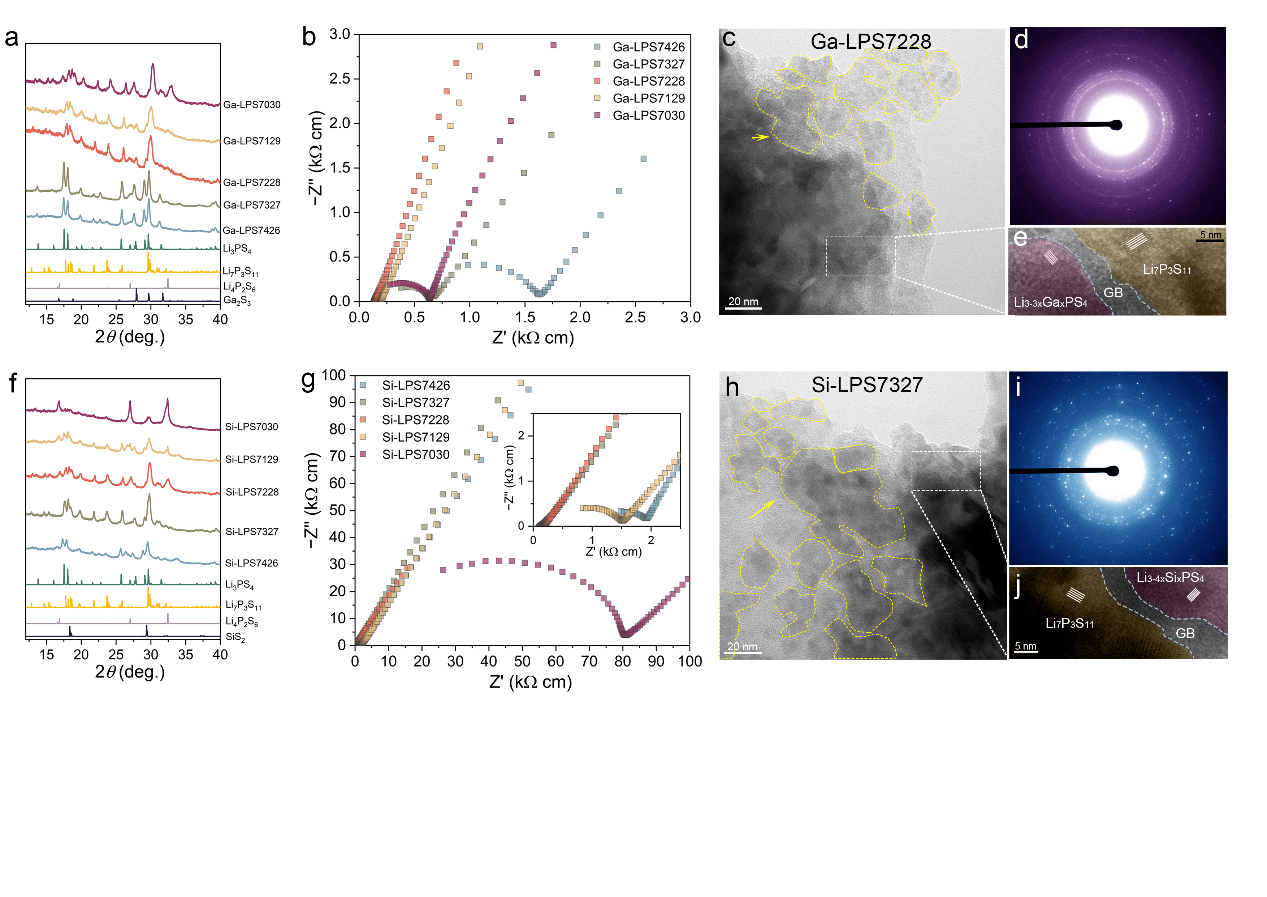


**Supplementary Figure 11. Ga_2_S_3_ and SiS_2_-tuned GCs.** XRD patterns of (**a**) Ga-GCs and (**f**) Si-GCs. EIS plots of (**b**) Ga-GCs and (**g**) Si-GCs at room temperature. Cryo-TEM of (**c**) Ga-LPS7228 and (**h**) Si-LPS7327. SAED of (**d**) Ga-LPS7228 and (**i**) Si-LPS7327. HRTEM of (**e**) Ga-LPS7228 and (**j**) Si-LPS7327.


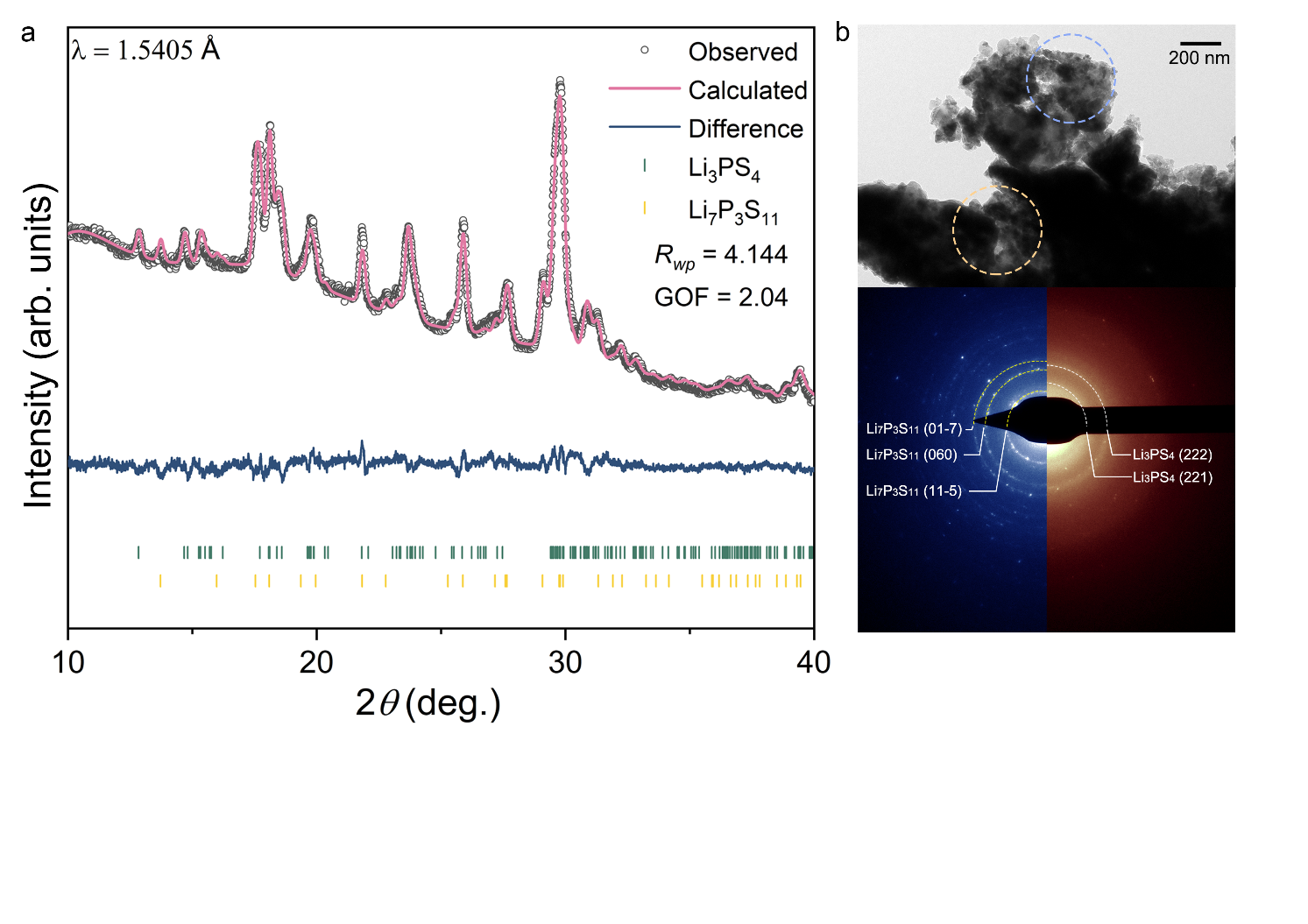


**Supplementary Figure 12. LPS w/o Al_2_S_3_.** (**a**) Rietveld refinement of LPS w/o Al_2_S_3_ XRD pattern. (**b**) cryo-TEM and SAED images LPS w/o Al_2_S_3_.


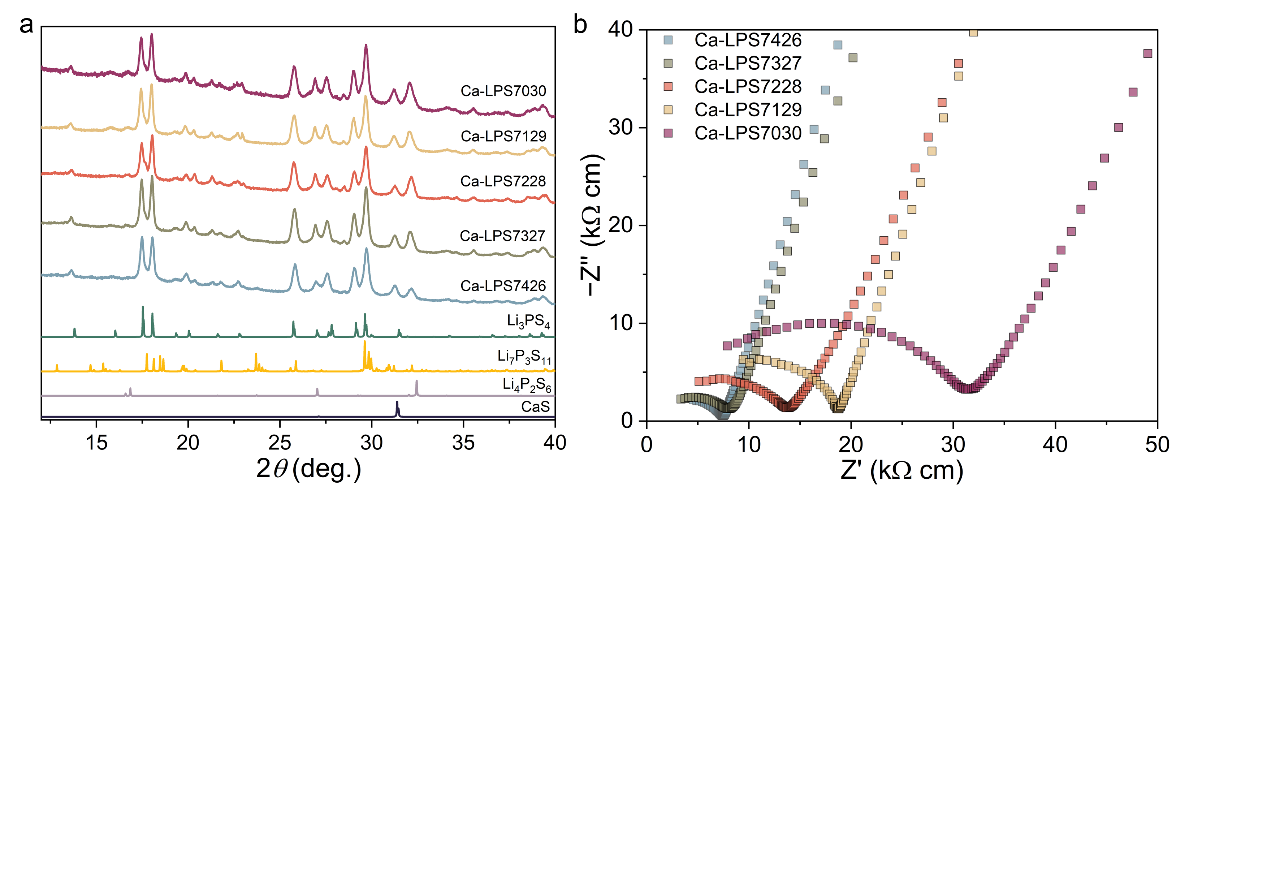


**Supplementary Figure 13. CaS-tuned GCs.** (**a**) XRD patterns of Ca-GCs. (**b**) EIS plots of Ca-GCs at room temperature.

*
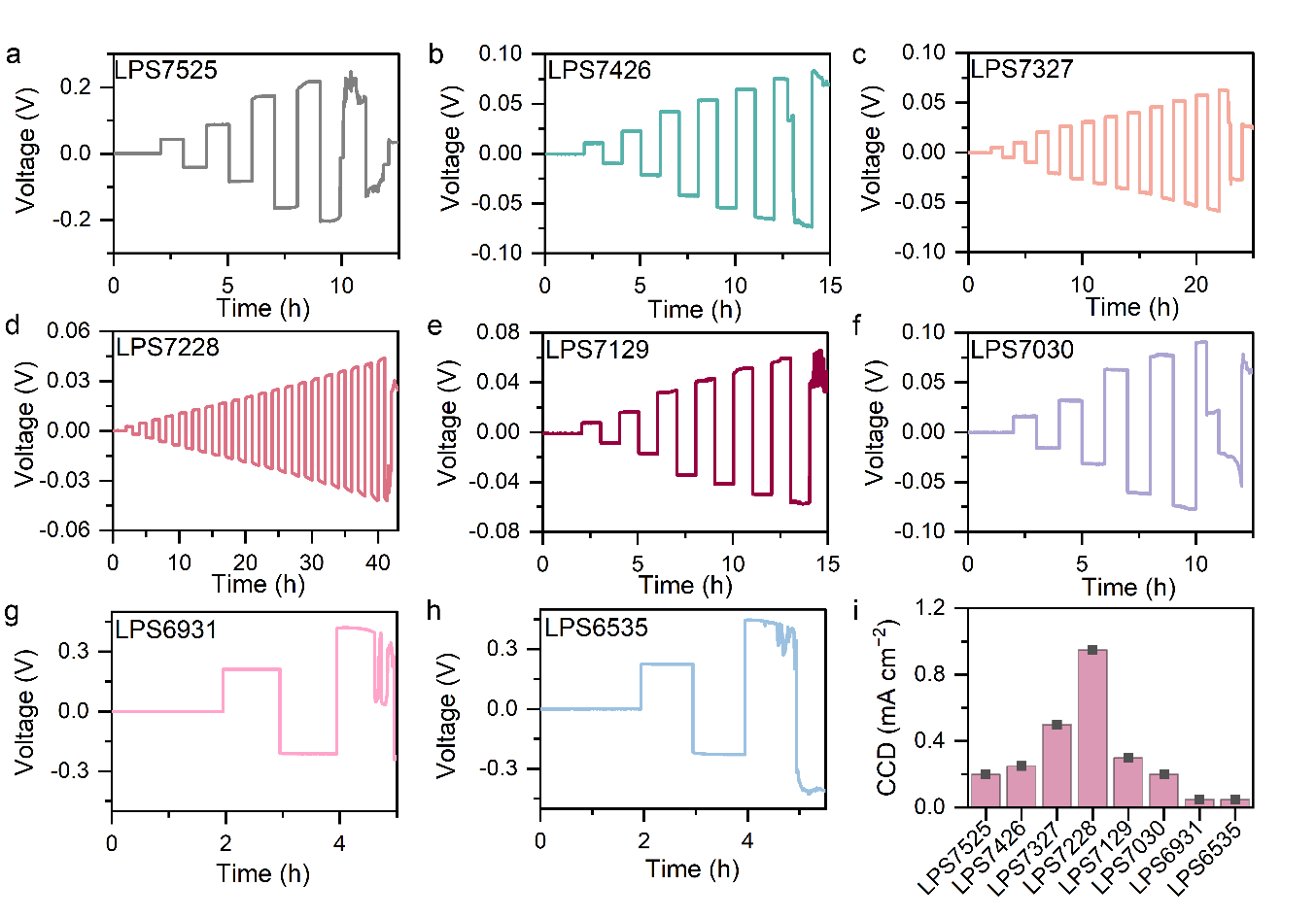
*

**Supplementary Figure 14. Critical current density (CCD).** (**a-h**) Galvanostatic cycling of Li|Al-GCs|Li symmetric cells with step-increased current densities (0.05 mA cm^−2^ for 1 hour) from initial 0.05 mA cm^−2^ at room temperature. (**i**) The CCD of Al-GCs estimated from (**a-h**).


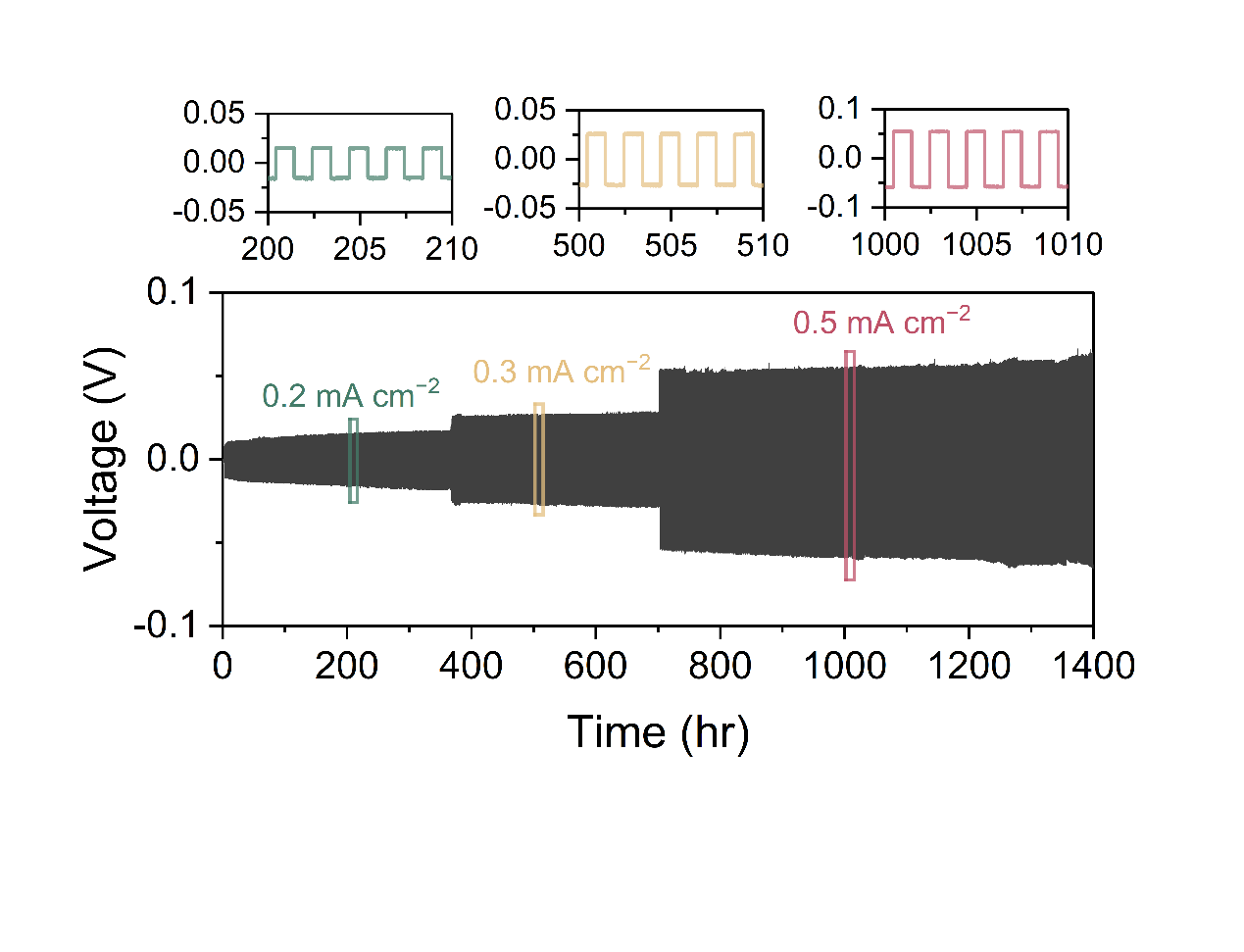


**Supplementary Figure 15.** Galvanostatic cycling measurements of Li|LPS7228|Li at various current densities and room temperature.


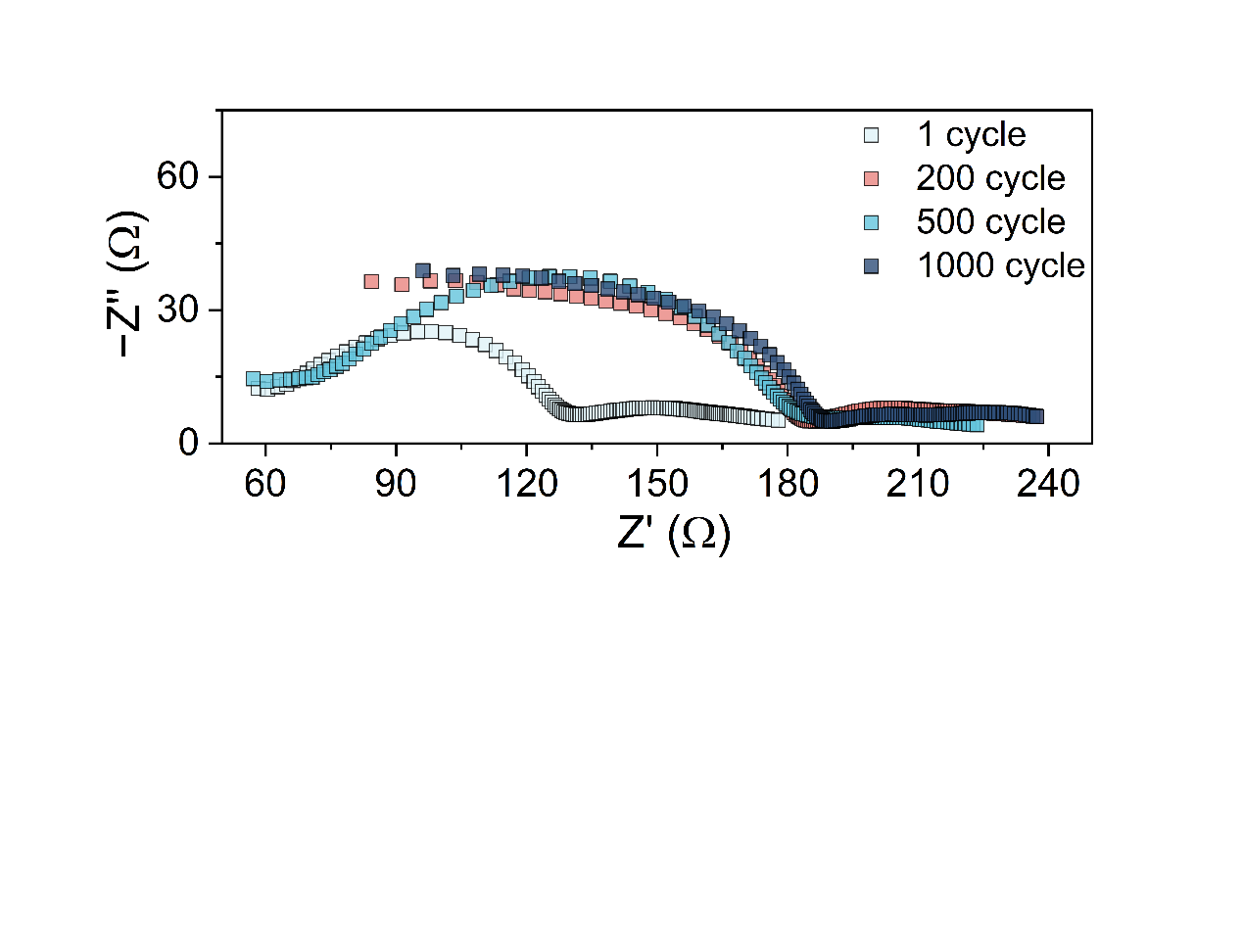


**Supplementary Figure 16**. EIS plots of Li|LPS7228|Li symmetric cell after the 1^st^, 200^th^, 500^th^, 1000^th^ cycles at a current density of 0.2 mA cm^−2^ (0.2 or 1 mAh cm^−2^). Due to the in-situ formed Li|LPS7228 interface, the impedance evolution of Li|LPS7228|Li symmetric cell agrees well with the overpotential evolution as discussed in Fig. 4a.


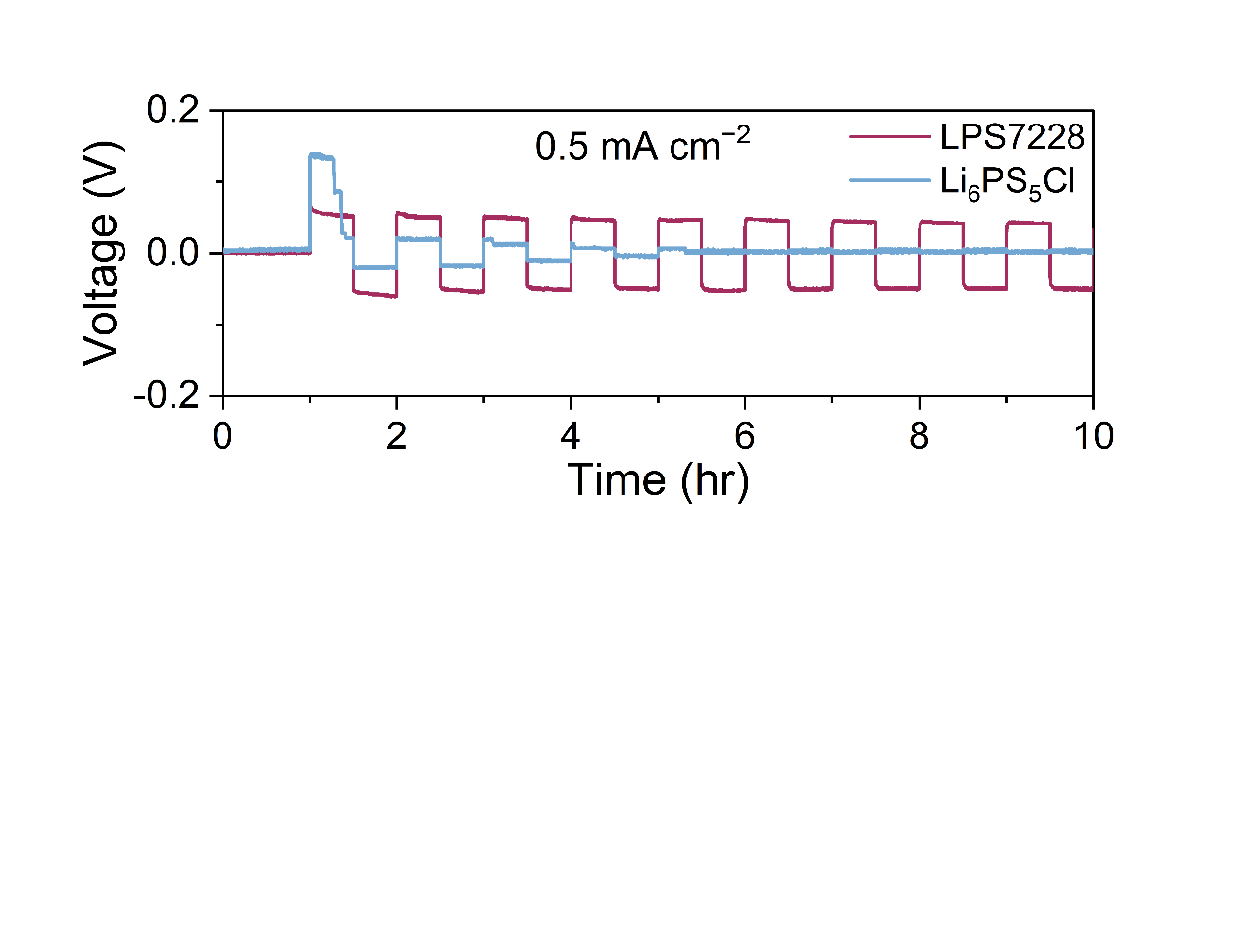


**Supplementary Figure 17**. Galvanostatic cycling of the Li|LPS7228|Li and Li|Li_6_PS_5_Cl|Li symmetric cells at 0.5 mA cm^−2^ and room temperature in our home-designed operando optical microscopy observation device.


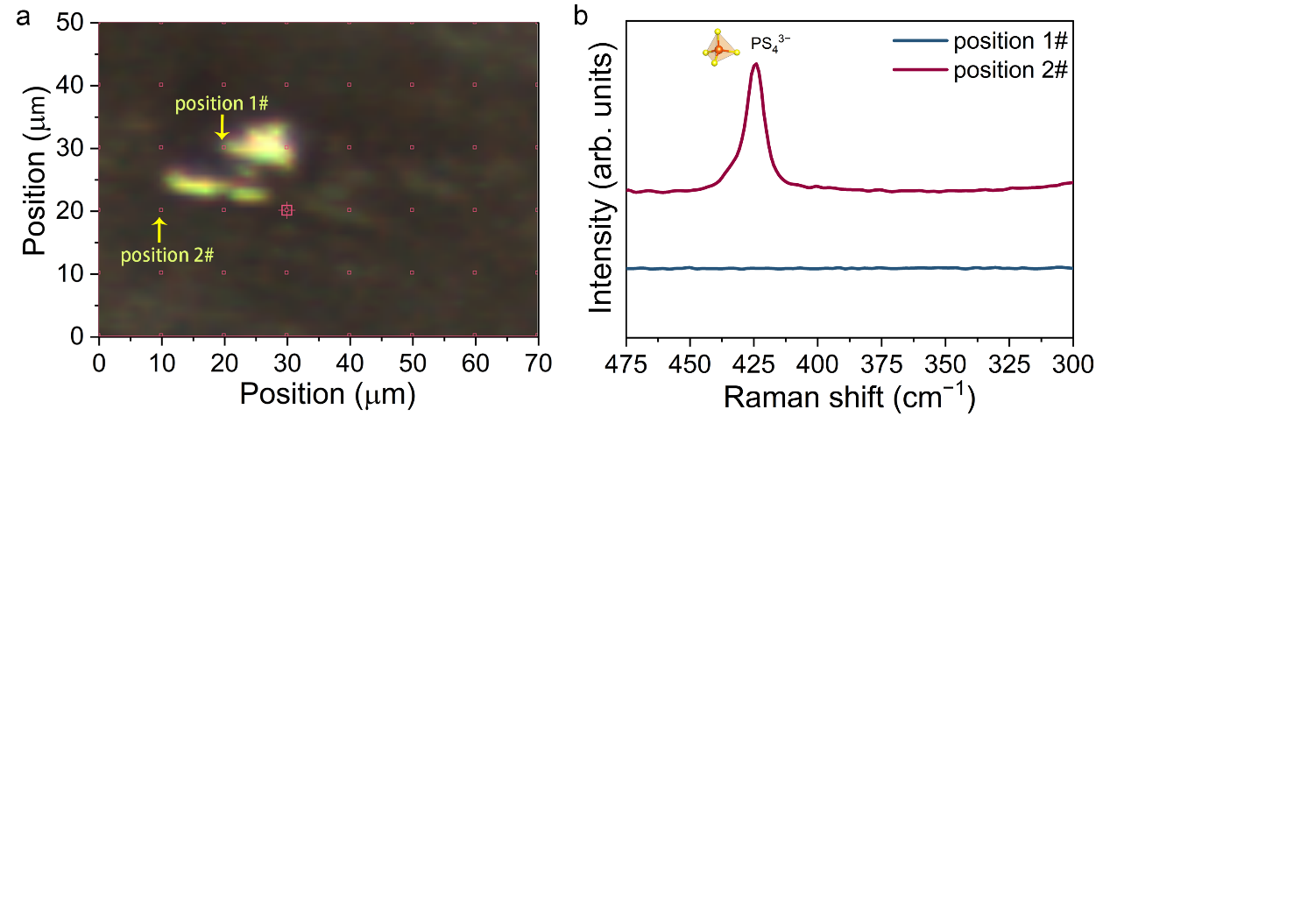


**Supplementary Figure 18. Raman mapping of selected area.** (**a**) Optical image. (**b**) Raman spectra of position 1# and 2# from **a**.


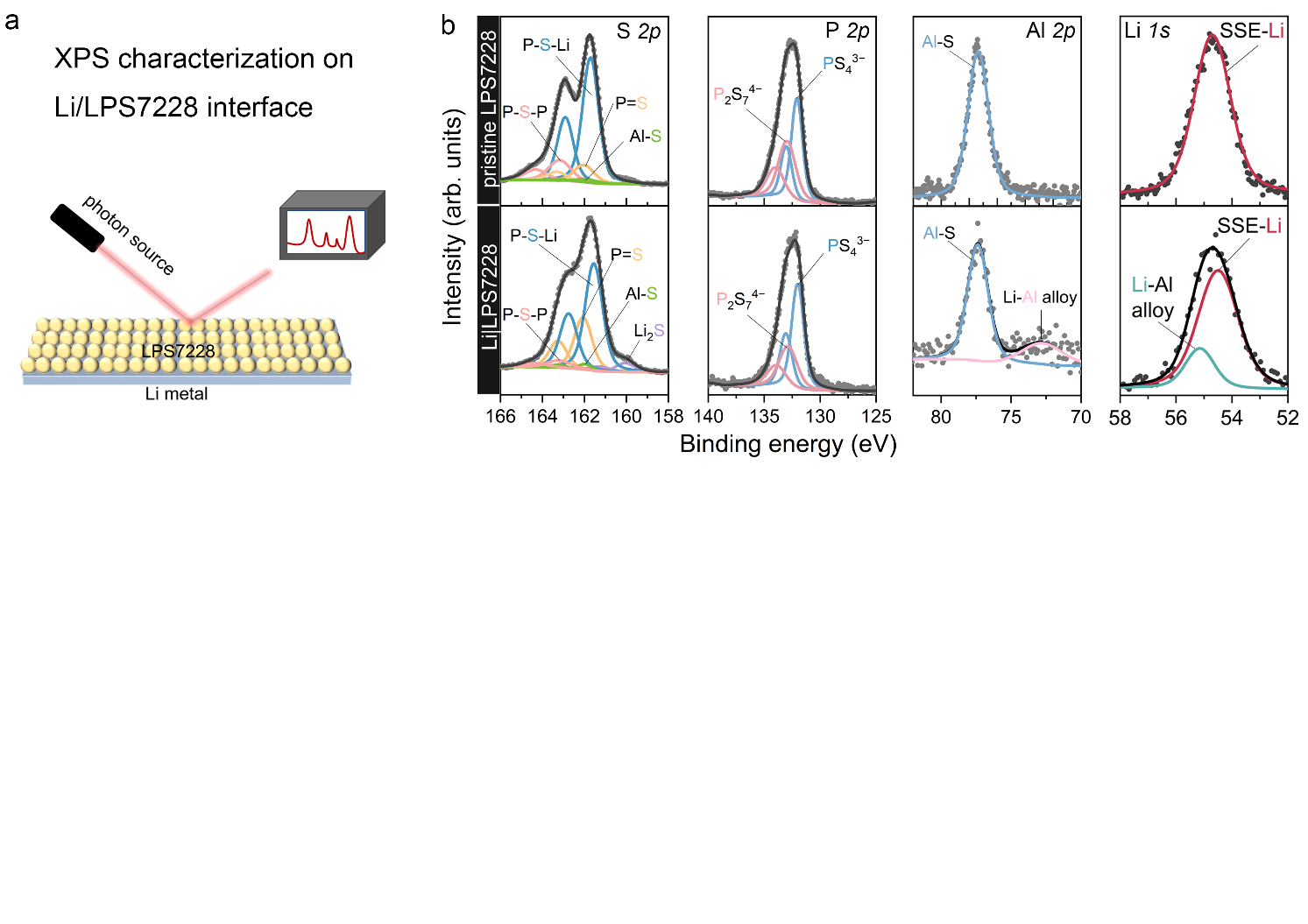


**Supplementary Figure 19. XPS characterization.** (**a**) Schematic illustration of XPS characterization on LPS7228/Li interface. (**b**) S *2p*, P *2p*, Al *2p* and Li *1s* XPS spectra for pristine LPS7228 and the as-cycled LPS7228/Li interface.

**
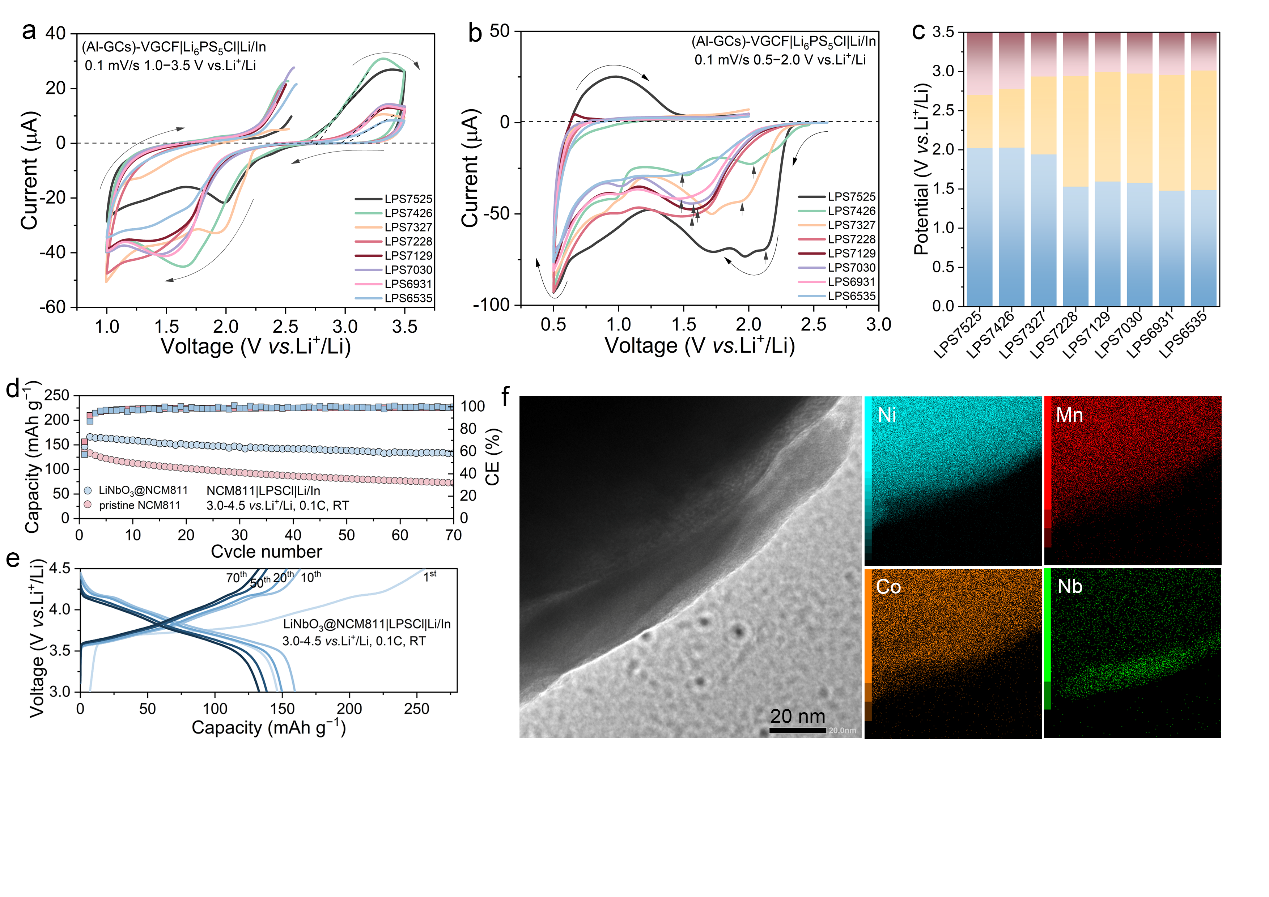
**

**Supplementary Figure 20. Electrochemical stable windows of Al-GCs.** CV of (Al-GCs)-VGCF|Li_6_PS_5_Cl|Li/In cells at a scan rate of 0.1 mV/s in the voltage range of (**a**) 1.0 − 3.5 V and (**b**) 0.5 − 2.0 V. (**c**) Practical EW of Al-GCs estimated from (Al-GCs)-VGCF|Li_6_PS_5_Cl|Li/In cells. The stability region for each electrolyte is shown in orange. (**d**) Cycle behavior of bare-NCM811|Li_6_PS_5_Cl|Li/In and LiNbO_3_ coated NCM811|Li_6_PS_5_Cl|Li/In ASSB at 0.1 C and room temperature. (**e**) Charge-discharge curves of the LiNbO_3_ coated NCM811|Li_6_PS_5_Cl|Li/In ASSB at 0.1 C and room temperature. (**f**) HRTEM image of LiNbO_3_ coated NCM811 particle and Ni, Co, Mn, Nb elemental maps.


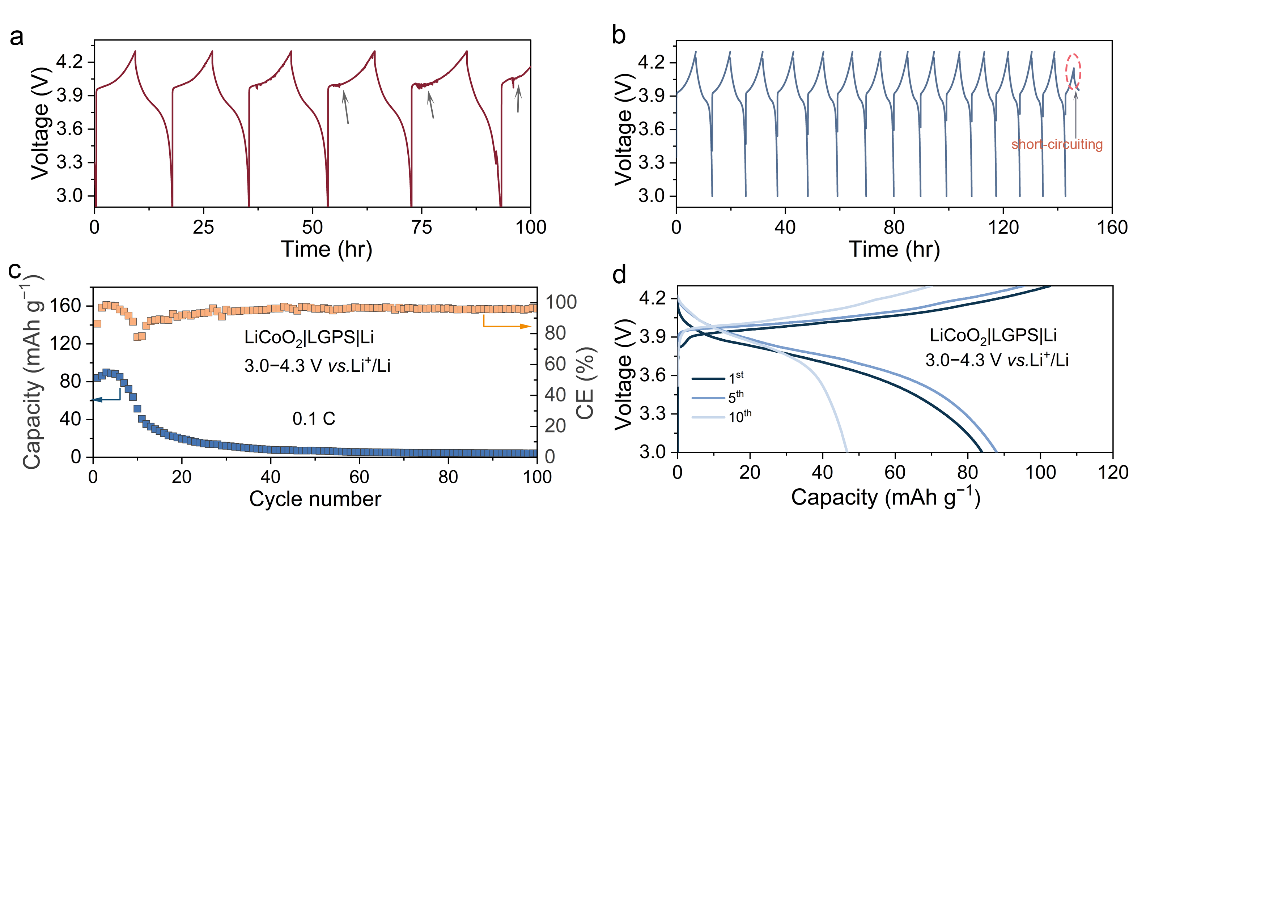


**Supplementary Figure 21.** Electrochemical performance of LiCoO_2_|Li cells at 0.1C (1C = 140 mAh g^−1^) and room temperature. (**a**) Charge and discharge curves of LiCoO_2_|Li_3_PS_4_|Li cell. (**b**) Charge and discharge curves of LiCoO_2_|Li_6_PS_5_Cl|Li cell. (**c**) Cycle behavior of LiCoO_2_|LGPS|Li cell. (**d**) Charge-discharge curves of the LiCoO_2_|LGPS|Li cell.


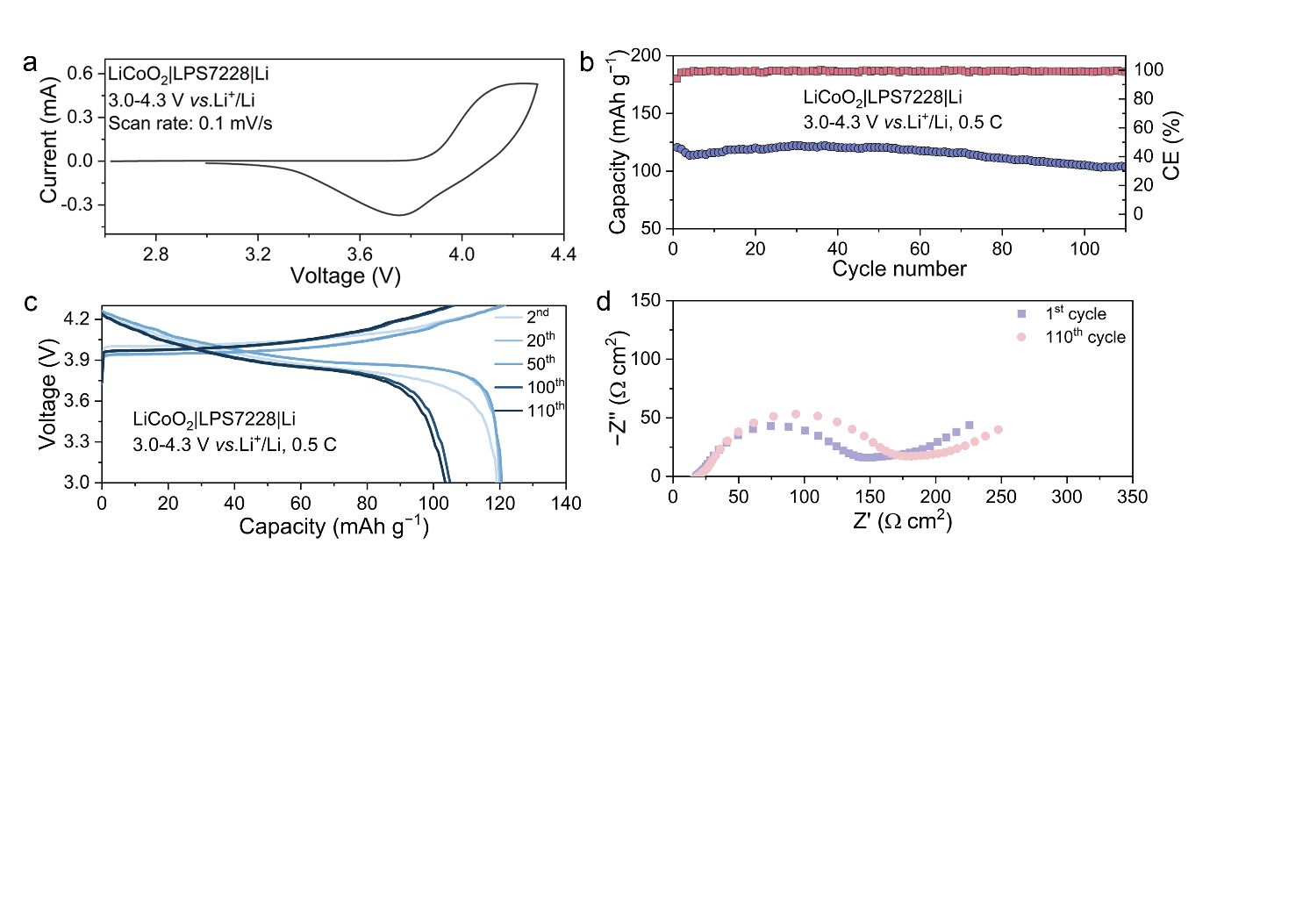


**Supplementary Figure 22**. (**a**) CV profile of LiCoO_2_|LPS7228|Li. (**b**) Cycle behavior and (**c**) charge-discharge curves of LiCoO_2_|LPS7228|Li ASSB at 0.5 C and room temperature. (**d**) EIS plots of LiCoO_2_|LPS7228|Li ASSB after the 1^st^ and 110^th^ discharging.


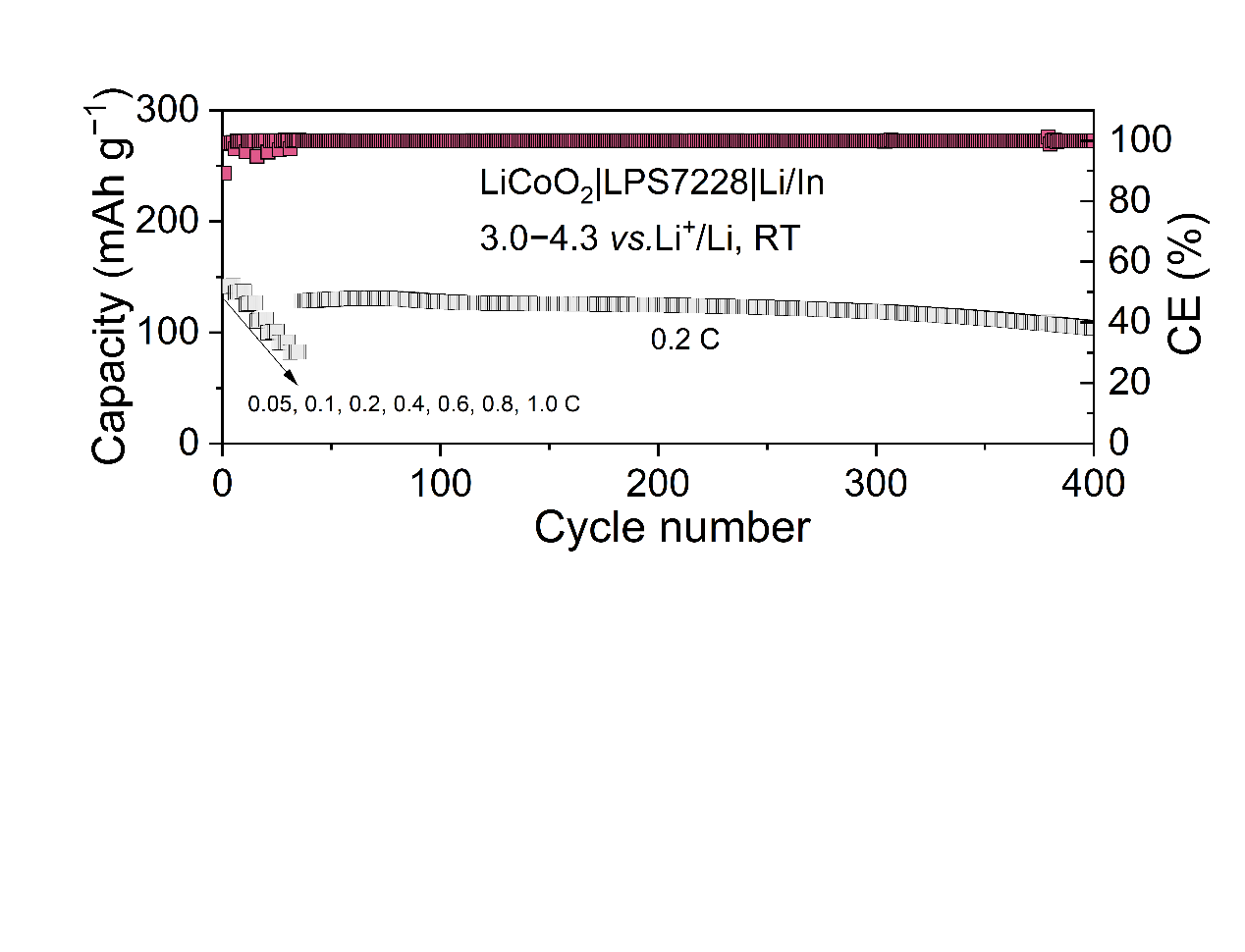


**Supplementary Figure 23**. Cycle behavior of LiCoO_2_|LPS7228|Li/In battery (areal loading, 8.92 mg cm^−2^).

The LiCoO_2_|LPS7228|Li/In battery presents satisfactory discharge capacity of 141 mAh g^−1^ at 0.05 C, 136.7 mAh g^−1^ at 0.1 C, 126 mAh g^−1^ at 0.2 C, and 82.6 mAh g^−1^ at 1 C, respectively. After cycled at 0.2 C for 400 cycles, it still presents a high discharge capacity of 104.2 mAh g^−1^, corresponding to a capacity retention of 82.7 % with an average CE of 99.8 %.


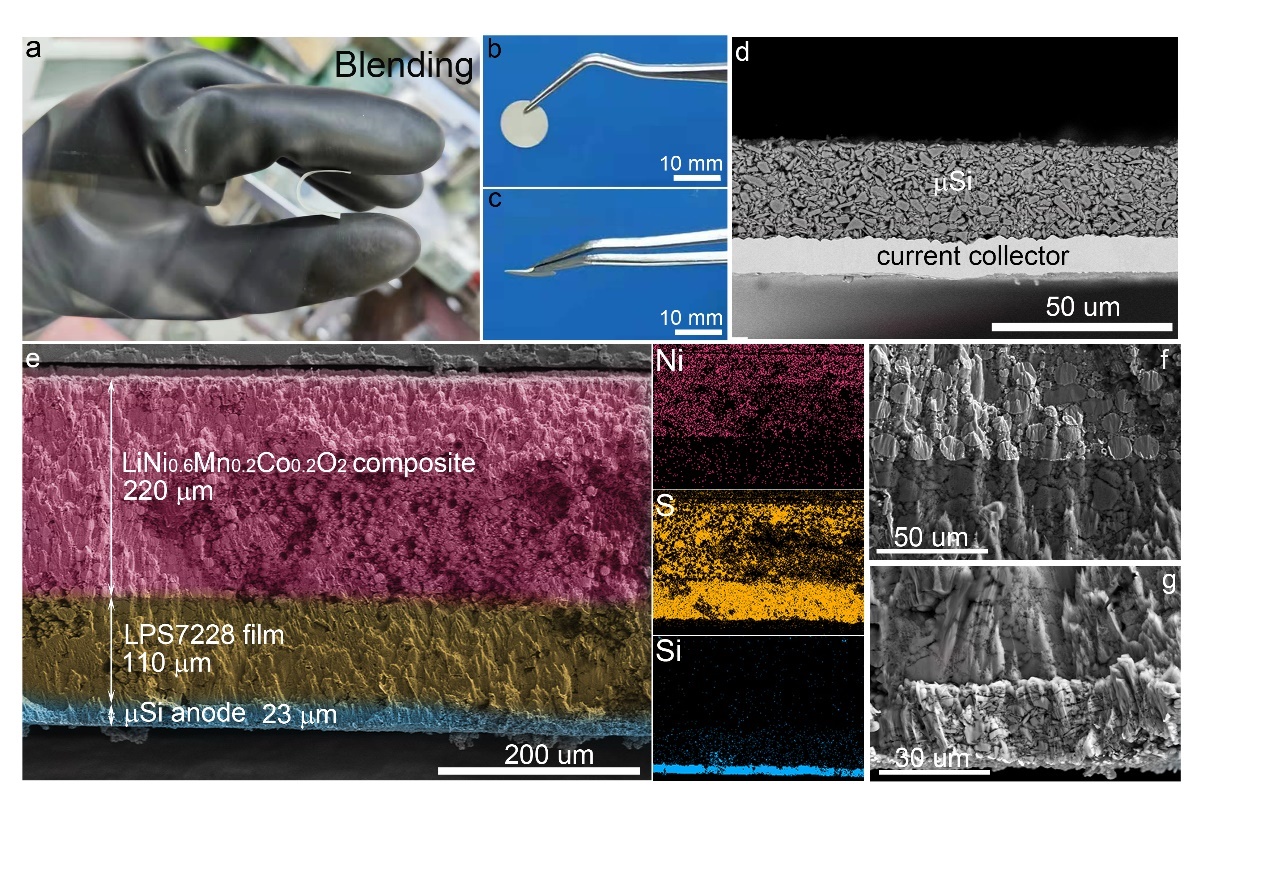


**Supplementary Figure 24. High energy ASSB.** (**a)-(c**) A flexible and free-standing LPS7228 film. (**d**) Microstructure of pristine μSi anode. (**e**) Typical SEM image of cross-sectional and element mapping analysis of the high energy solid-state battery. The cross-sectional morphology of (**f**) cathode/LPS7228 film interface and (**g**) LPS7228 film/anode interface.

**Supplementary Tables**

**Supplementary Table 1**. Stoichiometry (mol%) of Ca-GCs.

| Li_2_S | P_2_S_5_ | CaS | Denotation |
| --- | --- | --- | --- |
| 74 | 26 | 4 | Ca-LPS7426 |
| 73 | 27 | 8 | Ca-LPS7327 |
| 72 | 28 | 12 | Ca-LPS7228 |
| 71 | 29 | 16 | Ca-LPS7129 |
| 70 | 30 | 20 | Ca-LPS7030 |

**Supplementary Table 2**. Stoichiometry (mol%) of Ga-GCs.

| Li_2_S | P_2_S_5_ | Ga_2_S_3_ | Denotation |
| --- | --- | --- | --- |
| 74 | 26 | $\frac{4}{3}$ | Ga-LPS7426 |
| 73 | 27 | $\frac{8}{3}$ | Ga-LPS7327 |
| 72 | 28 | $\frac{12}{3}$ | Ga-LPS7228 |
| 71 | 29 | $\frac{16}{3}$ | Ga-LPS7129 |
| 70 | 30 | $\frac{20}{3}$ | Ga-LPS7030 |

**Supplementary Table 3**. Stoichiometry (mol%) of Si-GCs.

| Li_2_S | P_2_S_5_ | SiS_2_ | Denotation |
| --- | --- | --- | --- |
| 74 | 26 | 2 | Si-LPS7426 |
| 73 | 27 | 4 | Si-LPS7327 |
| 72 | 28 | 6 | Si-LPS7228 |
| 71 | 29 | 8 | Si-LPS7129 |
| 70 | 30 | 10 | Si-LPS7030 |

**Supplementary Table 4**. Crystallographic data of LPS7525. The lattice parameters, fractional atomic coordinates, occupancies are obtained from the Rietveld refinements against the SXRD data.

| Li_3_PS_4_ (space group *P n m a*)  λ = 0.82657 Å lattice parameter a = 12.8846(1) Å, b = 8.1171(4) Å, c = 6.1296(5) Å  *R_wp_* = 8.625 %, Goodness of fit = 2.73 | | | | | |
| --- | --- | --- | --- | --- | --- |
| Atom | Wyckoff position | Atomic coordinates | | | *Occ.* |
|  |  | *x* | *y* | *z* |  |
| S1 | 8d | 0.1513(3) | 0.0470(6) | 0.2791(3) | 1 |
| S2 | 4c | 0.9369(7) | 0.25 | 0.2570(4) | 1 |
| S3 | 4c | 0.0999(4) | 0.25 | 0.8307(1) | 1 |
| P | 4c | 0.0860(6) | 0.25000 | 0.1720(7) | 1 |
| Li1 | 8d | 0.3180(1) | 0.0170(1) | 0.1390(1) | 1 |
| Li2 | 4b | 0 | 0 | 0.5 | 0.7048(8) |
| Li3 | 4c | 0.4420(1) | 0.25 | 0.8307(1) | 0.2951(2) |

**Supplementary Table 5**. Crystallographic data of LPS7426. The lattice parameters, fractional atomic coordinates, occupancies are obtained from the Rietveld refinements against the SXRD data.

| Li_3_PS_4_ (space group *P n m a*)  λ = 0.82657 Å lattice parameter a = 13.0206(5) Å, b = 8.2041(1) Å, c = 6.1894(2) Å  *R_wp_* = 6.298 %, Goodness of fit = 1.90 | | | | | |
| --- | --- | --- | --- | --- | --- |
| Atom | Wyckoff position | Atomic coordinates | | | *Occ.* |
|  |  | *x* | *y* | *z* |  |
| S1 | 8d | 0.1498(2) | 0.0518(4) | 0.2152(3) | 1 |
| S2 | 4c | 0.4402(4) | 0.25 | 0.2585(9) | 1 |
| S3 | 4c | 0.1018(7) | 0.25 | 0.6648(7) | 1 |
| P | 4c | 0.0853(1) | 0.25000 | 0.3254(9) | 1 |
| Li1 | 8d | 0.3213(1) | 0.0245(1) | 0.1177(7) | 1 |
| Li2 | 4b | 0 | 0 | 0.5 | 0.6822(8) |
| Li3 | 4c | 0.4990(1) | 0.25 | 0.8890(1) | 0.3177(2) |

**Supplementary Table 6**. Crystallographic data of LPS7327. The lattice parameters, fractional atomic coordinates, occupancies are obtained from the Rietveld refinements against the SXRD data.

| Li_3_PS_4_ (space group *P n m a*)  λ = 0.82657 Å lattice parameter a = 12.8802(7) Å, b = 8.1437(5) Å, c = 6.1421(2) Å  *R_wp_* = 5.973 %, Goodness of fit = 1.80  Phase fraction : 93.5 wt% | | | | | |
| --- | --- | --- | --- | --- | --- |
| Atom | Wyckoff position | Atomic coordinates | | | *Occ.* |
|  |  | *x* | *y* | *z* |  |
| S1 | 8d | 0.1513(3) | 0.0470(6) | 0.2791(3) | 1 |
| S2 | 4c | 0.9369(7) | 0.25 | 0.2570(4) | 1 |
| S3 | 4c | 0.0999(4) | 0.25 | 0.8307(1) | 1 |
| P | 4c | 0.0860(6) | 0.25000 | 0.1720(7) | 1 |
| Li1 | 8d | 0.3180(1) | 0.0170(1) | 0.1390(1) | 1 |
| Li2 | 4b | 0 | 0 | 0.5 | 0.7048(8) |
| Li3 | 4c | 0.4420(1) | 0.25 | 0.8307(1) | 0.2951 (2) |

| Li_7_P_3_S_11_ (space group *P −1*)  λ = 0.82657 Å lattice parameter a = 6.1141(9) Å, b = 12.4635(4) Å, c = 12.6377(6) Å  *R_wp_* = 5.973 %, Goodness of fit = 1.80  Phase fraction : 6.5 wt% | | | | | |
| --- | --- | --- | --- | --- | --- |
| Atom | Wyckoff position | Atomic coordinates | | | *Occ.* |
|  |  | *x* | *y* | *z* |  |
| S1 | 2i | 0.1019(7) | 0.8669(3) | 0.1146(1) | 1 |
| S2 | 2i | 0.0920(5) | 0.1834(9) | 0.1869(1) | 1 |
| S3 | 2i | 0.4623(7) | 0.1396(8) | -0.0018(8) | 1 |
| S4 | 2i | 0.2118(2) | 0.3542(5) | 0.0035(8) | 1 |
| S5 | 2i | 0.4126(9) | 0.6726(1) | 0.2067(5) | 1 |
| S6 | 2i | 0.3181(1) | 0.4408(8) | 0.3177(6) | 1 |
| S7 | 2i | 0.7734(2) | 0.4674(8) | 0.1983(3) | 1 |
| S8 | 2i | 0.1767(5) | 0.8045(8) | 0.3799(2) | 1 |
| S9 | 2i | 0.2804(6) | 0.1732(1) | 0.4741(9) | 1 |
| S10 | 2i | 0.1680(5) | 0.6575(6) | 0.5529(8) | 1 |
| S11 | 2i | 0.6877(1) | 0.0058(6) | 0.3092(3) | 1 |
| P1 | 2i | 0.1431(5) | 0.1858(7) | 0.0025(5) | 1 |
| P2 | 2i | 0.4724(1) | 0.4584(8) | 0.2200(4) | 1 |
| P3 | 2i | 0.8825(1) | 0.1465(7) | 0.4254(6) | 1 |
| Li1 | 2i | −0.2043(1) | 0.2175(4) | 0.4067(6) | 1 |
| Li2 | 2i | −0.1407(2) | 0.4778(2) | 0.2925(9) | 1 |
| Li3 | 2i | 0.0049(8) | 0.4307(5) | 0.4836(7) | 1 |
| Li4 | 2i | 0.2868(3) | −0.2560(8) | 0.0609(1) | 1 |
| Li5 | 2i | 0.2439(6) | 0.6322(3) | 0.2960(8) | 1 |
| Li6 | 2i | 0.4387(9) | 0.3481(1) | 0.2757(1) | 1 |
| Li7 | 2i | 0.1270(2) | −0.0107(5) | 0.7409(8) | 1 |

**Supplementary Table 7**. Crystallographic data of LPS7228. The lattice parameters, fractional atomic coordinates, occupancies are obtained from the Rietveld refinements against the SXRD data.

| Li_3_PS_4_ (space group *P n m a*)  λ = 0.82657 Å lattice parameter a = 12.8802(7) Å, b = 8.1437(5) Å, c = 6.1421(2) Å  *R_wp_* = 7.747 %, Goodness of fit = 2.42  Phase fraction : 27.2 wt% | | | | | |
| --- | --- | --- | --- | --- | --- |
| Atom | Wyckoff position | Atomic coordinates | | | *Occ.* |
|  |  | *x* | *y* | *z* |  |
| S1 | 8d | 0.1540(2) | 0.0471(3) | 0.2158(9) | 1 |
| S2 | 4c | 0.4351(9) | 0.25 | 0.2644(6) | 1 |
| S3 | 4c | 0.0929(9) | 0.25 | 0.6682(1) | 1 |
| P | 4c | 0.0879(3) | 0.25 | 0.3109(9) | 1 |
| Li1 | 8d | 0.3464(1) | −0.0701(8) | 0.0664(4) | 1 |
| Li2 | 4b | 0 | 0 | 0 | 0.6930(8) |
| Li3 | 4c | 0.7980(5) | 0.25 | 0.8182(7) | 0.3069(2) |

| Li_7_P_3_S_11_ (space group *P −1*)  λ = 1.54051 Å lattice parameter a = 6.0737(2) Å, b = 12.3049(1) Å, c = 12.5403(9) Å  *R_wp_* = 7.747 %, Goodness of fit = 2.42  Phase fraction : 72.8 wt% | | | | | |
| --- | --- | --- | --- | --- | --- |
| Atom | Wyckoff position | Atomic coordinates | | | *Occ.* |
|  |  | *x* | *y* | *z* |  |
| S1 | 2i | 0.0866(5) | 0.8444(6) | 0.1158(4) | 1 |
| S2 | 2i | 0.1220(1) | 0.1599(6) | 0.1712(1) | 1 |
| S3 | 2i | 0.4250(1) | 0.1329(1) | 0.0358(3) | 1 |
| S4 | 2i | 0.2198(3) | 0.3824(9) | 0.0261(1) | 1 |
| S5 | 2i | 0.4354(9) | 0.6775(8) | 0.2002(5) | 1 |
| S6 | 2i | 0.3228(1) | 0.4659(8) | 0.3273(9) | 1 |
| S7 | 2i | 0.8264(6) | 0.4753(7) | 0.1904(1) | 1 |
| S8 | 2i | 0.1478(1) | 0.8176(1) | 0.3938(6) | 1 |
| S9 | 2i | 0.2842(8) | 0.1682(6) | 0.4641(6) | 1 |
| S10 | 2i | 0.1490(4) | 0.6701(1) | 0.5479(7) | 1 |
| S11 | 2i | 0.6966(4) | 0.0249(3) | 0.3115(6) | 1 |
| P1 | 2i | 0.1207(1) | 0.2193(8) | 0.0406(8) | 1 |
| P2 | 2i | 0.5231(4) | 0.5124(3) | 0.2442(9) | 1 |
| P3 | 2i | 0.8583(2) | 0.1252(1) | 0.4184(7) | 1 |
| Li1 | 2i | 0.1011(3) | 0.2867(2) | 0.4506(3) | 1 |
| Li2 | 2i | −0.0237(5) | 0.5789(9) | 0.2884(3) | 1 |
| Li3 | 2i | 0.7264(8) | 0.1511(1) | 0.1038(3) | 1 |
| Li4 | 2i | 0.2938(5) | −0.0267(7) | 0.2780(4) | 1 |
| Li5 | 2i | 0.2701(4) | 0.5245(8) | 0.1077(1) | 1 |
| Li6 | 2i | 0.4328(4) | 0.3646(2) | −0.0420(3) | 1 |
| Li7 | 2i | 0.2332(3) | 0.0389(2) | 0.3858(2) | 1 |

**Supplementary Table 8**. Crystallographic data of LPS7129. The lattice parameters, fractional atomic coordinates, occupancies are obtained from the Rietveld refinements against the SXRD data.

| Li_3_PS_4_ (space group *P n m a*)  λ = 0.82657 Å lattice parameter a = 12.8644(1) Å, b = 8.1253(5) Å, c = 6.1223(1) Å  *R_wp_* = 4.787 %, Goodness of fit = 1.48  Phase fraction : 24.7 wt% | | | | | |
| --- | --- | --- | --- | --- | --- |
| Atom | Wyckoff position | Atomic coordinates | | | *Occ.* |
|  |  | *x* | *y* | *z* |  |
| S1 | 8d | 0.1551(1) | 0.0458(3) | 0.2148(2) | 1 |
| S2 | 4c | 0.4346(7) | 0.25 | 0.2574(3) | 1 |
| S3 | 4c | 0.1068(1) | 0.25 | 0.6634(9) | 1 |
| P | 4c | 0.0872(4) | 0.25 | 0.3281(7) | 1 |
| Li1 | 8d | 0.3562(2) | 0.0130(4) | 0.0610(5) | 1 |
| Li2 | 4b | 0 | 0 | 0.5 | 0.6827(4) |
| Li3 | 4c | 0.4990(2) | 0.25 | 0.8890(3) | 0.3175(6) |

| Li_7_P_3_S_11_ (space group *P −1*)  λ = 0.82657 Å lattice parameter a = 6.0737(2) Å, b = 12.3049(1) Å, c = 12.5403(9) Å  *R_wp_* = 4.787 %, Goodness of fit = 1.48  Phase fraction : 47.9 wt% | | | | | |
| --- | --- | --- | --- | --- | --- |
| Atom | Wyckoff position | Atomic coordinates | | | *Occ.* |
|  |  | *x* | *y* | *z* |  |
| S1 | 2i | 0.0969(3) | 0.8606(8) | 0.1195(3) | 1 |
| S2 | 2i | 0.0713(8) | 0.1765(7) | 0.1658(8) | 1 |
| S3 | 2i | 0.4771(1) | 0.1600(4) | 0.0320(6) | 1 |
| S4 | 2i | 0.2227(5) | 0.3824(3) | 0.0471(4) | 1 |
| S5 | 2i | 0.4427(4) | 0.6694(3) | 0.2116(3) | 1 |
| S6 | 2i | 0.4119(6) | 0.4914(3) | 0.3493(6) | 1 |
| S7 | 2i | 0.7905(3) | 0.4906(2) | 0.1926(3) | 1 |
| S8 | 2i | 0.1708(6) | 0.8406(4) | 0.4033(4) | 1 |
| S9 | 2i | 0.2455(7) | 0.1721(1) | 0.4538(7) | 1 |
| S10 | 2i | 0.1655(1) | 0.6781(5) | 0.5640(1) | 1 |
| S11 | 2i | 0.6719(6) | 0.0228(9) | 0.3124(1) | 1 |
| P1 | 2i | 0.1728(8) | 0.2062(6) | 0.0346(7) | 1 |
| P2 | 2i | 0.4691(2) | 0.5071(2) | 0.2034(6) | 1 |
| P3 | 2i | 0.8902(7) | 0.1600(5) | 0.4415(5) | 1 |
| Li1 | 2i | 0.1988(1) | 0.3310(3) | 0.4080(6) | 1 |
| Li2 | 2i | 0.0401(2) | 0.6381(7) | 0.3657(7) | 1 |
| Li3 | 2i | 0.7460(1) | 0.2160(1) | 0.2400(1) | 1 |
| Li4 | 2i | 0.2720(2) | 0.0580(1) | 0.2300(9) | 1 |
| Li5 | 2i | 0.0501(9) | 0.6410(1) | 0.1632(4) | 1 |
| Li6 | 2i | 0.6290(1) | 0.3630(1) | 0.0020(7) | 1 |
| Li7 | 2i | 0.4330(6) | 0.1390(1) | 0.6340(1) | 1 |

| Li_4_P_2_S_6_ (space group *P 63/m c m*)  λ = 0.82657 Å lattice parameter a = 6.0703(8) Å, c = 6.1223(1) Å  *R_wp_* = 4.787 %, Goodness of fit = 1.48  Phase fraction : 27.4 wt% | | | | | |
| --- | --- | --- | --- | --- | --- |
| Atom | Wyckoff position | Atomic coordinates | | | *Occ.* |
|  |  | *x* | *y* | *z* |  |
| S | 6g | 0.3237(2) | 0 | 0.25 | 1 |
| P | 4e | 0 | 0 | 0.1715(1) | 0.5 |
| Li | 4d | 0.3333(3) | 0.6666(7) | 0 | 1 |

**Supplementary Table 9**. Crystallographic data of the as-prepared AlPS_4_. The lattice parameters, fractional atomic coordinates, occupancies are obtained from the Rietveld refinements against the XRD data.

| AlPS_4_ (space group *P -4 2 c*)  λ = 1.54051 Å lattice parameter a = 5.6825(1) Å, c = 9.1103(8) Å  *R_wp_* = 3.916 %, Goodness of fit = 2.28  Impurity phase: 11.2 wt% of Al_4_(P_2_S_6_)_3_ | | | | | |
| --- | --- | --- | --- | --- | --- |
| Atom | Wyckoff position | Atomic coordinates | | | *Occ.* |
|  |  | *x* | *y* | *z* |  |
| S | 8n | 0.2105(5) | 0.2786(8) | 0.1231(4) | 1 |
| P | 2d | 0 | 0.5 | 0.25 | 1 |
| Al | 2a | 0 | 0 | 0.25 | 1 |

**Supplementary Table 10**. Crystallographic data of the as-prepared Li_2.82_Al_0.06_PS_4_. The lattice parameters, fractional atomic coordinates, occupancies are obtained from the Rietveld refinements against the SXRD data.

| Li_2.828_Al_0.057_PS_4_ (space group *P n m a*)  λ = 1.54051 Å lattice parameter a = 12.9057(1) Å, b = 8.1417(3) Å, c = 6.1369(2) Å  *R_wp_* = 8.265%, Goodness of fit = 2.73 | | | | | |
| --- | --- | --- | --- | --- | --- |
| Atom | Wyckoff position | Atomic coordinates | | | *Occ.* |
|  |  | *x* | *y* | *z* |  |
| S1 | 8d | 0.1566(6) | 0.0388(8) | 0.2812(7) | 1 |
| S2 | 4c | 0.9300(3) | 0.25 | 0.2617(8) | 1 |
| S3 | 4c | 0.1022(8) | 0.25 | 0.8051(8) | 1 |
| P | 4c | 0.0859(4) | 0.25 | 0.1722(6) | 1 |
| Al | 4b | 0 | 0 | 0.5 | 0.2292(8) |
| Li1 | 8d | 0.3452(1) | 0.9477(1) | 0.4499(9) | 1 |
| Li2 | 4b | 0 | 0 | 0.5 | 0.5414(9) |
| Li3 | 4c | 0.9960(1) | 0.25 | 0.8890(1) | 0.2865(5) |

**Supplementary Table 11**. Crystallographic data of the as-prepared Li_7_P_3_S_11_. The lattice parameters, fractional atomic coordinates, occupancies are obtained from the Rietveld refinements against the XRD data.

| Li_7_P_3_S_11_ (space group *P −1*)  λ = 1.54051 Å lattice parameter a = 6.0737(2) Å, b = 12.3049(1) Å, c = 12.5403(9) Å  *R_wp_* = 2.743 %, Goodness of fit = 1.05 | | | | | |
| --- | --- | --- | --- | --- | --- |
| Atom | Wyckoff position | Atomic coordinates | | | *Occ.* |
|  |  | *x* | *y* | *z* |  |
| S1 | 2i | 0.0969(1) | 0.8606(1) | 0.1195(7) | 1 |
| S2 | 2i | 0.0713(1) | 0.1765(1) | 0.1658(2) | 1 |
| S3 | 2i | 0.4771(3) | 0.1600(5) | 0.0320(8) | 1 |
| S4 | 2i | 0.2227(9) | 0.3824(6) | 0.0471(5) | 1 |
| S5 | 2i | 0.4427(5) | 0.6694(1) | 0.2116(4) | 1 |
| S6 | 2i | 0.4119(8) | 0.4914(6) | 0.3493(3) | 1 |
| S7 | 2i | 0.7905(8) | 0.4906(2) | 0.1926(6) | 1 |
| S8 | 2i | 0.1708(8) | 0.8406(1) | 0.4033(8) | 1 |
| S9 | 2i | 0.2455(7) | 0.1721(4) | 0.4538(1) | 1 |
| S10 | 2i | 0.1655(5) | 0.6781(1) | 0.5640(8) | 1 |
| S11 | 2i | 0.6719(2) | 0.0228(8) | 0.3124(3) | 1 |
| P1 | 2i | 0.1728(9) | 0.2062(4) | 0.0346(3) | 1 |
| P2 | 2i | 0.4691(3) | 0.5071(9) | 0.2034(4) | 1 |
| P3 | 2i | 0.8902(7) | 0.1600(4) | 0.4415(6) | 1 |
| Li1 | 2i | 0.1988(1) | 0.3310(1) | 0.4080(4) | 1 |
| Li2 | 2i | 0.0401(1) | 0.6381(6) | 0.3657(2) | 1 |
| Li3 | 2i | 0.7460(9) | 0.2160(3) | 0.2400(1) | 1 |
| Li4 | 2i | 0.2720(9) | 0.0580(8) | 0.2300(1) | 1 |
| Li5 | 2i | 0.0501(6) | 0.6410(6) | 0.1632(4) | 1 |
| Li6 | 2i | 0.6290(5) | 0.3630(6) | 0.0020(6) | 1 |
| Li7 | 2i | 0.4330(2) | 0.1390(2) | 0.6340(5) | 1 |

**Supplementary Table 12**. Crystallographic data of LPS w/o Al_2_S_3_. The lattice parameters, fractional atomic coordinates, occupancies are obtained from the Rietveld refinements against the XRD data.

| Li_3_PS_4_ (space group *P n m a*)  λ = 1.54051 Å lattice parameter a = 12.8930(3) Å, b = 8.1362(6) Å, c = 6.1368(9) Å  *R_wp_* = 4.144 %, Goodness of fit = 2.04  Phase fraction : 51.3 wt% | | | | | |
| --- | --- | --- | --- | --- | --- |
| Atom | Wyckoff position | Atomic coordinates | | | *Occ.* |
|  |  | *x* | *y* | *z* |  |
| S1 | 8d | 0.1524(5) | 0.0452(3) | 0.2236(6) | 1 |
| S2 | 4c | 0.4307(1) | 0.25 | 0.2620(9) | 1 |
| S3 | 4c | 0.0992(8) | 0.25 | 0.6701(7) | 1 |
| P | 4c | 0.0788(8) | 0.25 | 0.3169(1) | 1 |
| Li1 | 8d | 0.3058(4) | 0.0189(2) | 0.1180(7) | 1 |
| Li2 | 4b | 0 | 0 | 0.5 | 0.75 |
| Li3 | 4c | 0.0421(1) | 0.25 | 0.4632(8) | 0.25 |

| Li_7_P_3_S_11_ (space group *P −1*)  λ = 1.54051 Å lattice parameter a = 6.0513(5) Å, b = 12.3669(5) Å, c = 12.5398(5) Å  *R_wp_* = 4.144 %, Goodness of fit = 2.04  Phase fraction : 48.7 wt% | | | | | |
| --- | --- | --- | --- | --- | --- |
| Atom | Wyckoff position | Atomic coordinates | | | *Occ.* |
|  |  | *x* | *y* | *z* |  |
| S1 | 2i | 0.2156(5) | 0.8660(5) | 0.1050(5) | 1 |
| S2 | 2i | 0.1259(8) | 0.1571(1) | 0.1501(8) | 1 |
| S3 | 2i | 0.6742(2) | 0.2071(1) | 0.0340(4) | 1 |
| S4 | 2i | 0.2937(1) | 0.3895(6) | 0.0998(1) | 1 |
| S5 | 2i | 0.5493(5) | 0.6920(8) | 0.2234(1) | 1 |
| S6 | 2i | 0.4850(7) | 0.4845(5) | 0.3428(2) | 1 |
| S7 | 2i | 0.8865(3) | 0.5079(8) | 0.1995(6) | 1 |
| S8 | 2i | 0.1795(7) | 0.8347(9) | 0.3936(1) | 1 |
| S9 | 2i | 0.3159(8) | 0.1838(2) | 0.4488(1) | 1 |
| S10 | 2i | 0.1754(4) | 0.6771(1) | 0.5969(9) | 1 |
| S11 | 2i | 0.6668(1) | 0.0379(2) | 0.3465(7) | 1 |
| P1 | 2i | 0.1944(5) | 0.2131(6) | 0.0071(4) | 1 |
| P2 | 2i | 0.6374(3) | 0.5545(7) | 0.2064(6) | 1 |
| P3 | 2i | 0.0204(2) | 0.1523(8) | 0.4291(1) | 1 |
| Li1 | 2i | 0.0947(4) | 0.4278(2) | 0.4895(1) | 1 |
| Li2 | 2i | 0.4713(3) | 0.6088(6) | 0.2939(1) | 1 |
| Li3 | 2i | 0.4202(4) | −0.1153(7) | 0.2817(1) | 1 |
| Li4 | 2i | −0.6459(1) | −0.1889(8) | −0.3307(6) | 1 |
| Li5 | 2i | 0.6983(9) | 0.6907(1) | −0.0227(8) | 1 |
| Li6 | 2i | 0.6222(1) | 0.4268(3) | −0.0761(1) | 1 |
| Li7 | 2i | 0.9326(2) | 0.4973(8) | 0.5359(3) | 1 |

**Supplementary Table 13**. Room temperature $\sigma_{{Li}^{+}}$ of the GCs at cold pressed state prepared by mechanical milling and annealing.

| Material | $\sigma_{{Li}^{+}}$ (mS cm^−1^) | Ref |
| --- | --- | --- |
| LPS7228 | 13.2 | This work |
| Ga-LPS7228 | 7.45 | This work |
| Si-LPS7327 | 8.57 | This work |
| 60Li_2_S∙40P_2_S_5_ | 0.0032 | 2 |
| 67Li_2_S·33P_2_S_5_ | 0.038 | 2 |
| 70Li_2_S·30P_2_S_5_ | 0.037 | 2 |
| 75Li_2_S·25P_2_S_5_ | 0.28 | 2 |
| 80Li_2_S·20P_2_S_5_ | 0.13 | 2 |
| 75Li_2_S∙25P_2_S_5_ | 1.33 | 3 |
| Li_3_PS_4_ | 0.164 | 4 |
| Li_3.2_P_0.8_Sn_0.2_S_4_ | 1.21 | 5 |
| Li_7_P_3_S_11_ | 0.81 | 6 |
| Li_6.988_P_2.994_Nb_0.2_S_10.934_O_0.6_ | 2.82 | 7 |
| 99.5(70Li_2_S∙30P_2_S_5_)-0.5FeS_2_ | 2.22 | 8 |
| Li_7_P_2.9_Sb_0.1_S_10.75_O_0.25_ | 1.61 | 9 |
| Li_3.06_P_0.98_Zn_0.02_S_3.98_O_0.02_ | 1.12 | 10 |
| Li_7_P_2.9_Ce_0.2_S_10.9_Cl_0.3_ | 3.2 | 11 |
| Li_7_P_2.88_Nb_0.12_S_10.7_O_0.3_ | 3.59 | 6 |

**Supplementary Table 14**. Summary of the sulfide electrolyte-based ASSBs’ performance at 0.1 C and room temperature.

|  | Cell type | Lifespan/cycles | Active material loading/mg cm^−2^ | 1^st^ capacity/mAh g^−1^ | Initial Coulombic efficiency | 50^th^ capacity retention | 100^th^ capacity retention | Ref |
| --- | --- | --- | --- | --- | --- | --- | --- | --- |
| Bare Li anode | LCO\|LPS7228\|Li | 200 | 8.92 | 141.7 | 92.4% | 94.5% | 90.6% | This work |
|  | LCO\|gc-Li_3.2_P_0.8_Sn_0.2_S_4_\|Li | 60 | 8.92 | 118.4 | 96.2% | 82.4% | / | 12 |
| Multilayer electrolyte | LCO\|Li_6_PS_5_Cl\|LPSCl_0.3_F_0.7_@Li | 50 | 8.92 | 122 | 89% | 95% | / | 13 |
|  | LCO\|Li_10_GeP_2_S_12_/LPSI-20Sn\|Li | 50 | 8.92 | 123.7 | 91% | 88.5% | / | 14 |
|  | LCO\|Li_10_GeP_2_S_12_/Li_3.06_P_0.98_Zn_0.02_S_3.98_O_0.02_\|Li | 100 | NA | 139.1 | 83% | 86% | 81% | 10 |
| Modified Li anode | LCO\|Li_7_P_3_S_11_\|LiF@Li | 100 | 3.6 | 118.9 | 79.3% | 86.4% | 81.4% | 15 |
|  | LCO\|Li_3_PS_4_\|Li*_x_*SiS*_y_*-Li | 100 | 8.92 | 126 | 87.9% | 97.6% | 79.4% | 16 |
|  | LCO\|Li_10_GeP_2_S_12_\|LiH_2_PO_4_@Li | 500 | 5.5 | 136.7 | 85.9% | 95.1% | 87.8% | 17 |
|  | LCO\|Li_6_PS_5_Cl\|Li_2_Se-NR/Li | 100 | 7.53 | 123.4 | 93% | 81.5% | 76% | 18 |
|  | LCO\|Li_6_PS_5_Cl\|Li_3_N/LiF@Li | 80 | NA | 125.2 | 93.57% | 94% | / | 19 |

Note:

LCO, LiCoO_2_;

LPSCl_0.3_F_0.7_, Li_6_PSCl_0.3_F_0.7_;

LPSI-20Sn, Li_6.2_P_0.8_Sn_0.2_S_5_I;

Li*_x_*SiS*_y_*-Li, Li*_x_*SiS*_y_* layer on the surface of Li anode, where Li*_x_*SiS*_y_* is fabricated by the solution-based reaction through Li, Li_2_S_8_ and SiCl_4_;

Li_2_Se-NR/Li, Li_2_Se nanorods on the surface of Li anode.

**Supplementary Table 15**. Data for the calculation of *E_m_* and *P_m_*.

| Cathode | Electrolyte | Anode | Discharge specific capacity *q* (mA h g^−1^) | Current *I* (mA) | Average discharge voltage *U* (V) | Mass (mg) | | | | *E_m_* (Wh kg^−1^) | *P_m_* (W kg^−1^) | Temp. (°C) | Ref |
| --- | --- | --- | --- | --- | --- | --- | --- | --- | --- | --- | --- | --- | --- |
|  |  |  |  |  |  | Cathode *m_c_* | Electrolyte *m_e_* | Anode *m_a_* | Cathode loading *m_l_* |  |  |  |  |
| LCO | Li_10_GeP_2_S_12_-Li_3_PS_4_ | Li-In | 136.15 | 0.15 | 3.28 | 15 | 150 | 100 | 12.75 | 21.49 | 1.86 | 30 | 20 |
|  |  |  | 132.73 | 0.3 | 3.27 |  |  |  |  | 20.91 | 3.71 |  |  |
|  |  |  | 120.29 | 0.75 | 3.25 |  |  |  |  | 18.83 | 9.21 |  |  |
|  |  |  | 111.22 | 1.05 | 3.19 |  |  |  |  | 17.08 | 12.65 |  |  |
|  |  |  | 95.96 | 1.5 | 3.15 |  |  |  |  | 14.52 | 17.81 |  |  |
|  |  |  | 56.91 | 3 | 2.94 |  |  |  |  | 8.05 | 33.29 |  |  |
|  |  |  | 11.62 | 7.5 | 2.75 |  |  |  |  | 1.54 | 77.76 |  |  |
| LCO | Li_10_GeP_2_S_12_ | In | 134.84 | 0.1 | 3.27 | 10 | 80 | 45.9 | 8 | 25.93 | 2.4 | 25 | 21 |
|  |  |  | 120.88 | 0.2 | 3.25 |  |  |  |  | 23.16 | 4.79 |  |  |
|  |  |  | 107.95 | 0.5 | 3.16 |  |  |  |  | 20.06 | 11.61 |  |  |
|  |  |  | 91.93 | 1 | 2.98 |  |  |  |  | 16.14 | 21.95 |  |  |
|  |  |  | 72.17 | 2 | 2.7 |  |  |  |  | 11.46 | 39.71 |  |  |
|  |  |  | 34.37 | 5 | 2.26 |  |  |  |  | 4.63 | 84.23 |  |  |
| LCO | Li_9.6_P_3_S_12_ | Graphite |  |  |  |  |  |  |  | 32.6 | 4.01 | 25 | 22 |
|  |  |  |  |  |  |  |  |  |  | 30.92 | 36.25 |  |  |
|  |  |  |  |  |  |  |  |  |  | 30.36 | 71.6 |  |  |
|  |  |  |  |  |  |  |  |  |  | 26.83 | 169.84 |  |  |
|  |  |  |  |  |  |  |  |  |  | 24.99 | 273.52 |  |  |
|  |  |  |  |  |  |  |  |  |  | 24.58 | 343.47 |  |  |
|  |  |  |  |  |  |  |  |  |  | 19.54 | 647.34 |  |  |
|  |  |  |  |  |  |  |  |  |  | 16.38 | 1042.52 |  |  |
|  |  |  |  |  |  |  |  |  |  | 12.15 | 1465.22 |  |  |
|  |  |  |  |  |  |  |  |  |  | 8.69 | 1923.8 |  |  |
|  |  |  |  |  |  |  |  |  |  | 6.22 | 2310.34 |  |  |
|  |  |  |  |  |  |  |  |  |  | 4.7 | 2768.05 |  |  |
|  |  |  |  |  |  |  |  |  |  | 3.36 | 3033.42 |  |  |
|  |  |  |  |  |  |  |  |  |  | 2.4 | 3475.85 |  |  |
|  |  |  |  |  |  |  |  |  |  | 1.61 | 3809.08 |  |  |
| LCO | LiI-Li_2_S-P_2_S_5_ | Graphite |  |  |  |  |  |  |  | 176.89 | 6.22 | 25 | 23 |
|  |  |  |  |  |  |  |  |  |  | 135.35 | 60.04 |  |  |
|  |  |  |  |  |  |  |  |  |  | 63.35 | 115.3 |  |  |
|  |  |  |  |  |  |  |  |  |  | 30.53 | 175.3 |  |  |
| LCO | Li_10_GeP_2_S_12_ | Li |  |  |  |  |  |  |  | 19.54 | 2.03 | 25 | 17 |
|  |  |  |  |  |  |  |  |  |  | 16.99 | 4.01 |  |  |
|  |  |  |  |  |  |  |  |  |  | 15.01 | 10.18 |  |  |
|  |  |  |  |  |  |  |  |  |  | 6.91 | 20.11 |  |  |
| NCM622 | Li_3_PS_4_ | Li |  |  |  |  |  |  |  | 43.62 | 7.11 | 25 | 24 |
|  |  |  |  |  |  |  |  |  |  | 34.93 | 17.22 |  |  |
|  |  |  |  |  |  |  |  |  |  | 17.88 | 31.26 |  |  |
| NCM622 | Li_3_PS_4_ | Li |  |  |  |  |  |  |  | 97.53 | 19.32 | 25 | 24 |
|  |  |  |  |  |  |  |  |  |  | 67.16 | 44.93 |  |  |
|  |  |  |  |  |  |  |  |  |  | 37.57 | 84.78 |  |  |
| LiAl_0.08_Co_0.92_O_2_ | Li_3.25_Ge_0.25_P_0.75_S_4_ | Li-In | 140.09 | 0.048 | 3.32 | 10 | 150 | 60 | 7 | 14.8 | 0.72 | 25 | 25 |
|  |  |  | 125.14 | 0.48 | 3.26 |  |  |  |  | 12.98 | 7.11 |  |  |
|  |  |  | 113.63 | 0.96 | 3.22 |  |  |  |  | 11.63 | 14.03 |  |  |
|  |  |  | 98.46 | 1.92 | 3.00 |  |  |  |  | 9.39 | 26.17 |  |  |
|  |  |  | 80.76 | 4.8 | 2.92 |  |  |  |  | 7.5 | 63.69 |  |  |
| Li_2_S | Li_3_PS_4_ | Li | 945.93 | 0.1 | 1.86 | 5.43 | 12 | 1.88 | 2 | 182.62 | 9.65 | 25 | 26 |
|  |  |  | 688.32 | 0.2 | 1.83 |  |  |  |  | 130.46 | 18.95 |  |  |
|  |  |  | 528.78 | 0.4 | 1.8 |  |  |  |  | 98.31 | 37.18 |  |  |
|  |  |  | 358.96 | 1.0 | 1.7 |  |  |  |  | 63.32 | 88.2 |  |  |
| FeS_2_ | 77.5Li_2_S-22.5P_2_S_5_+self-healing polymer | Li-In | 1014.51 | 0.2 | 1.5 | 5 | 10 | 20 | 2.27 | 98.7 | 8.57 | 25 | 27 |
|  |  |  | 971.49 | 0.4 | 1.48 |  |  |  |  | 93.17 | 16.9 |  |  |
|  |  |  | 882.56 | 1 | 1.43 |  |  |  |  | 82.12 | 40.99 |  |  |
|  |  |  | 772.13 | 2 | 1.38 |  |  |  |  | 69.11 | 78.86 |  |  |
|  |  |  | 583.69 | 4 | 1.29 |  |  |  |  | 48.71 | 147.05 |  |  |
|  |  |  | 408.73 | 8 | 1.2 |  |  |  |  | 31.81 | 274.29 |  |  |
| LiTiS_2_ | Li_3_PS_4_ | Li_4_Ti_5_O_12_ | 142.62 | 0.08 | 0.64 | 4.5 | 7.73 | 10 | 1.4535 | 5.94 | 2.29 | 30 | 28 |
|  |  |  | 122.03 | 0.28 | 0.57 |  |  |  |  | 4.58 | 7.23 |  |  |
|  |  |  | 109.09 | 0.58 | 0.53 |  |  |  |  | 3.81 | 13.95 |  |  |
|  |  |  | 98.08 | 1 | 0.5 |  |  |  |  | 3.21 | 22.53 |  |  |
|  |  |  | 86.57 | 1.42 | 0.47 |  |  |  |  | 2.64 | 29.75 |  |  |
|  |  |  | 65.01 | 2.8 | 0.4 |  |  |  |  | 1.7 | 50.38 |  |  |
|  |  |  | 33.88 | 5.8 | 0.3 |  |  |  |  | 0.68 | 79.57 |  |  |
| NCM622 | LPS7228 | μSi | 170.45 | 0.63 | 3.8 | 43.75 | 8 | 2.2 | 35 | 420.2 | 44.37 | 30 | This work |
|  |  |  | 148.48 | 1.26 | 3.49 |  |  |  |  | 336.2 | 81.51 |  |  |
|  |  |  | 101.27 | 3.15 | 3.32 |  |  |  |  | 218.1 | 193.85 |  |  |
|  |  |  | 58.70 | 6.3 | 3.28 |  |  |  |  | 124.9 | 383.02 |  |  |
|  |  |  | 38.26 | 9.45 | 3.25 |  |  |  |  | 80.67 | 569.28 |  |  |

Note:

1. Calculation equations for *E_m_* and *P_m_* shown as follow.

$$\begin{matrix} E_{m}=\frac{Uqm_{l}}{m_{c}+m_{e}+m_{a}}; \\ P_{m}=\frac{UI}{m_{c}+m_{e}+m_{a}} \end{matrix}$$

2. Description of approximations and assumptions made to calculate data for comparison where indicated.

Ref 20, Ref 25, Ref 26, Ref 28: The value of *q* and *U* are extracted from graph;

Ref 21: The value of *q* and U are extracted from graph, and the *m_a_* (Indium anode) is calculated based on 0.125 mm thick and 8 mm diameter;

Ref 22, Ref 23, Ref 17: The value of *E_m_* and *P_m_* are extracted from a Ragone plot in Ref 24;

Ref 24: The value of *E_m_* and *P_m_* are extracted from a Ragone plot displayed in the paper;

Ref 27: The value of *q* is extracted from graph. The value of *U* is assumed based on the electrode materials and discharge rates. *m_a_* is assumed as 20 mg based on the given specific energy.

**Supplementary Notes**

**Supplementary Note 1: Phase composition of Al-GCs**

Supplementary Figure 1 shows the SXRD patterns and Raman spectra of the as-prepared Al-GCs. For both LPS7525 and LPS7426, the SXRD patterns are in good accordance with that of *β*-Li_3_PS_4_ (hereinafter referred to as Li_3_PS_4_ unless otherwise stated), which is also confirmed by the Raman spectra showing only one peak at 418 cm^−1^ of PS_4_^3−^. Further decreasing the Li_2_S : P_2_S_5_ ratio results in the emergence of new SXRD peaks at 6.9, 7.9, 8.2, 12.7°, and new Raman peak at 402 cm^−1^. According to the prior reports and the standard XRD pattern,^29^ such new peaks can be attributed to Li_7_P_3_S_11_ analog phase consisted by PS_4_^3−^ and P_2_S_7_^4−^. The Rietveld refinement results in Supplementary Figure 1d indicate that the proportion of Li_7_P_3_S_11_ and Li_3_PS_4_ analog phase in LPS7327 are 5.8 and 94.2 wt %, respectively. No other new phase emerges when the Li_2_S : P_2_S_5_ ratio decreases to 72 : 28, except the proportion of Li_7_P_3_S_11_ analog phase increases significantly to 72.9 wt %. Further decreasing Li_2_S content generates another new phase of Li_4_P_2_S_6_ analog, which is verified by the Raman spectrum and new SXRD peaks at 9.0, 14.4, 17.2°. According to the Rietveld refinement analysis, the proportion of Li_3_PS_4_, Li_7_P_3_S_11_ and Li_4_P_2_S_6_ analog phase accounts for 24.7, 47.9, 27.4 wt % for LPS7129, and evolves to 22.6, 32.0, 45.4 wt % for LPS7030. Attempt to decrease the Li_2_S molar percentage to 69 % leads to the rise of aluminum thiophosphates (AlPS*_x_*), i.e., AlPS_4_ and Al_4_(P_2_S_6_)_3_,^30^ which can be prepared successfully by replacing all the Li_2_S by Al_2_S_3_ (Supplementary Figure 1g).

Based on the above analysis, the phase composition of Al-GCs is visually shown in Supplementary Figure 1h. Conclusively, with the ratio of Li_2_S : P_2_S_5_ decreases from 75 : 25 to 65 : 35, the PS_4_^3−^ tetrahedrons undergo the transformation of PS_4_^3−^ → P_2_S_7_^4−^ → P_2_S_6_^4−^, corresponding to the crystal structure transformation of Li_3_PS_4_ analog phase → Li_7_P_3_S_11_ analog phase → Li_4_P_2_S_6_ analog phase.

**Supplementary Note 2:** **Estimation of the P/S ratio**

The stoichiometry (mol%) of LPS7228 is 72 mol% Li_2_S, 28 mol% P_2_S_5_ and 4 mol% Al_2_S_3_. Assuming the total weight of the components is 1g, the moles of Li_2_S and P_2_S_5_ in the raw materials are:

$$\text{M}_{1}(\text{Li}_{2}\text{S})=\frac{1\text{g}}{72\text{mol\%}\times46\text{gmol}^{\text{-1}}+28\text{mol\%}\times222\text{gmol}^{\text{-1}}+4\text{mol\%}\times150\text{gmol}^{\text{-1}}}\times72\text{mol\%}=7.106\text{mmol}$$

$$\text{M}_{1}\text{(P}_{2}\text{S}_{5}\text{)}=\frac{1\text{g}}{72\text{mol\%}\times46\text{gmol}^{\text{-1}}+28\text{mol\%}\times222\text{gmol}^{\text{-1}}+4\text{mol\%}\times150\text{gmol}^{\text{-1}}}\times28\text{mol\%}=2.763\text{mmol}$$

The moles of Li_2_S and P_2_S_5_ needed to generate 27.1 wt% Li_2.82_Al_0.06_PS_4_ (180.36 g mol^−1^) are:

$$\text{M}_{2}\text{(Li}_{2}\text{S)}=\frac{1\text{g}\times0.271}{180.36\text{gmol}^{\text{-1}}}\times\frac{2.82}{2}=2.115\text{mmol}$$

$$\text{M}_{2}\text{(P}_{2}\text{S}_{5}\text{)}=\frac{1\text{g}\times0.271}{180.36\text{gmol}^{\text{-1}}}\times\frac{1}{2}=0.75\text{mmol}$$

The P/S ratio in the remaining raw material is:

$$\frac{\text{P}}{\text{S}}=\frac{2\times(\mathrm{M}_{1}(\mathrm{P}_{2}\mathrm{S}_{5})-\mathrm{M}_{2}(\mathrm{P}_{2}\mathrm{S}_{5}))}{5\times(\mathrm{M}_{1}(\mathrm{P}_{2}\mathrm{S}_{5})-\mathrm{M}_{2}(\mathrm{P}_{2}\mathrm{S}_{5}))+(\mathrm{M}_{1}(\text{Li}_{2}\text{S})-\mathrm{M}_{2}(\text{Li}_{2}\text{S}))}=0.267$$

**Supplementary Note 3:**

Li_3_PS_4_ is composed of isolated PS_4_^3−^ tetrahedra that is connected with LiS_6_ octahedra via common edges. There are three different Li sites as shown in Supplementary Figure 3a: a fully occupied tetrahedral site (8*d*, 100%, marked as Li1), a partially occupied octahedral site (4*b*, 70%, marked as Li2), and a partially occupied tetrahedral site (4*c*, 30%, marked as Li3).^31^

**Supplementary Note 4:**

As shown in Supplementary Figure 6b, during Li_7_P_3_S_11_ nucleation growth, the binding energy (*E_bind_*) of Li_7_P_3_S_11_@Li_2.82_Al_0.06_PS_4_ is −4.8 eV nm^−2^, 1.3 times lower than that of Li_7_P_3_S_11_@Li_7_P_3_S_11_, −6.2 eV nm^−2^, indicating that Li_7_P_3_S_11_ prefers to crystallize on Li_2.82_Al_0.06_PS_4_ crystal faces, which is in well agreement with the experimental observation in Fig. 2f.

**Supplementary Note 5:**

Temperature-dependent electrochemical impedance spectroscopy (EIS) are conducted to derive the Arrhenius plots of Al-GCs (Supplementary Figure 8a below). The activation energies (*E_a_*) of Al-GCs are determined based on the Arrhenius equation:

$\sigma=\frac{\sigma_{0}}{T}\exp(\frac{-E_{a}}{k_{B}T})$ (1)

where, $\sigma$, $\sigma_{0}$ $T$, $k_{B}$ denote the Li^+^ conductivity, exponential prefactor, absolute temperature, Boltzmann constant, respectively.

As shown in Supplementary Figure 8b, a value of 0.310 eV is reached for LPS7525 and decreases to 0.305 eV for LPS7426. With decreasing Li_2_S : P_2_S_5_ ratio, *E_a_* decreases to 0.304 eV for LPS7327, and reaches a minimum of 0.291 eV for LPS7228. The decrease of *E_a_* may be contributed to the formation of Li_3−3_*_x_*Al*_x_*PS_4_ with increased carrier’s density and hetero-nanodomains. Further reducing the Li_2_S : P_2_S_5_ ratio increases *E_a_* to 0.316 eV (LPS7129), 0.352 eV (LPS7030), 0.375 eV (LPS6931) and 0.398 eV (LPS6535). According to the species evolution of Al-GCs, further decreasing Li_2_S content generates large amount of poor Li^+^ conductor of Li_4_P_2_S_6_ (*E_a_* = 0.46 eV)^32^ and even non-Li^+^ conductor aluminum thiophosphates, blocking the Li^+^ migration and thus leading to a large *E_a_*.

**Supplementary Note 6:**

Low electronic conductivity ($\sigma_{e}$) is important for solid electrolytes, to minimize self-discharge, and reduce dendritic lithium formation. The DC polarization curves of Al-GCs under an applied voltage of 0.1 V are shown in Supplementary Figure 9a, based on which the $\sigma_{e}$ of Al-GCs (from LPS7525 to LPS6535) are calculated to be 4.74, 4.83, 5.76, 4.40, 5.67, 3.84, 4.76, 5.46 × 10^−9^ S cm^−1^ (Supplementary Figure 9b), respectively, which are similar to that of reported GCs.

**Supplementary Note 7:**

The ∆G_r_ of Ga-doped Li_3_PS_4_ and Si-doped Li_3_PS_4_ are −136.21 and −138.64 meV atom^−1^, respectively, which are nearly the same as Al-doped Li_3_PS_4_. Using the same synthesis condition, Ga-GCs and Si-GCs are successfully prepared. As shown in Supplementary Figure 11a and 11f, the phase composition analysis of Ga-GCs and Si-GCs suggest that the similar crystal structure transformation of Li_3_PS_4_ analog phase → Li_7_P_3_S_11_ analog phase → Li_4_P_2_S_6_ analog phase also can be found in Ga-GCs and Si-GCs. The $\sigma_{{Li}^{+}}$ of Ga-LPS7426 is 0.613 mS cm^−1^, which boosts up to 1.49 mS cm^−1^ for Ga-LPS7327 and to the highest value of 7.45 mS cm^−1^ for Ga-LPS7228, and then gradually reduces to 5.07 mS cm^−1^ (Ga-LPS7129) and 1.57 mS cm^−1^ (Ga-LPS7030) (Supplementary Figure 11b). The measured $\sigma_{{Li}^{+}}$ of the Si-GCs also presents the similar variation tendency as that of Ga-GCs, and the highest value of 8.57 mS cm^−1^ is obtained for Si-LPS7327 (Supplementary Figure 11g). Meanwhile, the hetero-nanodomains resulted from sequential crystallization and heterogeneous growth induced by Ga_2_S_3_ and SiS_2_ are investigated by cryo-TEM, as shown in Supplementary Figure 11 c-e and h-j.

**Supplementary Note 8: Indispensability of the nucleation-accelerant**

In this section, the indispensability of nucleation-accelerant in sequential crystallization and heterogeneous growth mechanism is carefully validated. To verify this, a GC with a composition of 72.5 mol% Li_2_S and 27.5 mol% P_2_S_5_ (referred as LPS w/o Al_2_S_3_), just the theoretical ratio needed to produce exactly 72.9 wt% Li_7_P_3_S_11_ and 27.1 wt% Li_3_PS_4_, is firstly processed. In addition, after DFT calculation, we find that Li_3−2_*_x_*Ca*_x_*PS_4_ also has very low ∆G_r_, indicating CaS could be used as nucleation-accelerant. To this end, CaS-tuned GCs (regarded as Ca-GCs) is also prepared.

The Rietveld refinement result in Supplementary Figure 12a indicates that the practical proportion of Li_3_PS_4_ and Li_7_P_3_S_11_ phase are 51.3 and 48.7 wt %, respectively. Such a fifty-fifty reaction probably results from the similar ∆G_r_ of Li_3_PS_4_ *vs.* Li_7_P_3_S_11_ (Supplementary Figure 3). Lower Li_7_P_3_S_11_ fraction means a poorer $\sigma_{{Li}^{+}}$ because P_2_S_7_^4−^ is more conductive than PS_4_^3−^. Worse still, the similar ∆G_r_ also suggests the simultaneous crystallization of Li_3_PS_4_ and Li_7_P_3_S_11_ separately, leading to the respective distribution of both phase (Supplementary Figure 12b), which is quite different from the Li_7_P_3_S_11_@Li_2.82_Al_0.06_PS_4_ hetero-nanodomains in LPS7228 (Fig. 2d). As a result, $\sigma_{{Li}^{+}}$ of LPS w/o Al_2_S_3_ is only 1.28 mS cm^−1^, 10 times lower than that of LPS7228.

CaS is used to tune GCs (Ca-GCs) under the same conditions as Al-GCs. It should be noted that the ∆G_r_ of Ca-doped Li_3_PS_4_ is nearly the same as Al-doped Li_3_PS_4_. Nevertheless, the XRD patterns in Supplementary Figure 13 demonstrate the pure Li_3_PS_4_ analogy even for the 20 mol% Ca-tuned GC (Ca-LPS7030), indicating that all the CaS participates in the solid-state reaction to form Ca-doped Li_3_PS_4_, i.e., Li_2.34_Ca_0.33_PS_4_, that is to say a much larger solution limit of Ca^2+^ than that of Al^3+^ in Li_3_PS_4_. As a consequence of the low ∆G_r_ and large solution limit, only Ca-doped Li_3_PS_4_ can be precipitated within Ca-GCs. Without neither the highly conductive P_2_S_7_^4−^ polyhedron nor the hetero-nanodomains resultant from the sequential crystallization and heterogeneous growth, very poor $\sigma_{{Li}^{+}}$ is obtained for Ca-GCs, for example, 0.032 mS cm^−1^ at room temperature. In higher dopant concentrations the complex defect pair of bound dopant cations (Ca^2+^ in this case) and vacancies are increased. Since the bound pairs are not easily separated, the available sites for Li^+^ hopping are decreased, resulting in both lower mobilities and decreased conductivity.

Consequently, it can be concluded that two functions of nucleation-accelerant are indispensable, that is, the nucleation-accelerants can greatly lower the energy of certain crystallite nucleation for its short burst nucleation, but also change the P/S ratio subsequently to initiate the nucleation of another crystallite.

**Supplementary Note 9:**

Symmetric Li|Al-GCs|Li cells are assembled to test the critical current density (CCD) at room temperature, and the results are shown in Supplementary Figure 14. For the sulfide electrolytes, due to the lithium dendrite growth with increasing current density, the cells show a voltage drop at a certain current value, which is estimated as the CCD. Accordingly, the CCD of Al-GCs from LPS7525 to LPS6535 at room temperature are 0.2, 0.25, 0.5, 0.95, 0.3, 0.2, 0.05 and 0.05 mA cm^−2^, respectively.

**Supplementary Note 10:**

As shown in Supplementary Figure 18b, there is no Raman signal at position 1#, but one peak at around 425 cm^−1^ associated with PS_4_^3−^ can be observed at position 2#. It is generally known that a change in polarizability during molecular vibration is an essential requirement to obtain Raman spectra of samples, however metal does not show the polarizability change and so no Raman spectra are obtained. Accordingly, we can conclude that the silver-like granules observed in Fig. 4d are deposits of lithium metal inside Li_6_PS_5_Cl.

**Supplementary Note 11:**

To figure out the stable cycle behavior, symmetric Li|LPS7228|Li cell after cycling at 0.2 mA cm^−2^ for 50 cycles is disassembled for XPS analysis. Supplementary Figure 19 shows the Li *1s*, S *2p*, P *2p* and Al *2p* XPS spectra of the pristine LPS7228 sample and the Li|LPS7228 interface from the as-cycled cell. For the S *2p* spectrum of pristine LPS7228, the peaks located at 161.7, 162.1 and 163.0 eV represent P−S−Li, P=S and P−S−P species, respectively.^33^ Meanwhile, the peak at 161.9 eV is assigned to Al−S species, which is also verified in Al *2p* spectrum (77.3 eV, Al−S). Correspondingly, the P *2p* spectrum demonstrates peaks of PS_4_^3−^ (132.1 eV) and P_2_S_7_^4−^ species (133.0 eV). For the as-cycled Li|LPS7228 interface, no significant changes are detected both in the P *2p* and S *2p* spectra with the exception of the presence of a weak signal in S *2p* spectrum located at 160.1 eV, corresponding to Li_2_S. However, for the Al *2p* spectrum, new peak located at 72.5 eV can be fitted, which suggests a partial reduction of Al.^34^ In addition, a new Li 1s peak is also simulated at 55.2 eV. Both the Al *2p* and Li *1s* spectra indicate the formation of Li−Al alloy according to the following equation,

20Li + 3Al_2_S_3_ → 9Li_2_S + 2LiAl_3_, *∆*G_r_ = −0.601 eV (2)

Actually, Li−Al alloy has been deliberately adopted as a functional interlayer between Li and liquid electrolytes to regulate the Li stripping/plating behavior.^35,36^ In our case, the stable Li polarization behavior can also be attributed to the in-situ formed Li−Al alloy to uniformize the Li^+^ flux during Li plating/stripping processes.^12^

**Supplementary Note 12:**

Experimentally, the electrochemical window (EW) is usually probed via the cyclic voltammetry (CV) method. According to the approach reported by Han et al,^37^ an asymmetric cell setup ((Al-GCs)-VGCF|Li_6_PS_5_Cl|Li/In) with lithium-indium alloy (acting as both counter and reference electrode) and a composite of Al-GCs with VGCF (weight ratio of Al-GC to VGCF is 70 : 30) is used as working electrode to obtain the intrinsic EW of Al-GCs.

Specifically, to probe the upper potential of the EW, the cell is cycled at a scan rate of 0.1 mV/s, starting at the open-circuit potential, up to 3.5 V and back to 1 V *vs*. Li^+^/Li and the cycle ends at the open-circuit potential. To probe the lower potential of the EW, the cell is cycled at a scan rate of −0.1 mV/s, starting at the open-circuit potential, down to 0.5 V and back to 2.0 V *vs*. Li^+^/Li. The EWs of Al-GCs extracted from Supplementary Figure 20a and 20b are shown in Supplementary Figure 20c. The EWs of Al-GCs are 2.02−2.7, 2.03−2.77, 1.94−2.93, 1.53−2.94, 1.59−2.99, 1.57−2.97, 1.47−2.95, 1.48−3.01 V *vs*. Li^+^/Li for LPS7525, LPS7426, LPS7327, LPS7228, LPS7129, LPS7030, LPS6931, LPS6535, respectively.

To test whether Al-GCs can match high-voltage NCM811, all-solid-state lithium batteries (ASSBs) using LPS7228 (30 wt%) + bare- or LiNbO_3_ coated NCM811 (70 wt%) cathode composite, Li_6_PS_5_Cl electrolyte and Li/In anode were fabricated and cycled at 0.1 C rate between 3.0 and 4.5 V *vs*. Li^+^/Li. As shown in Supplementary Figure 20d and 20e, the bare-NCM811|Li_6_PS_5_Cl|Li/In battery exhibits fast capacity fade with 52.5 % capacity retention over only 70 cycles, similar to the results reported by Koerver et al.^33^ This common phenomenon is cuased by the narrow thermodynamic intrinisic EW of sulfide electrolytes ranging from 1.7 to 2.9 V *vs*. Li^+^/Li, and the tendency for the high-voltage cathode oxidizing the sulfide electrolytes in physical contact, in particular at high charging potential.

Coating cathode active material particles with an electronically insulating/ionically conductive, chemically compatible material has been verified to address this problem effectively. Accordingly, the ASSB with LiNbO_3_ coated NCM811 cathode composite (Supplementary Figure 20f) demonstrates much better electrochemical performance, maintaining 80 % capacity retention over 70 cycles. Consequently, it can be concluded that although LPS7228 has a narrow EW, it can still match high-voltage cathode materials with stable coating. It should be noted that the cathode active materials used in this work are all coated with LiNbO_3_.

**Supplementary Note 13:**

Supplementary Figure 22a shows the cyclic voltammetry (CV) of the LiNbO_3_ coated LiCoO_2_|LPS7228|Li at a scan rate of 0.1 mV s^−1^, which exhibits well-defined redox peaks, corresponding to the main lithiation/delithiation process. The symmetry of oxidative/reductive peaks indicates a good reversibility of the intercalation/deintercalation process. As shown in Supplementary Figure 22b and S22c, the cycling performance of LiCoO_2_|LPS7228|Li ASSB at 0.5 C is evaluated. A reversible specific discharge capacity of 120.5 mAh g^−1^ is achieved at 0.5 C for the first cycle in the voltage range of 3.0 − 4.3 V with a high CE of 96.1 %. A reversible specific capacity of 103.7 mAh g^−1^ is maintained after 110 cycles, showing a good retention of 86.1 % with an average CE of 99.2 %, suggesting highly reversible Li^+^ intercalation/deintercalation. To understand the probable reasons for capacity fade, EIS measurements on the LiNbO_3_ coated LiCoO_2_|LPS7228|Li are conducted. Supplementary Figure 22d shows the Nyquist plots of the ASSB after the first and 110^th^ cycle. The ohmic resistances remain stable, while the interfacial resistances increase, accounting for the capacity fade with cycling.

**Supplementary Methods**

**Preparation of AlPS_4_** Stoichiometric amounts of Al (Alfa Aesar 99.9 %), P (Alfa Aesar 98.9 %), and S (Alfa Aesar 99.5 %) raw materials are mixed in an agate mortar. The mixture is then vacuum-sealed in a quartz tube and heated to 923 K. After holding at 923 K for 7 days and naturally cooling down to room temperature, AlPS_4_ is obtained.

**Preparation of Li_2.82_Al_0.06_PS_4_, Li_7_P_3_S_11_, LPS w/o Al_2_S_3_,** **Ca-GCs, Ga-GCs and Si-GCs.** Stoichiometric amounts of Li_2_S (Alfa Aesar 99.9 %), P_2_S_5_ (MACKLIN ≥ 99 %), and/or Al_2_S_3_ (Alfa Aesar 99.9 %), CaS (MACKLIN ≥ 99 %), Ga_2_S_3_ (Aladdin 99.999%), SiS_2_ (Aladdin 99.9%) raw materials are weighted and mixed in zirconia jars followed by mechanical milling for 24 hrs at a speed of 450 rpm using an apparatus (FRITSCH, Pulverisette7). Afterwards, the obtained powders are pressed into green pellets under 320 MPa. Then the pellets are sealed into a quartz tube filled with argon and heated at 300 °C for 8 hrs and naturally cooled down to room temperature.

**Preparation of Li_6_PS_5_Cl** Li_6_PS_5_Cl is prepared by the reaction of stoichiometric mixtures of Li_2_S (Alfa Aesar 99.9%), P_2_S_5_ (MACKLIN ≥ 99%), and LiCl (MACKLIN ≥ 99%). The starting materials are mixed in a sealed zirconia pot for 24 h at a speed of 600 rpm using a ball milling apparatus (FRITSCH, Pulverisette7). The obtained mixtures are pelletized, placed in a quartz tube, and sealed, followed by annealing at 550 °C for 5 hrs, and then slowly cooling to room temperature. Subsequently, the pellets are taken out from the tube and manually ground in an agate mortar to obtain the Li_6_PS_5_Cl powders.

**Preparation of Li_10_GeP_2_S_12_ (LGPS)** Li_2_S (Alfa Aesar 99.9%), P_2_S_5_ (MACKLIN ≥ 99%), and GeS_2_ (MACKLIN ≥ 99%) are mixed in an Ar-filled glovebox according to the chemistry composition. These powders were then sealed into a sealed zirconia pot and ball milled for 4 hrs using a milling apparatus (FRITSCH, Pulverisette7). Subsequently, the powders are poured out into an agate mortar and ground again. The ground powders are then pressed into pellets, sealed in a tube (Ar-filled), and heated at 550 °C for 8 hrs. After that, the pellets are taken out from the tube and ball milled again to obtain the fine LGPS powders.

**Preparation of LPS7228 film** 99.5 wt% solid electrolytes and 0.5 wt% PTFE are weighed and placed in an agate mortar and thoroughly mixed until a dough is formed. Then the malleable dough is sandwiched by two pieces of stainless-steel foil and calendared into a membrane with a desirable thickness.

**Preparation of μSi anode** To prepare the 99.7 wt% µSi anode, a slurry is prepared using the µSi particles (MACKLIN 99.9%), N-Methyl-2-Pyrrolidone (NMP, Alfa) solvent and 0.3 wt% of the polyvinylidene fluoride binder (PVDF, Sigma), before casting on a copper current collector using a doctor blade. The rough side of the copper foil is used to improve adhesion. The casted electrode is left to dry under vacuum at 85 °C overnight to remove the solvent, before punching to obtain the electrode discs.

**Supplementary References**

1. Kudu, Ö. U. *et al.* A review of structural properties and synthesis methods of solid electrolyte materials in the Li2S − P2S5 binary system. *J. Power Sources* **407**, 31-43 (2018).

2. Dietrich, C. *et al.* Lithium ion conductivity in Li2S–P2S5 glasses – building units and local structure evolution during the crystallization of superionic conductors Li3PS4, Li7P3S11 and Li4P2S7. *J. Mater. Chem. A* **5**, 18111-18119 (2017).

3. Lu, S. *et al.* Optimization of lithium ion conductivity of Li2S-P2S5 glass ceramics by microstructural control of crystallization kinetics. *Solid State Ionics* **362**, 115583 (2021).

4. Liu, Z. *et al.* Anomalous High Ionic Conductivity of Nanoporous β-Li3PS4. *J. Am. Chem. Soc.* **135**, 975-978 (2013).

5. Zhao, F. *et al.* An Air-Stable and Li-Metal-Compatible Glass-Ceramic Electrolyte enabling High-Performance All-Solid-State Li Metal Batteries. *Adv. Mater.* **33**, 2006577 (2021).

6. Jiang, Z. *et al.* Improved Ionic Conductivity and Li Dendrite Suppression Capability toward Li7P3S11-Based Solid Electrolytes Triggered by Nb and O Cosubstitution. *ACS Appl. Mater. Interfaces* **12**, 54662-54670 (2020).

7. Ahmad, N. *et al.* Enhanced Air Stability and High Li-Ion Conductivity of Li6.988P2.994Nb0.2S10.934O0.6 Glass–Ceramic Electrolyte for All-Solid-State Lithium–Sulfur Batteries. *ACS Appl. Mater. Interfaces* **12**, 21548-21558 (2020).

8. Zhou, L. *et al.* Cathode-doped sulfide electrolyte strategy for boosting all-solid-state lithium batteries. *Chem. Eng. J.* **391**, 123529 (2020).

9. Zhao, B.-S. *et al.* Congener Substitution Reinforced Li7P2.9Sb0.1S10.75O0.25 Glass-Ceramic Electrolytes for All-Solid-State Lithium–Sulfur Batteries. *ACS Appl. Mater. Interfaces* **13**, 34477-34485 (2021).

10. Liu, G. *et al.* High air-stability and superior lithium ion conduction of Li3+3xP1-xZnxS4-xOx by aliovalent substitution of ZnO for all-solid-state lithium batteries. *Energy Storage Materials* **17**, 266-274 (2019).

11. Zhou, L. *et al.* Strong Interfacial Adhesion between the Li2S Cathode and a Functional Li7P2.9Ce0.2S10.9Cl0.3 Solid-State Electrolyte Endowed Long-Term Cycle Stability to All-Solid-State Lithium–Sulfur Batteries. *ACS Appl. Mater. Interfaces* **13**, 28270-28280 (2021).

12. Zhao, F. *et al.* An Air-Stable and Li-Metal-Compatible Glass-Ceramic Electrolyte enabling High-Performance All-Solid-State Li Metal Batteries. *Adv. Mater.* **33**, e2006577 (2021).

13. Zhao, F. *et al.* Ultrastable Anode Interface Achieved by Fluorinating Electrolytes for All-Solid-State Li Metal Batteries. *ACS Energy Lett.* **5**, 1035-1043 (2020).

14. Zhao, F. *et al.* A Versatile Sn-Substituted Argyrodite Sulfide Electrolyte for All-Solid-State Li Metal Batteries. *Adv. Energy Mater.* **10**, 1903422 (2020).

15. Xu, R. *et al.* Interface engineering of sulfide electrolytes for all-solid-state lithium batteries. *Nano Energy* **53**, 958-966 (2018).

16. Liang, J. *et al.* An Air-Stable and Dendrite-Free Li Anode for Highly Stable All-Solid-State Sulfide-Based Li Batteries. *Adv. Energy Mater.* **9**, 1902125 (2019).

17. Zhang, Z. *et al.* Interface Re-Engineering of Li10GeP2S12 Electrolyte and Lithium anode for All-Solid-State Lithium Batteries with Ultralong Cycle Life. *ACS Appl. Mater. Interfaces* **10**, 2556-2565 (2018).

18. Park, H. *et al.* Epitaxial Growth of Nanostructured Li2Se on Lithium Metal for All Solid-State Batteries. *Adv. Sci.* **8**, 2004204 (2021).

19. Wang, C. *et al.* In Situ Ion-Conducting Protective Layer Strategy to Stable Lithium Metal Anode for All-Solid-State Sulfide-Based Lithium Metal Batteries. *Adv. Mater. Interfaces* **8**, 2001698 (2021).

20. Choi, Y. E. *et al.* Coatable Li4SnS4 Solid Electrolytes Prepared from Aqueous Solutions for All-Solid-State Lithium-Ion Batteries. *ChemSusChem* **10**, 2605-2611 (2017).

21. Zhang, W. *et al.* Interfacial Processes and Influence of Composite Cathode Microstructure Controlling the Performance of All-Solid-State Lithium Batteries. *ACS Appl. Mater. Interfaces* **9**, 17835-17845 (2017).

22. Kato, Y. *et al.* High-power all-solid-state batteries using sulfide superionic conductors. *Nat. Energy* **1**, 16030 (2016).

23. Kato, Y. *et al.* All-Solid-State Batteries with Thick Electrode Configurations. *J. Phys. Chem. Lett.* **9**, 607-613 (2018).

24. Randau, S. *et al.* Benchmarking the performance of all-solid-state lithium batteries. *Nat. Energy* **5**, 259-270 (2020).

25. Xu, X. *et al.* Self-Organized Core–Shell Structure for High-Power Electrode in Solid-State Lithium Batteries. *Chem. Mater.* **23**, 3798-3804 (2011).

26. Xu, R. *et al.* Cathode-Supported All-Solid-State Lithium–Sulfur Batteries with High Cell-Level Energy Density. *ACS Energy Lett.* **4**, 1073-1079 (2019).

27. Whiteley, J. M., Taynton, P., Zhang, W. & Lee, S.-H. Ultra-thin Solid-State Li-Ion Electrolyte Membrane Facilitated by a Self-Healing Polymer Matrix. *Adv. Mater.* **27**, 6922-6927 (2015).

28. Nam, Y. J. *et al.* Bendable and Thin Sulfide Solid Electrolyte Film: A New Electrolyte Opportunity for Free-Standing and Stackable High-Energy All-Solid-State Lithium-Ion Batteries. *Nano Lett.* **15**, 3317-3323 (2015).

29. Seino, Y., Ota, T., Takada, K., Hayashi, A. & Tatsumisago, M. A sulphide lithium super ion conductor is superior to liquid ion conductors for use in rechargeable batteries. *Energy Environ. Sci.* **7**, 627-631 (2014).

30. Kuhn, A. *et al.* In Search of Aluminum Hexathiohypodiphosphate: Synthesis and Structures of ht-AlPS4, lt-AlPS4, and Al4(P2S6)3. *Z. Anorg. Allg. Chem.* **640**, 2663-2668 (2014).

31. Stöffler, H. *et al.* Li+-Ion Dynamics in β-Li3PS4 Observed by NMR: Local Hopping and Long-Range Transport. *J. Phys. Chem. C* **122**, 15954-15965 (2018).

32. Dietrich, C. *et al.* Local Structural Investigations, Defect Formation, and Ionic Conductivity of the Lithium Ionic Conductor Li4P2S6. *Chem. Mater.* **28**, 8764-8773 (2016).

33. Koerver, R. *et al.* Capacity Fade in Solid-State Batteries: Interphase Formation and Chemomechanical Processes in Nickel-Rich Layered Oxide Cathodes and Lithium Thiophosphate Solid Electrolytes. *Chem. Mater.* **29**, 5574-5582 (2017).

34. Zhong, H., Wu, Y., Ding, F., Sang, L. & Mai, Y. An artificial Li-Al interphase layer on Li-B alloy for stable lithium-metal anode. *Electrochim. Acta* **304**, 255-262 (2019).

35. Kim, H. *et al.* Enhancing performance of Li–S cells using a Li–Al alloy anode coating. *Electrochem. Commun.* **36**, 38-41 (2013).

36. Wang, L. *et al.* In situ formation of a LiF and Li–Al alloy anode protected layer on a Li metal anode with enhanced cycle life. *J. Mater. Chem. A* **8**, 1247-1253 (2020).

37. Han, F., Zhu, Y., He, X., Mo, Y. & Wang, C. Electrochemical Stability of Li10GeP2S12 and Li7La3Zr2O12 Solid Electrolytes. *Adv. Energy Mater.* **6**, 1501590 (2016).
